# An atropisomeric M_2_L_4_ cage mixture displaying guest-induced convergence and strong guest emission in water†

**DOI:** 10.1039/d0sc03223a

**Published:** 2020-07-20

**Authors:** Takahiro Tsutsui, Lorenzo Catti, Kenji Yoza, Michito Yoshizawa

**Affiliations:** Laboratory for Chemistry and Life Science, Institute of Innovative Research, Tokyo Institute of Technology 4259 Nagatsuta, Midori-ku Yokohama 226-8503 Japan yoshizawa.m.ac@m.titech.ac.jp; WPI Nano Life Science Institute, Kanazawa University Kakuma-machi Kanazawa 920-1192 Japan; Bruker AXS 3-9 Moriya-cho, Kanagawa-ku Yokohama 221-0022 Japan

## Abstract

Introduction of atropisomeric axes into a bent bispyridine ligand leads to the quantitative formation of a complex mixture of atropisomeric M_2_L_4_ cages upon treatment with metal ions. Whereas the isomer ratio of the obtained cage mixture, consisting of up to 42 isomers, is insensitive to temperature and solvent, the quantitative convergence from the mixture to a single isomer is accomplished upon encapsulation of a large spherical guest, namely fullerene C_60_. The observed isomerization with other guests depends largely on their size and shape (*e.g.*, <10 and 82% convergence with planar triphenylene and bowl-shaped corannulene guests, respectively). Besides the unusual guest-induced convergence, the present cage mixture displays the strongest guest emission (*Φ*_F_ = 68%) among previously reported M_*n*_L_*m*_ cages and capsules, upon encapsulation of a BODIPY dye in water.

## Abstract 2

A complex mixture of atropisomeric M_2_L_4_ cages is shown to undergo perfect convergence to a single isomer upon encapsulation of spherical C_60_ in water. Moreover, the cage mixture displays very strong guest emission upon encapsulation of a BODIPY dye.

## Graphical abstract

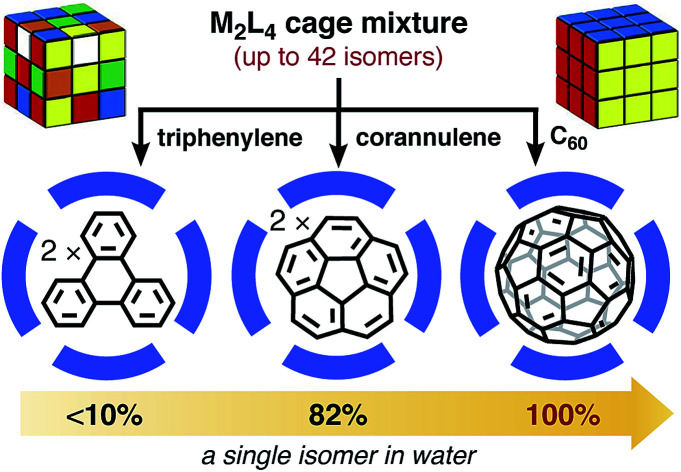

## Introduction

**Fig. 1:**
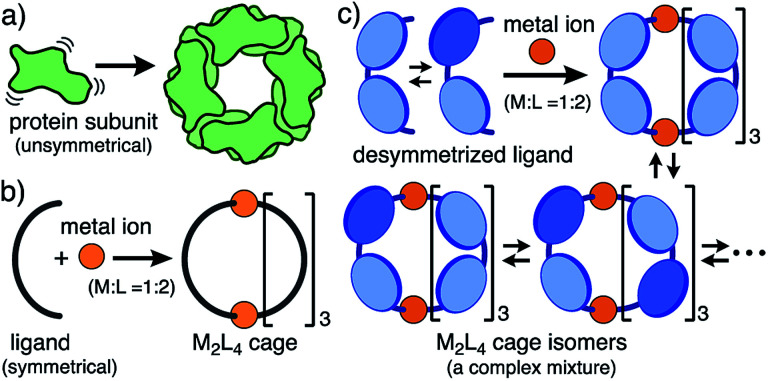
Schematic representation of the self-assemblies of (a) a biological nanostructure, (b) an M_2_L_4_ cage from symmetrical ligands and metal ions, and (c) a complex mixture of M_2_L_4_ cage isomers from desymmetrized ligands and metal ions studied herein.

Biological assemblies such as viral capsids and ferritins display well-organized, spherical nanoarchitectures yet consist of unsymmetrical protein subunits bearing flexible moieties before assembly (Fig. 1a).^1^ On the other hand, synthetic chemists are apt to design highly symmetrical subunits with sufficient conformational rigidity to precisely construct spherical nanostructures through hydrogen bonding^2^ and coordination-driven self-assemblies,^3^*e.g.*, a spherical M_2_L_4_ cage (Fig. 1b).^4^ The cavity size and properties of such artificial assemblies are also highly controllable by the use of the well-designed components.^5^ In contrast, desymmetrized, synthetic subunits with one or more isomerizable moieties give rise to a complex mixture of product isomers *via* self-assembly (*e.g.*, Fig. 1c). So far, no rational method has been reported to converge such complex, artificial mixtures (so-called dynamic combinatorial libraries)^6^ into a single, three-dimensional isomer.

**Fig. 2:**
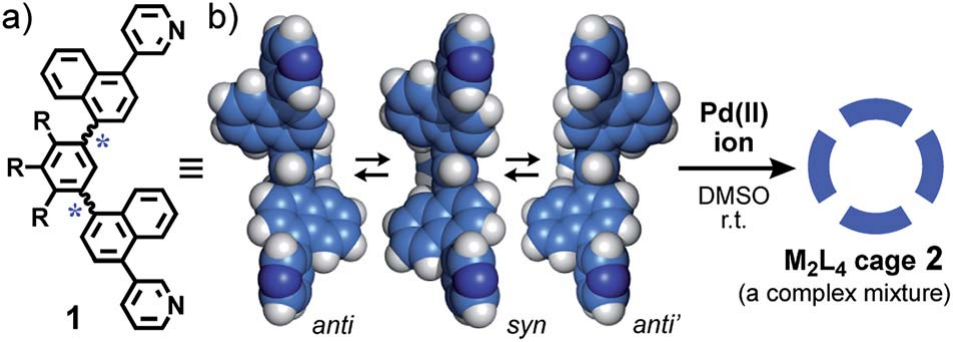
Schematic representation of (a) bispyridine ligand **1** (R = –OCH_2_CH_2_OCH_3_) and, (b) the optimized structures of the atropisomers in equilibrium (DFT calculation, B3LYP/6-31* level, R = –OCH_3_) and the formation of M_2_L_4_ cage **2** as a complex isomeric mixture.

Herein we report the design and synthesis of bent bispyridine ligand **1** with *two atropisomeric axes* (Fig. 2a). Treatment of the isomerizable and desymmetrized ligands with metal ions leads to the quantitative formation of a complex mixture of atropisomeric M_2_L_4_ cages **2** (Fig. 2b). Although the isomer ratio of the resultant cages is insensitive to temperature and solvent, the selective and quantitative convergences from the complex mixture to a single isomer are demonstrated upon encapsulation of spherical corannulene dimer and fullerene C_60_, respectively. In addition, the present cage mixture displays the strongest guest emission (*Φ*_F_ = 68%), as compared to previously reported M_*n*_L_*m*_ cages and capsules,^7^ upon encapsulation of a fluorescent BODIPY dye.

**Fig. 3:**
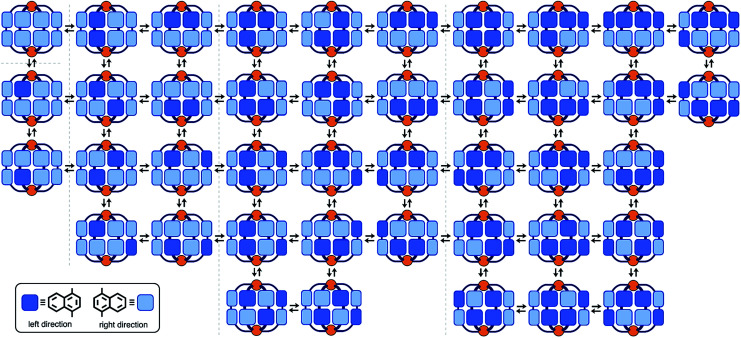
Schematic representation of the possible 42 isomers of M_2_L_4_ cage **2**. These isomers are in equilibrium in solution.

As the simplest model, we chose a highly symmetrical M_2_L_4_ structure to examine its desymmetrization and convergence. It has been established that bent bispyridine ligands with a virtual *C*_2v_ symmetry generate M_2_L_4_ cages or capsules as a single isomer upon complexation with metal ions (Fig. 1b).^4^ Their host abilities have been widely investigated by many groups so far.^8,9^ Thus we introduced atropisomeric axes^10^ into the ligand and designed 1,4-naphthylene-embedded, bent ligand **1** (Fig. 2a). The new bispyridine ligand adopts three atropisomers owing to hindered rotation around the phenyl–naphthyl bonds so that the corresponding atropisomeric M_2_L_4_ cage, possessing a spherical hydrophobic cavity (∼1 nm in diameter), is anticipated to be present as an equilibrium mixture of up to 42 isomers (Fig. 3).

## Results and discussion

### Formation of a complex mixture of M_2_L_4_ cages

**Fig. 4:**
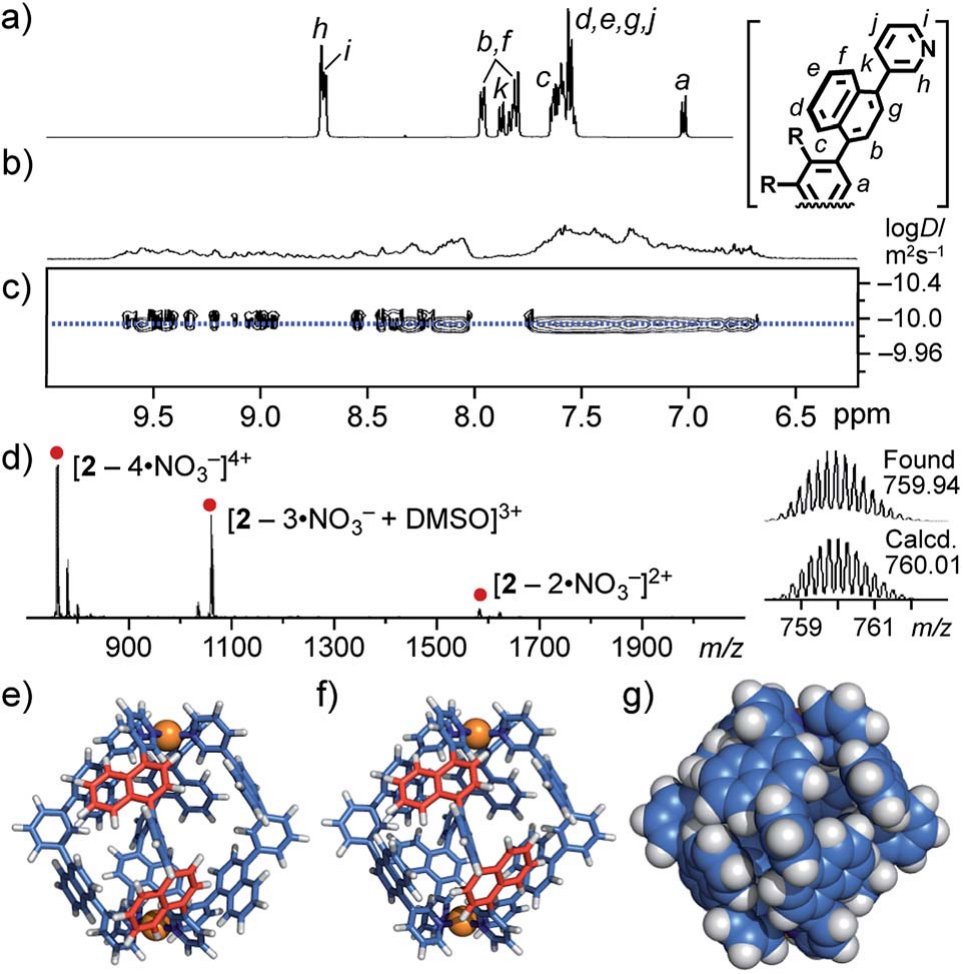
^1^H NMR spectra (500 MHz, DMSO-*d*_6_, r.t.) of (a) ligand **1** and (b) a complex mixture of cage isomers **2**. (c) ^1^H DOSY NMR (500 MHz, DMSO-*d*_6_, 298 K) and (d) ESI-TOF MS (DMSO, r.t.) spectra of isomers **2**. Optimized structures of **2**: (e) an all-*syn* isomer and (f and g) an all-*anti* isomer (substituents and counterions are omitted for clarity).

First we observed the quantitative formation of a complex mixture of M_2_L_4_ cage **2** isomers by mixing atropisomeric ligand **1**, PdCl_2_(DMSO)_2_, and AgNO_3_ (in a 2 : 1 : 2 ratio) in DMSO-*d*_6_ for 1 h at room temperature (Fig. 2).^11,12*a*^ As expected, the ^1^H NMR spectrum of the product mixture showed complicated and broadened, aromatic signals in the range of 9.7 to 6.6 ppm, in contrast to the simple and sharp signals observed for free ligand **1** (Fig. 4a and b). The ^1^H DOSY NMR spectrum revealed that these proton signals have the same diffusion constant (*D* = 1.2 × 10^−10^ m^2^ s^−1^; Fig. 4c),^12*b*^ indicating the existence of Pd(ii)-linked cage **2** as an isomeric mixture in equilibrium. The exclusive formation of M_2_L_4_ assemblies **2** was unambiguously confirmed by ESI-TOF MS analysis: prominent peaks were observed at *m*/*z* 759.9, 1033.9, and 1582.3, assignable to the [**2** – *n*·NO_3_^−^]^*n*+^ species (*n* = 4, 3, and 2, respectively; Fig. 4d). In a manner similar to **2**, Pt(ii)-analogue **2′** was formed quantitatively and its M_2_L_4_ structure was confirmed by NMR and MS analyses.^11^ The existence of various isomers of **2′** was also established by the complicated and broadened ^1^H NMR signals (Fig. S23†).

The theoretical calculation of cage **2** indicated that differences in energy are relatively small among representative isomers (Δ*E* < 1.8 kcal mol^−1^; *e.g.*, Fig. 4e, f and S22†),^11^ supporting the complex signals observed in the ^1^H NMR spectrum (Fig. 4b). The optimized structure of **2** (*e.g.*, an all-*anti* isomer, Fig. 4g) clarified that the spherical cavity (∼580 Å^3^) is surrounded by eight naphthalene panels and the cavity diameter is 1.3 nm. Although the cavity shape and size are almost identical to those of anthracene-based M_2_L_4_ capsule **3** reported previously (Fig. S1†),^9,11^ the bent polyaromatic ligands of **2** are proximate to each other yet unstacked.

### Guest-induced convergence to a single isomer

**Fig. 5:**
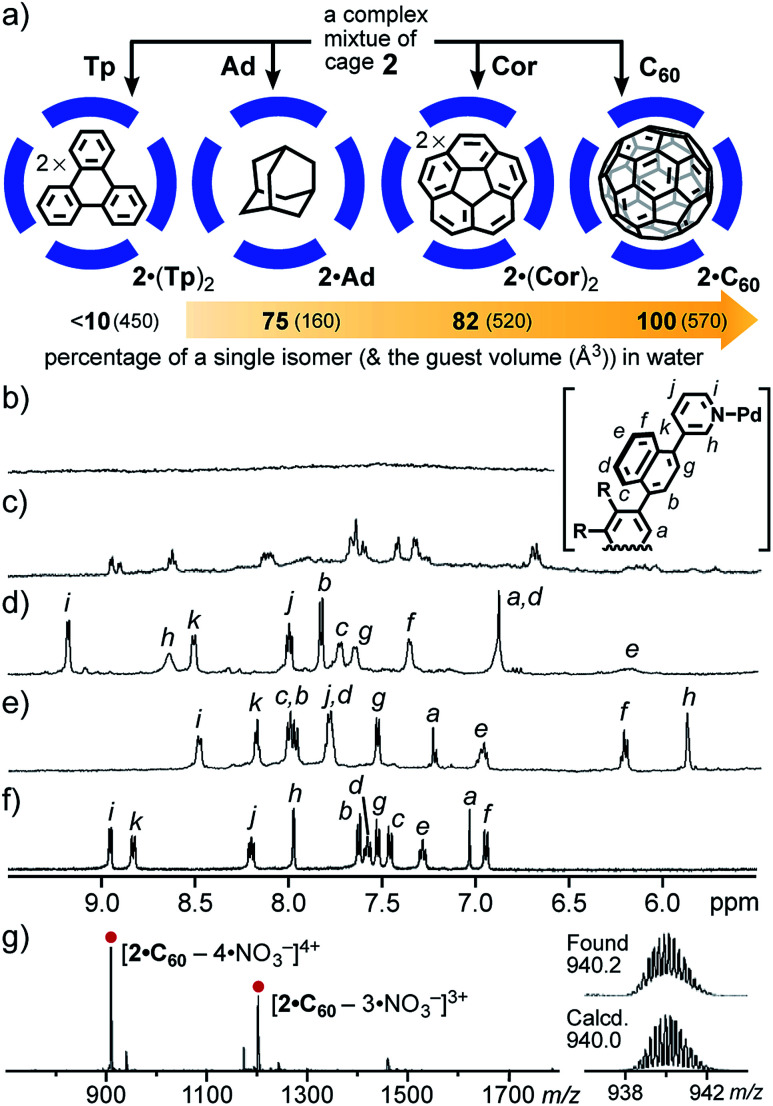
(a) Schematic representation of the guest-induced convergence of a complex mixture of **2**. ^1^H NMR spectra (500 MHz, D_2_O, r.t.) of (b) cage **2**, (c) **2**·(**Tp**)_2_, (d) **2**·**Ad**, (e) **2**·(**Cor**)_2_, and (f) **2**·**C60**. (g) ESI-TOF MS spectrum (H_2_O) of **2**·**C60** and the expansion and simulation of the [**2**·**C60** – 4·NO_3_^−^]^4+^ signals.

Whereas the isomer ratio of cage **2** remained intact even upon changing the solvent (*e.g.*, CD_3_CN, CD_3_OD, and D_2_O) and temperature (*e.g.*, −10 to 110 °C), guest encapsulation allowed the complex mixture to convert into a single isomer in water. When a simple planar and hydrophobic guest, triphenylene (**Tp**; excess), was mixed with a D_2_O solution of **2** (0.6 mM) at room temperature for 1 h (Fig. 5a), the ^1^H NMR signals of **2** changed from completely broad^13^ to slightly sharp (Fig. 5b and c), due to the quantitative incorporation of the guests (2 equiv.) into the host cavity through the hydrophobic effect.^14^ The NMR signals were unchanged even at elevated temperature (*e.g.*, 80 °C). The ESI-TOF MS analysis of the product clearly supported the formation of 1 : 2 host–guest complex **2**·(**Tp**)_2_ (Fig. S27c†). This result indicated the possibility of wide-ranging host abilities of **2** in water and selective isomerization of **2** through an appropriate guest stimulus.^15^

Next, the treatment of small spherical adamantane (**Ad**) and bowl-shaped corannulene (**Cor**) with cage mixture **2** gave rise to host–guest complexes **2**·**Ad** and **2**·(**Cor**)_2_ in a quantitative manner, respectively, under the same aqueous conditions (Fig. 5a).^15^ Interestingly, the resultant ^1^H NMR spectra revealed further conversion from the complex mixtures to a single isomer upon binding of the sterically demanding guests, whose shapes are similar to the spherical cavity of **2**. The percentages of the generated, major host isomer accommodating **Ad** and (**Cor**)_2_ guests were estimated to be 75 and 82, respectively (Fig. 5d and e), on the basis of the reference proton signal of the methoxy groups of **2** around 2.96–2.83 ppm. The aliphatic proton signals of bound **Ad** appeared around −0.7 ppm, due to the aromatic shielding effect of **2** (Fig. S28a†). The eleven aromatic signals were clearly observed in the ^1^H NMR spectrum of **2**·(**Cor**)_2_ (Fig. 5e). Noteworthy, a similar ^1^H NMR spectrum was obtained from a mixture of cage **2′**, which is an analogue of **2** with Pt(ii) ions, and excess **Cor** in D_2_O at room temperature for 7 h (Fig. S30†).^11^ The finding revealed that the naphthalene panels embedded in the spherical framework of **2** can flip even at ambient temperature, because the bonds between the Pt(ii) hinges and the pyridyl groups are fixed under these conditions.

The quantitative conversion of the mixture into a single isomer was accomplished upon incorporation of spherical fullerene C_60_ (C_60_) into cage **2**. Mixing of cage **2** with excess **C60** in D_2_O at 100 °C for 24 h yielded 1 : 1 host–guest complex **2**·**C60** exclusively. Intense ESI-TOF MS peaks at *m*/*z* = 940.2 and 1274.2 were assignable to [**2**·**C60** – *n*·NO_3_^−^]^*n*+^ species (*n* = 4 and 3; Fig. 5g). Notably, the ^1^H NMR spectrum of **2**·**C60** exhibited only eleven signals in the aromatic region, indicating the presence of a single isomer (∼100% selectivity; Fig. 5f). In the ^13^C NMR spectrum of **2**·**C60**, a single signal for the **C60** guest was observed at 140.1 ppm (Fig. S32†), in a manner similar to that of the previous **C60**-incorporated M_2_L_4_ capsule (141.2 ppm).^9*a*^ The broadened, new UV-visible absorption bands derived from the guest were also found around 400–730 nm (Fig. S36†).^16^ In short, these results revealed that the “atropisomeric” dynamic combinatorial library^6^ of cage **2** can be strictly controlled by its size (volume) and shape complementary guest molecules.

### Crystallographic analysis of the single isomer

**Fig. 6:**
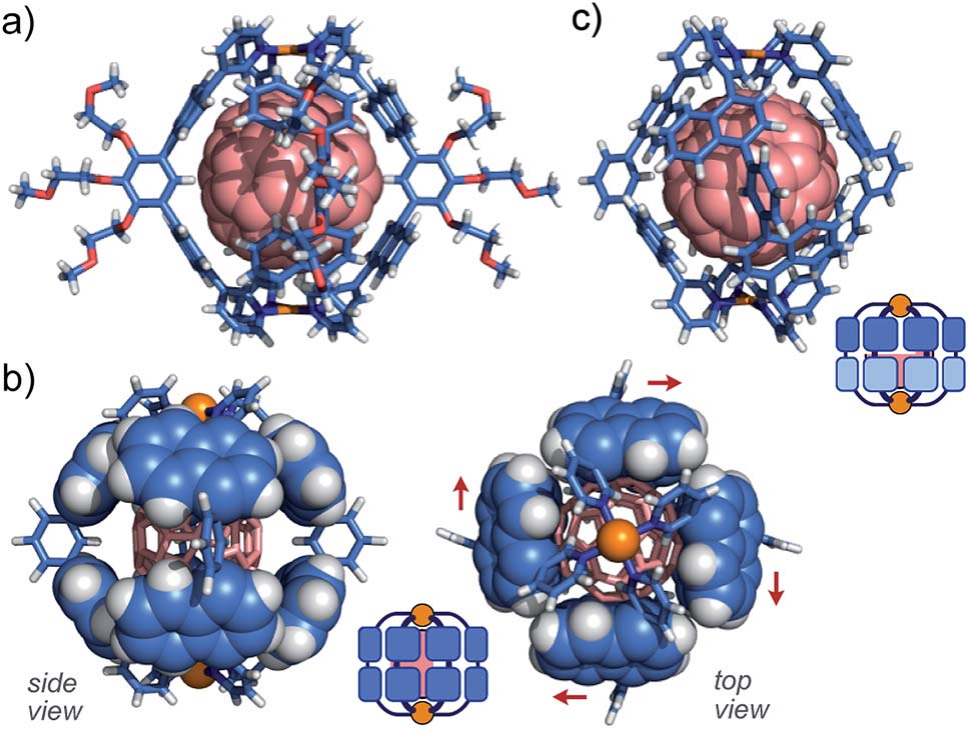
X-ray crystal structure of **2**·**C60** (all-*syn* isomer): (a) space-filling representation for **C60** and (b) space-filling representation for the naphthalene panels (the substituents are replaced by H atoms for clarity). (c) Optimized structure of **2**·**C60** (all-*anti* isomer, R = –H).

The direction of the eight naphthalene panels on **2**·**C60** was eventually determined by X-ray crystallographic and theoretical analyses.^11,17,18^ As we expected, the crystal structure showed that one molecule of **C60** is fully captured by the cage-shaped M_2_L_4_ framework of **2** (Fig. 6a). Remarkably, all the bent bispyridine ligands adopted a *syn* conformation and aligned roundly in the same direction (Fig. 6b), most probably to avoid the steric repulsion between the naphthalene panels of the adjacent ligands. The π-stacking interactions between **C60** and the naphthalene panels were observed at eight positions (*d* = 3.2–3.4 Å in interplanar distance; Fig. S38†). The theoretical calculations of **2**·**C60** (R = –H) on the basis of the present crystal structure indicated that the energy of the all-*syn* isomer (Fig. 6a) is 10.6 kcal mol^−1^ lower than that of an all-*anti* isomer (Fig. 6c) in the gas phase. The cage framework possesses four small openings (approximately 4 × 5 Å^2^) between the polyaromatic ligands, in contrast to previous M_2_L_4_ capsule **3** with an anthracene-based, closed shell.^9^ Preliminary host–guest studies showed the selective binding of one molecule of functionalized fullerenes (*e.g.*, diethyl malonate-derivatized **C60**) by the present cage in water (Fig. S40†).^11^

### Strong emission of an encapsulated BODIPY derivative

**Fig. 7:**
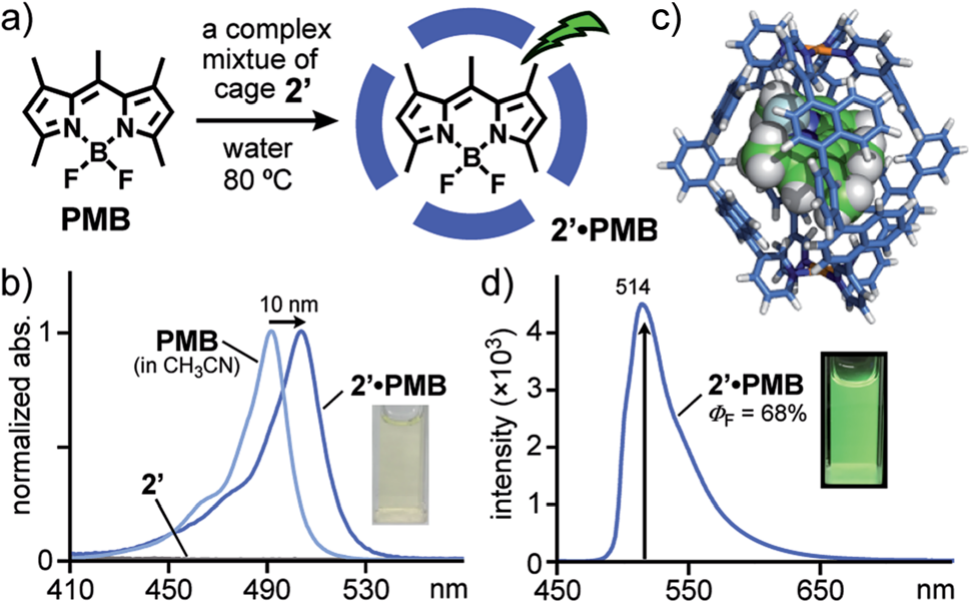
(a) Schematic representation of the formation of highly emissive host–guest complex **2′**·**PMB**. (b) UV-visible spectra and photograph (H_2_O, r.t.) of **2′**·**PMB**, **2′**, and **PMB** (in CH_3_CN). (c) Optimized structure of **2′**·**PMB** (R = –H, all-*syn* isomer). (d) Fluorescence spectrum (H_2_O, r.t., 80 μM based on **2′**, *λ*_ex_ = 500 nm) of **2′**·**PMB** and its photograph (*λ*_ex_ = 365 nm).

Finally, we encountered that naphthalene-based Pt(ii)-cage **2′** displays very strong guest emission upon encapsulation of a BODIPY dye in water.^19^ After stirring a mixture of cage **2′** and water-insoluble, pentamethyl boron-dipyrromethene **PMB** (9 equiv.) in D_2_O at 80 °C for 1.5 h (Fig. 7a), the formation of 1 : 1 host–guest complex **2′**·**PMB** was evidenced by NMR, MS, and UV-visible analyses. The obtained, broad ^1^H NMR signals indicated the existence of a complex isomeric mixture including the guest dye (Fig. S41a†). The 1 : 1 host–guest ratio of the product was confirmed by the ESI-TOF MS spectrum, which shows clear MS peaks for [**2′**·**PMB** – *n*·NO_3_^−^]^*n*+^ species (*n* = 4, 3; Fig. S41b†). The UV-visible spectrum of the pale yellow solution of **2′**·**PMB** in water showed new absorption bands in the range of 410–550 nm, derived from encapsulated **PMB** (Fig. 7b). The observed bands were slightly red-shifted (Δ*λ* = 10 nm) with respect to those of free **PMB** in CH_3_CN and their intensity indicated the encapsulation efficiency being ∼25%. The optimized structure of **2′**·**PMB** (R = –H) suggested that a slightly bent **PMB** framework is fully accommodated in the polyaromatic shell of **2′** (Fig. 7c).^11^

Host–guest complexes featuring high emissivity in water have attracted considerable attention related to biomolecular sensors. However, the majority of coordination cages and capsules disturb guest emission upon encapsulation, owing to the heavy metal effect.^3–6^ Our previous studies exceptionally demonstrated strong fluorescence (*λ*_max_ = 535 nm, *Φ*_F_ = 48%) from a 1 : 1 host–guest complex, composed of anthracene-based capsule **3** and **PMB** in water.^7*c*^ Notably, the aqueous solution of **2′**·**PMB** emitted much stronger green fluorescence with a quantum yield of 68% at *λ*_max_ = 514 nm, upon irradiation at 500 nm (Fig. 7d). The band shape and maximum wavelength are similar to those of free **PMB** in ethanol (*λ*_max_ = 522 nm, *Φ*_F_ = 78%). To the best of our knowledge, this represents the strongest guest emission in coordination host compounds reported so far.^7^ As compared to capsule **3** with a relatively rigid cavity,^9^ the flexible naphthalene-based cavity of **2′** is able to adjust to the shape of the bulky **PMB** guest (Fig. 7c), which reduces the steric host–guest repulsion. Once the cage encapsulates the guest, the flexibility of the naphthalene panels is largely suppressed due to the efficient host–guest interactions. These effects might suppress emission quenching processes, observed in the case of **3**·**PMB**, to a large degree (Δ*Φ*_F_ = +20%).^19^

## Conclusions

We have challenged the perfect conversion of a complex mixture of atropisomeric M_2_L_4_ cages into a single isomer by an external stimulus. The cage mixture including up to 42 isomers was generated upon incorporation of two atropisomeric axes into the bent bispyridine ligands of a common and highly symmetrical M_2_L_4_ structure. Unusual convergence of the mixed isomers was demonstrated upon encapsulation of guest molecules. Particularly, the encapsulation of a fullerene guest led to the quantitative formation of a single isomer, due to its size and shape complementarity to the spherical host cavity. Thus, the present system can be regarded as a novel dynamic combinatorial library capable of incorporating large guests up to 1 nm. The isomer structure (all-*syn*) of the M_2_L_4_ cage was fully characterized by NMR, MS, UV-visible, X-ray crystallographic, and theoretical analyses. As an additional host function, the cage mixture encapsulated a fluorescent BODIPY dye and the resultant 1 : 1 host–guest complex exhibited intense guest emission (*Φ*_F_ = ∼70%) in water. We believe that the present study is a small yet first “synthetic” step to approach large and well-organized, biological capsules, composed of multiple unsymmetrical subunits, *in vitro*.

## Conflicts of interest

There are no conflicts to declare.

## Supplementary Material

SC-011-D0SC03223A-s001

SC-011-D0SC03223A-s002
