# Supplementary material for: An atropisomeric M2L4 cage mixture displaying guest-induced convergence and strong guest emission in water
Source: Chem Sci. 2020 Jul 20;11(31):8145–50. doi: 10.1039/d0sc03223a (PMC8163439; doi:10.1039/d0sc03223a)
Supplement: SC-011-D0SC03223A-s001 [file SC-011-D0SC03223A-s001.pdf]

Electronic Supplementary Material (ESI) for Chemical Science.
This journal is © The Royal Society of Chemistry 2020

Supporting Information

An Atropisomeric M_2L_4 Cage Mixture Displaying Guest-Induced Convergence and Strong Guest Emission in Water

Takahiro Tsutsui,[a] Lorenzo Catti,[b] Kenji Yoza,[c] and Michito Yoshizawa*[a]

[a]Laboratory for Chemistry and Life Science, Institute of Innovative Research, Tokyo Institute of Technology, 4259 Nagatsuta, Midori-ku, Yokohama 226-8503, Japan

[b]WPI Nano Life Science Institute, Kanazawa University, Kakuma-machi, Kanazawa 920-1192, Japan

[c]Bruker AXS, 3-9 Moriya-cho, Kanagawa-ku, Yokohama 221-0022, Japan

Contents

- Materials, methods, and references
- Synthesis of 4-(3-pyridyl)naphthyl boronate (^{1}H & ^{13}C NMR, COSY, NOESY, HSQC, and MS spectra)
- Synthesis of ligand **1** (^{1}H & ^{13}C NMR, COSY, NOESY, HSQC, and MS spectra)
- Formation of cage **2** (^{1}H & ^{13}C NMR, DOSY, and MS spectra, optimized structures)
- Formation of cage **2’** (^{1}H & ^{13}C NMR, DOSY, and MS spectra)
- Preparation of **2**•(**Tp**)$_2$ (^{1}H NMR and MS spectra)
- Preparation of **2•Ad** (^{1}H NMR spectra)
- Preparation of **2**•(**Cor**)$_2$ and **2’**•(**Cor**)$_2$ (^{1}H NMR and MS spectra)
- Preparation of **2•C$_{60}$** (^{1}H & ^{13}C NMR, COSY, NOESY, MS, and UV-vis spectra, crystal data, optimized structures)
- Preparation of **2•MC$_{60}$** (^{1}H NMR, MS, and UV-vis spectra)
- Preparation of **2’•PMB** and **3•PMB** (^{1}H NMR, MS, UV-vis and fluorescence spectra)

Materials and methods

NMR: Bruker AVANCE-400 (400 MHz) and ASCEND-500 (500 MHz), MALDI-TOF MS: Bruker ultrafleXtreme, ESI-TOF MS: Bruker micrOTOF II, UV-vis: JASCO V-670DS, Fluorescence: Hitachi F-7000, Absolute PL quantum yield: Hamamatsu C9920-02G with an integration sphere, FT-IR: SHIMADZU IRSpirit-T, GPC: JAI LC-9225NEXT, X-ray: Bruker D8 VENTURE TXS.

DFT calculation: The three conformers of ligand **1** (R = -OCH_3) were optimized using density functional theory (DFT: Gaussian 16 Rev A.03, Gaussian, Inc.) at the B3LYP/6-31* level of theory, Molecular mechanics calculation (geometry optimization): Forcite module, Materials Studio, version 5.5.3 (Dassault Systèmes Co.).

Solvents and reagents: TCI Co., Ltd., FUJIFILM Wako Chemical Co., Kanto Chemical Co., Inc., Sigma-Aldrich Co., and Cambridge Isotope Laboratories, Inc. 1,5-Dibromo-2,3,4-tri(2-methoxyethoxy)benzene was synthesized according to ref. S1. Capsule **3** was synthesized according to ref. S2.

References

[S1] K. Yazaki, S. Noda, Y. Tanaka, Y. Sei, M. Akita, M. Yoshizawa, *Angew. Chem. Int. Ed.* **2016**, *55*, 15031–15034.

[S2] M. Yamashina, Y. Sei, M. Akita, M. Yoshizawa, *Nat. Commun.* **2014**, *5*, 4662.

[S3] M. Yamashina, M. M. Sartin, Y. Sei, M. Akita, S. Takeuchi, T. Tahara, M. Yoshizawa, *J. Am. Chem. Soc.* **2015**, *137*, 9266–9269.

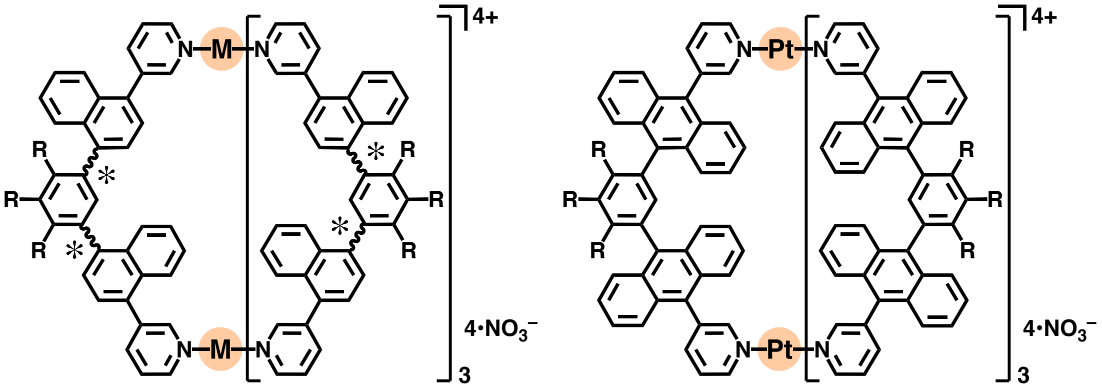

2 (M = Pd) (R = -$OCH_2CH_2OCH_3$)
2' (M = Pt)

3 (R = -$OCH_2CH_2OCH_3$)

Figure S1. Chemical structures of cages **2** and **2'**, and capsule **3**.

Synthesis of 4-(3-pyridyl)naphthyl boronate TT219, 222

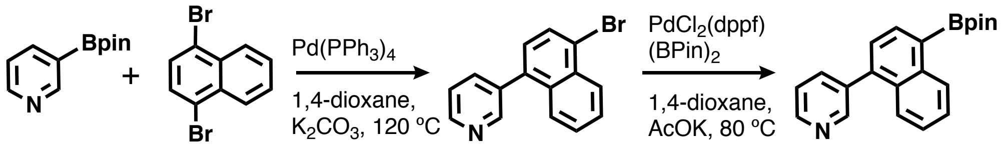

1,4-Dibromonaphthalene (2.55 g, 8.92 mmol), 3-pyridineboronic acid pinacol ester (0.921 g, 4.49 mmol), K_2CO_3 (2.31 g, 16.7 mmol), $Pd(PPh_3)_4$ (0.321 g, 0.277 mmol), and dry 1,4-dioxane (50 mL) were added to a 100 mL 2-necked glass flask filled with N_2. The resulted solution was stirred at 120 °C for 23 h. The mixture was concentrated under reduced pressure. After addition of water, the crude product was extracted with CH_2Cl_2 and then the combined organic phase was dried over $MgSO_4$, filtrated, and concentrated under reduced pressure. The crude product was purified by column chromatography (silica gel, CH_2Cl_2 : ethyl acetate = 9 : 1) to afford 1-bromo-4-(3-pyridyl)naphthalene as a white solid (0.881 g, 3.10 mmol; 69% yield).

1-Bromo-4-(3-pyridyl)naphthalene (0.881 g, 3.10 mmol), bis(pinacolato)diboron (0.879 g, 3.81 mmol), potassium acetate (0.913 g, 9.30 mmol), $PdCl_2$(dppf) (0.178 g, 0.310 mmol, dppf = 1,1'-bis(diphenylphosphino)ferrocene), and dry 1,4-dioxane (40 mL) were added to a 100 mL 2-necked glass flask filled with N_2. The resulted solution was stirred at 80 °C for 23 h. The mixture was concentrated under reduced pressure. The crude compound was passed through short column chromatography (silica gel) to remove salt and then concentrated under reduced pressure. The crude product was purified by column chromatography (silica gel, CH_2Cl_2:ethyl acetate = 9:1) and GPC to afford 4-(3-pyridyl)naphthyl boronate as a white solid (0.812 g, 2.45 mmol; 78% yield).

1-Bromo-4-(3-pyridyl)naphthalene: ^{1}H NMR (400 MHz, $CDCl_3$, r.t.): δ 7.26 (d, J = 7.4 Hz, 1H), 7.44 (dd, J = 4.9, 7.4 Hz, 1H), 7.51 (dd, J = 7.6, 7.6 Hz, 1H), 7.63 (dd, J = 7.6, 7.9 Hz, 1H), 7.77-7.79 (m, 2H), 7.86 (d, J = 7.4 Hz, 1H), 8.35 (d, J = 7.9 Hz, 1H), 8.71 (d, J = 4.9 Hz, 1H), 8.73 (s, 1H). ^{13}C NMR (100 MHz, $CDCl_3$, r.t.): δ 123.3 (CH), 123.5 (C_q), 126.1 (CH), 127.4 (CH), 127.6 (CH), 127.7 (CH), 127.9 (CH), 129.6 (CH), 132.3 (C_q), 132.9 (C_q), 135.8 (C_q), 136.6 (C_q), 137.4 (CH), 149.1 (CH), 150.6 (CH). FT-IR (KBr, cm^{-1}): 3043, 1565, 1506, 1477, 1379, 1024, 965, 850, 803, 754, 712, 622, 419. HR MS (ESI, CH_3OH): m/z Calcd. for $C_{15}H_{10}NBrNa$ [M + Na]$^+$ 305.9889, Found 305.9888.

4-(3-Pyridyl)naphthyl boronate: ^{1}H NMR (400 MHz, $CDCl_3$, r.t.): δ 1.43 (s, 12H), 7.39-7.46 (m, 3H), 7.57 (dd, J = 7.4, 8.0 Hz, 1H), 7.77-7.80 (m, 2H), 8.15 (d, J = 6.8 Hz,

1H), 8.68 (d, J = 4.0 Hz, 1H), 8.75 (s, 1H), 8.89 (d, J = 8.0 Hz, 1H). ^{13}C NMR (100 MHz, $CDCl_3$, r.t.): δ 25.0 (CH_3) × 4, 84.0 (C_q) × 2, 123.2 (CH), 125.6 (CH), 126.2 (CH), 126.6 (CH) × 2, 129.0 (CH), 131.3 (C_q), 135.1 (CH), 136.6 (C_q), 137.3 (CH), 137.4 (C_q) × 2, 139.6 (C_q), 148.7 (CH), 150.5 (CH). ESI-TOF MS (CH_3OH): m/z Calcd. for $C_{21}H_{22}BNO_2Na$ [M + Na]$^+$ 332.18, Found 332.18.

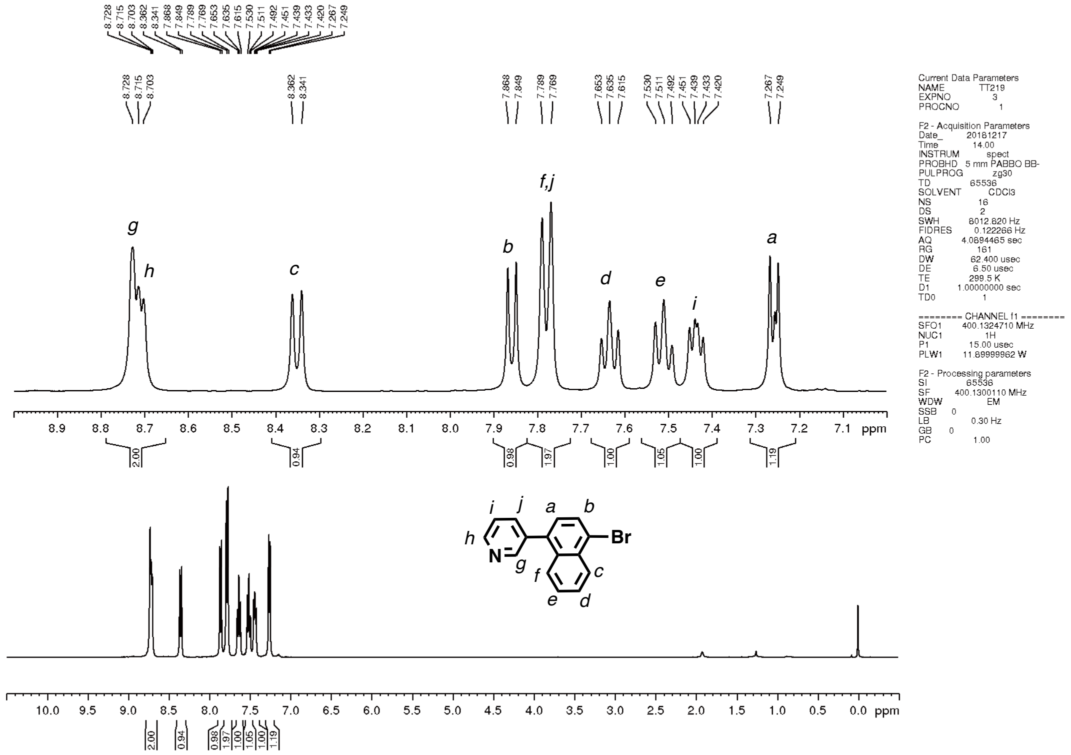

Figure S2a. ^{1}H NMR spectrum (400 MHz, $CDCl_3$, r.t.) of 1-bromo-4-(3-pyridyl)naphthalene.

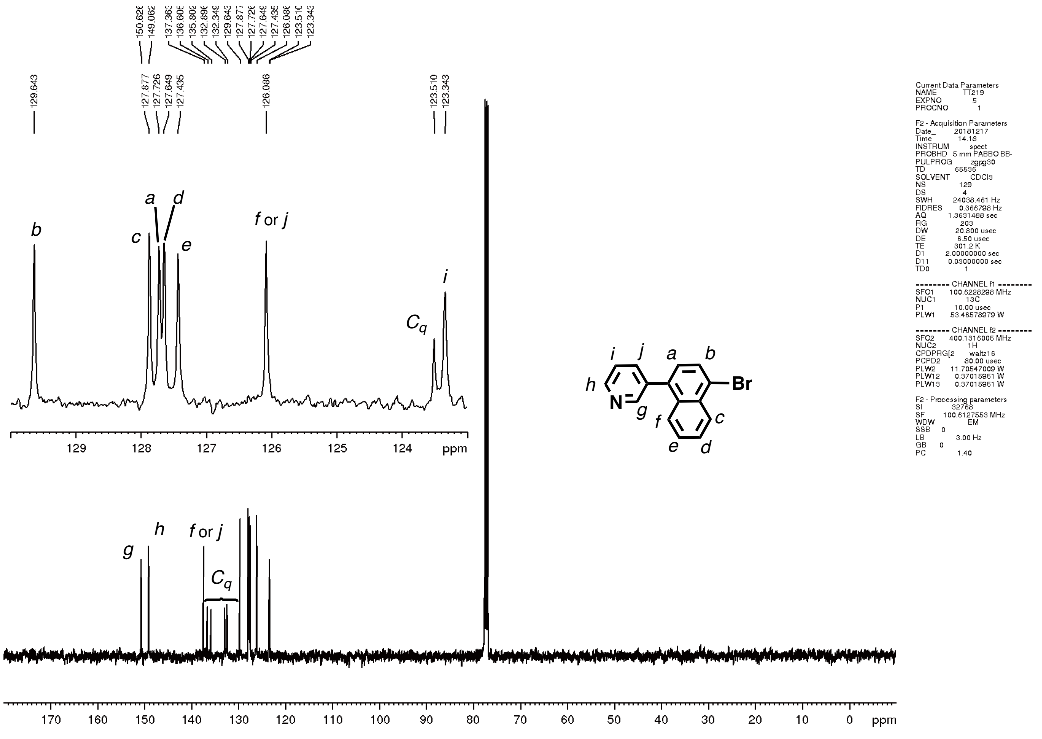

Figure S2b. ^{13}C NMR spectrum (100 MHz, $CDCl_3$, r.t.) of 1-bromo-4-(3-pyridyl)naphthalene.

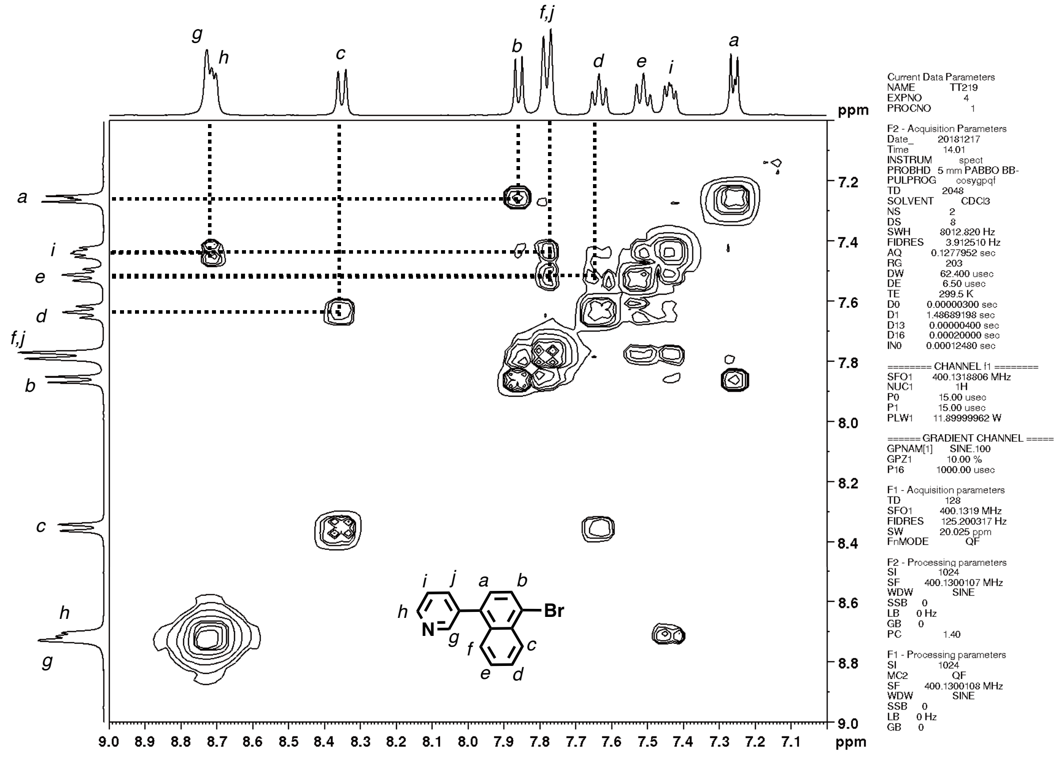

Figure S3. ^{1}H-^{1}H COSY spectrum (400 MHz, $CDCl_3$, r.t.) of 1-bromo-4-(3-pyridyl)naphthalene.

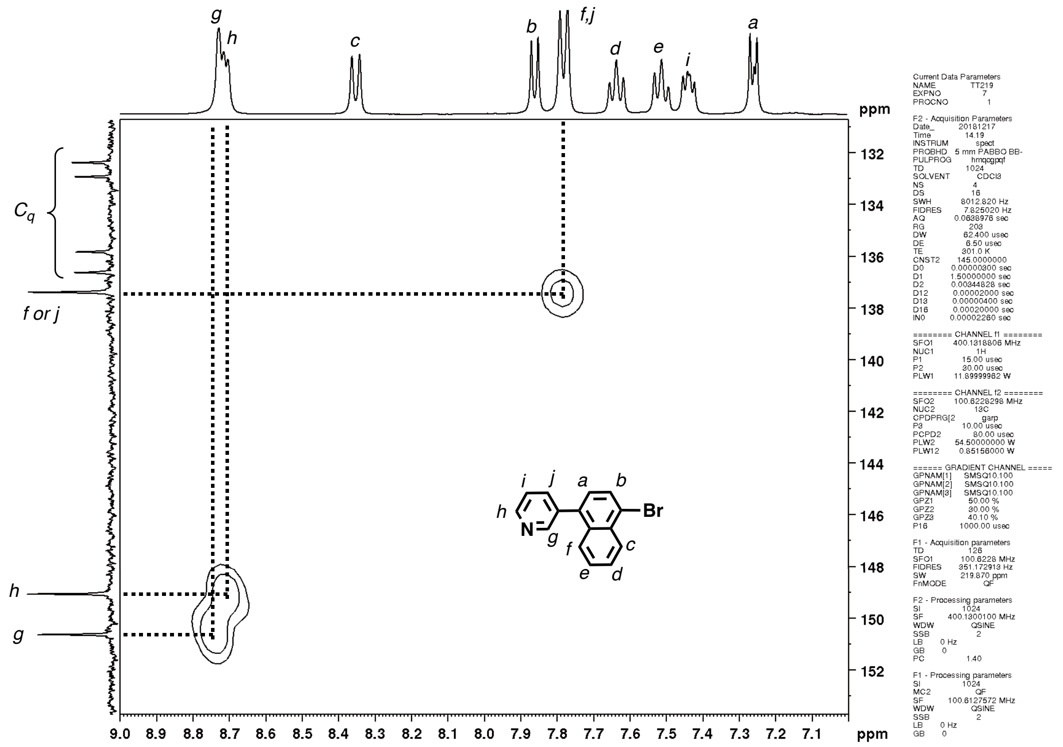

Figure S4a. HSQC spectrum (400 MHz, $CDCl_3$, r.t.) of 1-bromo-4-(3-pyridyl)naphthalene.

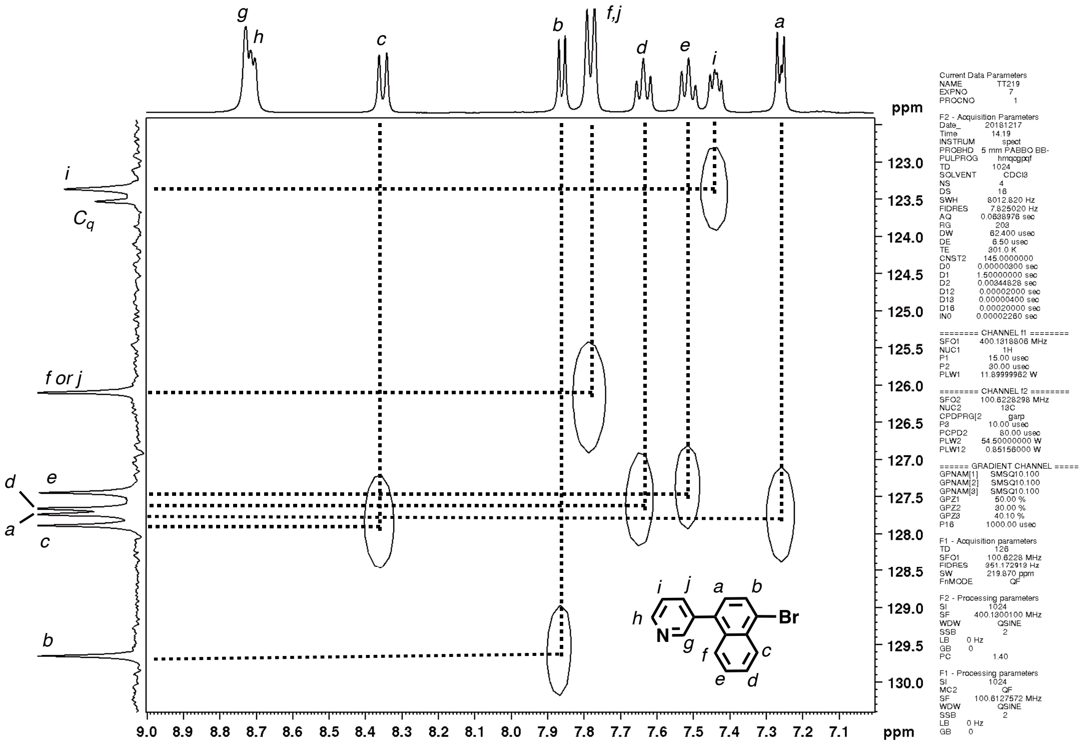

Figure S4b. HSQC spectrum (400 MHz, $CDCl_3$, r.t.) of 1-bromo-4-(3-pyridyl)naphthalene.

Analysis Info

Analysis Name	D:\Data\akita\17Tsutsui\TT219\1-bromo-4-(3-pyridyl)naphthalene_Me.d	Acquisition Date	2019/01/16 19:39:16
Method	esi_posi_wide.m	Operator	BDAL@DE
Sample Name	1-bromo-4-(3-pyridyl)naphthaleneMe	Instrument	micrOTOF 213750.10321
Comment			

Acquisition Parameter

Source Type	ESI	Ion Polarity	Positive	Set Nebulizer	0.3 Bar
Focus	Not active			Set Dry Heater	180 • ‹C
Scan Begin	50 m/z	Set Capillary	4500 V	Set Dry Gas	4.0 l/min
Scan End	2000 m/z	Set End Plate Offset	-500 V	Set Divert Valve	Waste

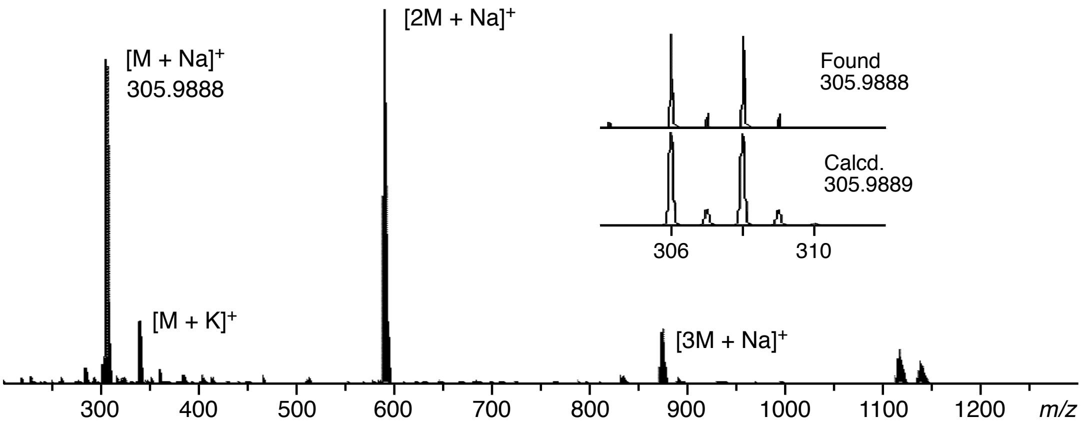

Figure S5. HR MS spectrum (ESI, CH_3OH) of 1-bromo-4-(3-pyridyl)naphthalene.

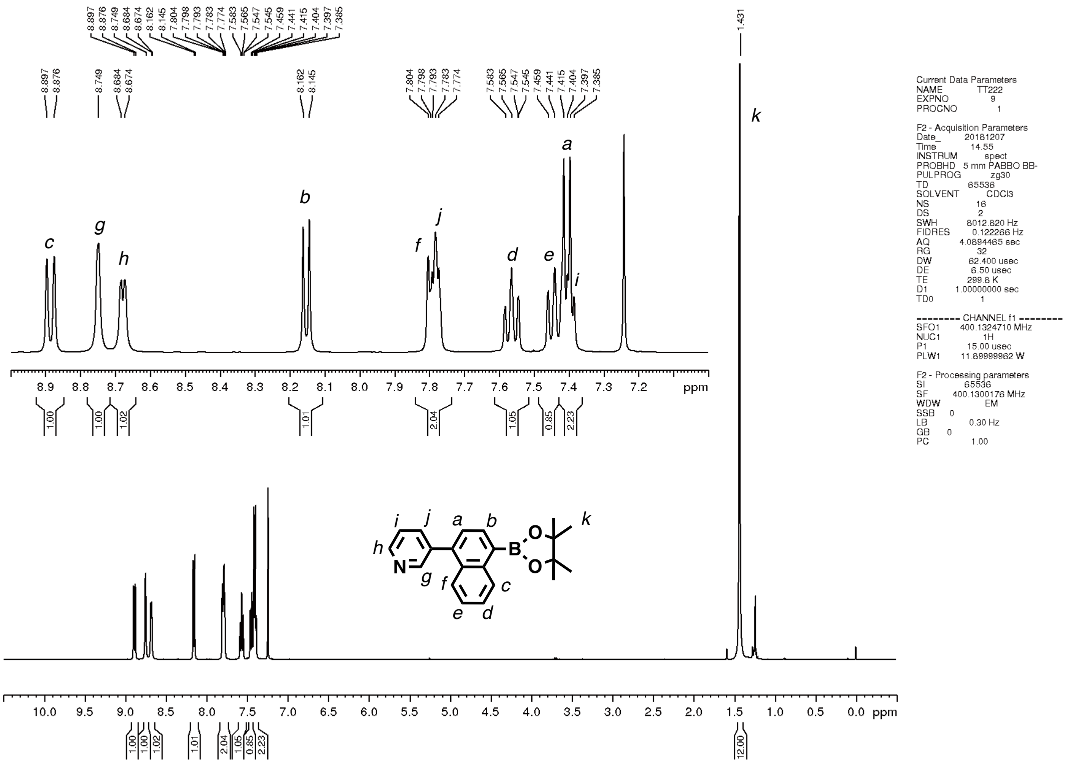

Figure S6. ^{1}H NMR spectrum (400 MHz, $CDCl_3$, r.t.) of 4-(3-pyridyl)naphthyl boronate.

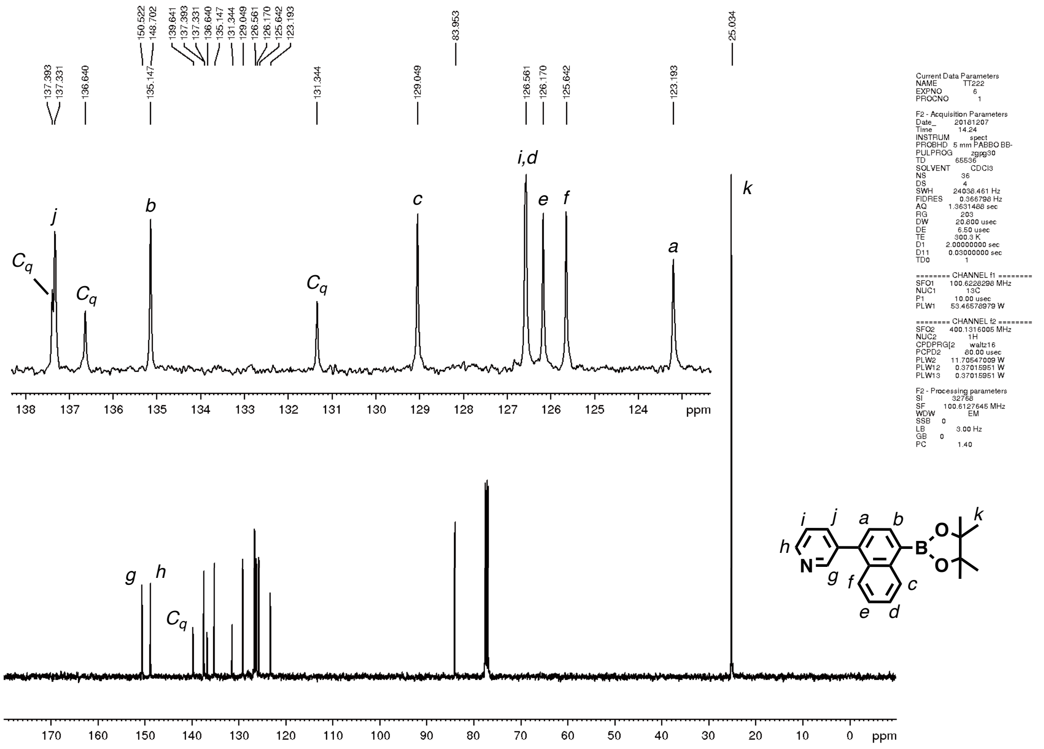

Figure S7. ^{13}C NMR spectrum (100 MHz, $CDCl_3$, r.t.) of 4-(3-pyridyl)naphthyl boronate.

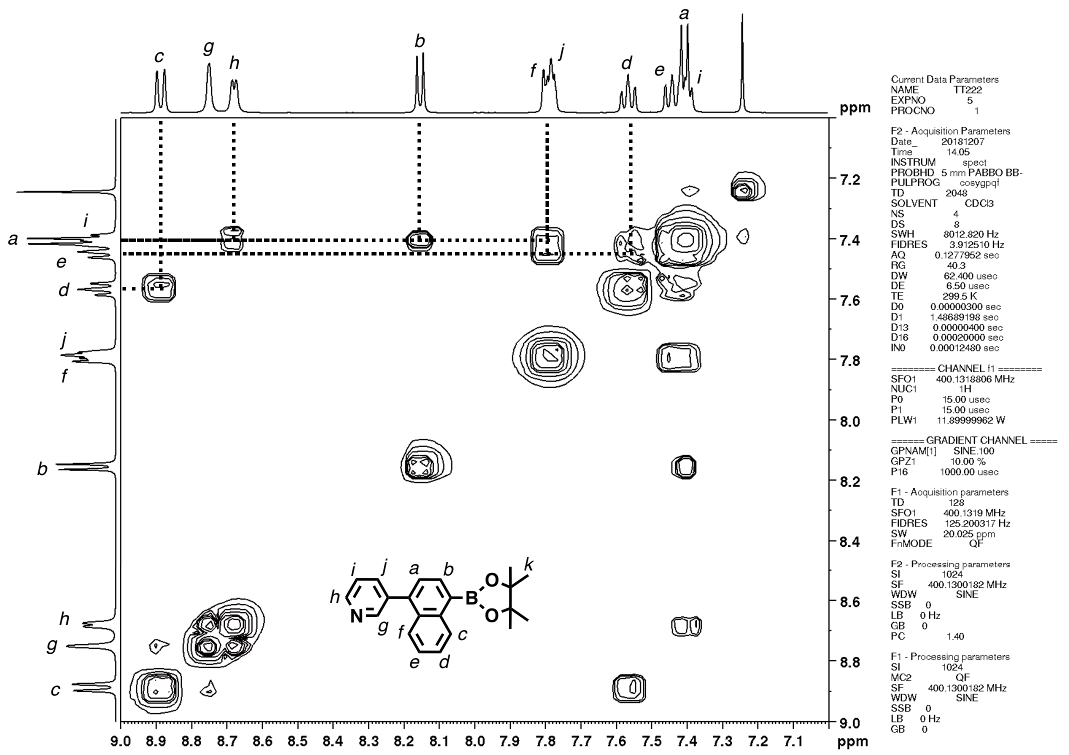

Figure S8. ^{1}H-^{1}H COSY spectrum (400 MHz, $CDCl_3$, r.t.) of 4-(3-pyridyl)naphthyl boronate.

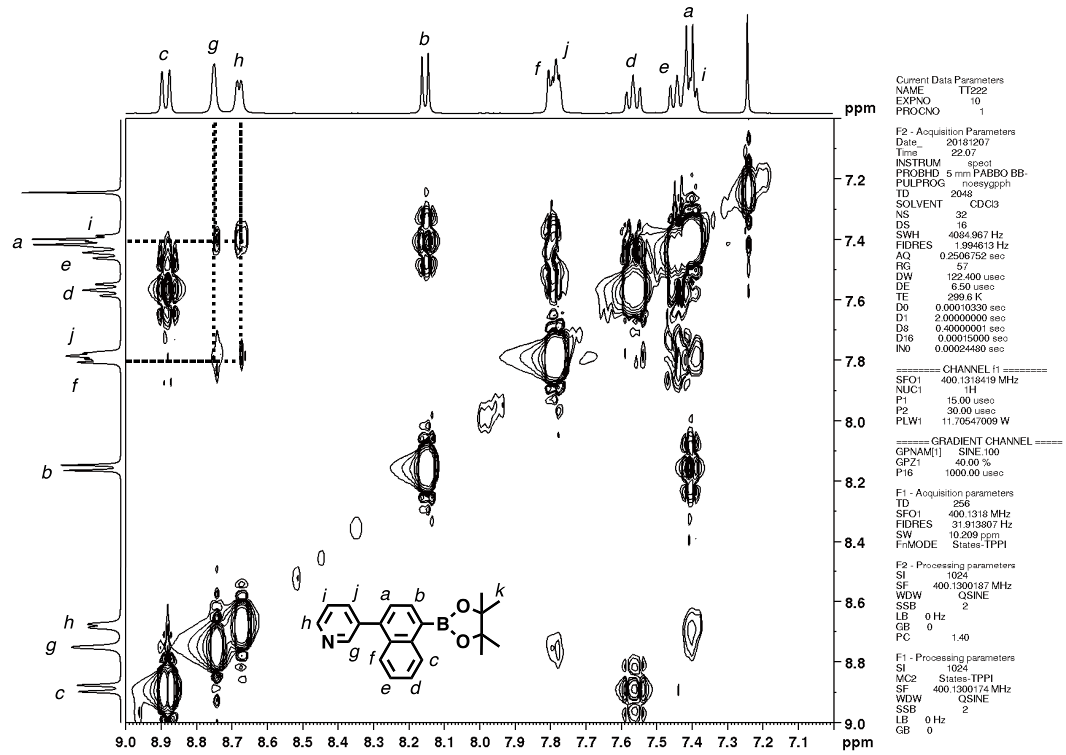

Figure S9. NOESY NMR spectrum (400 MHz, $CDCl_3$, r.t.) of 4-(3-pyridyl)naphthyl boronate.

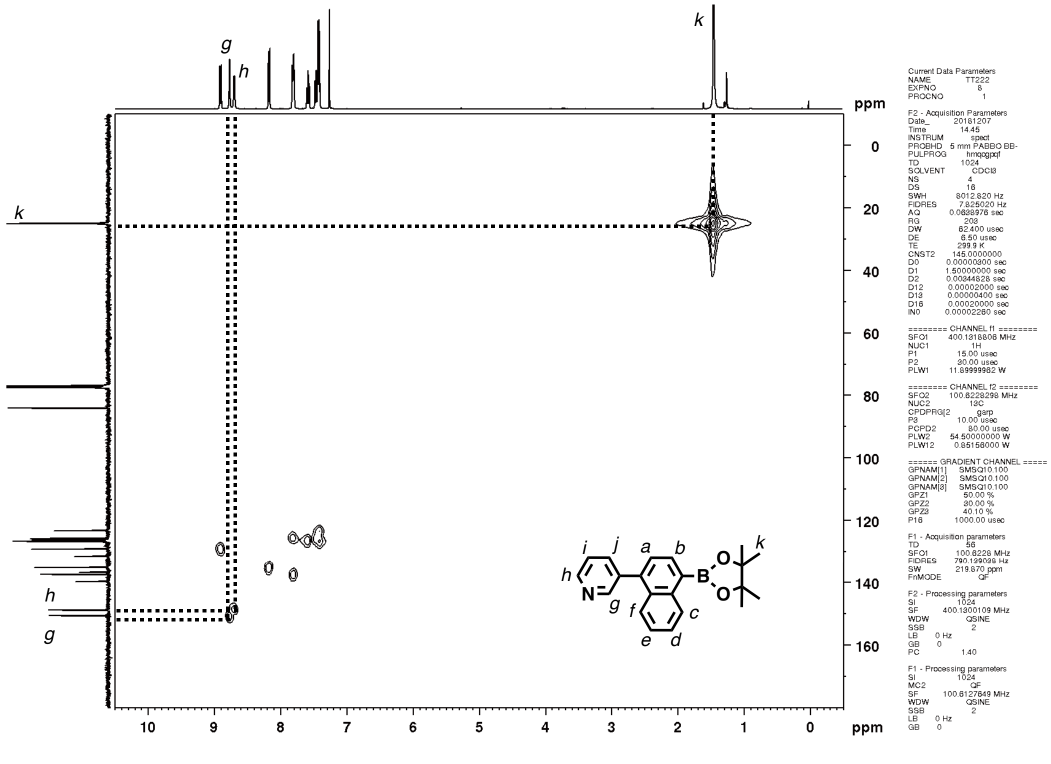

Figure S10a. HSQC spectrum (400 MHz, $CDCl_3$, r.t.) of 4-(3-pyridyl)naphthyl boronate.

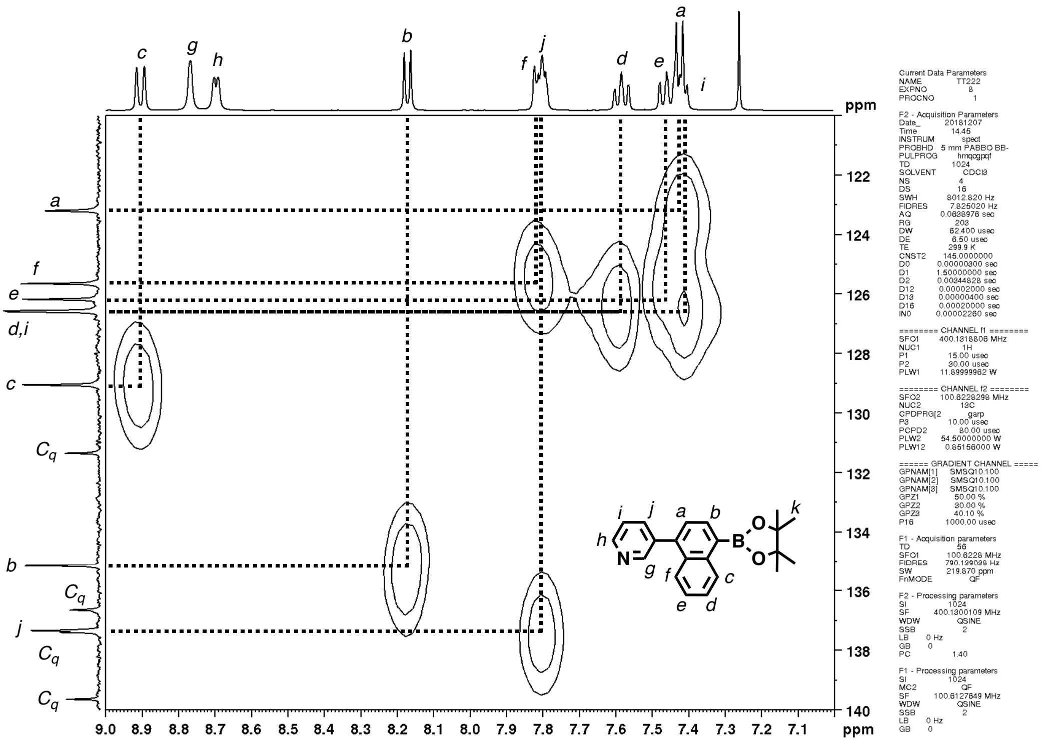

Figure S10b. HSQC spectrum (400 MHz, $CDCl_3$, r.t.) of 4-(3-pyridyl)naphthyl boronate.

Analysis Info

Analysis Name	D:\Data\akita\17Tsutsui\TT222\4-(3-pyridyl)naphthylbronic acid_MeOH.d	Acquisition Date	2019/01/16 19:57:27
Method	esi_posi_wide.m	Operator	BDAL@DE
Sample Name	4-(3-pyridyl)naphthylbronic acid_MeOH	Instrument	micrOTOF 213750.10321
Comment			

Acquisition Parameter

Source Type	ESI	Ion Polarity	Positive	Set Nebulizer	3.0 Bar
Focus	Not active			Set Dry Heater	180 • ‹C
Scan Begin	50 m/z	Set Capillary	4500 V	Set Dry Gas	4.0 l/min
Scan End	2000 m/z	Set End Plate Offset	-500 V	Set Divert Valve	Waste

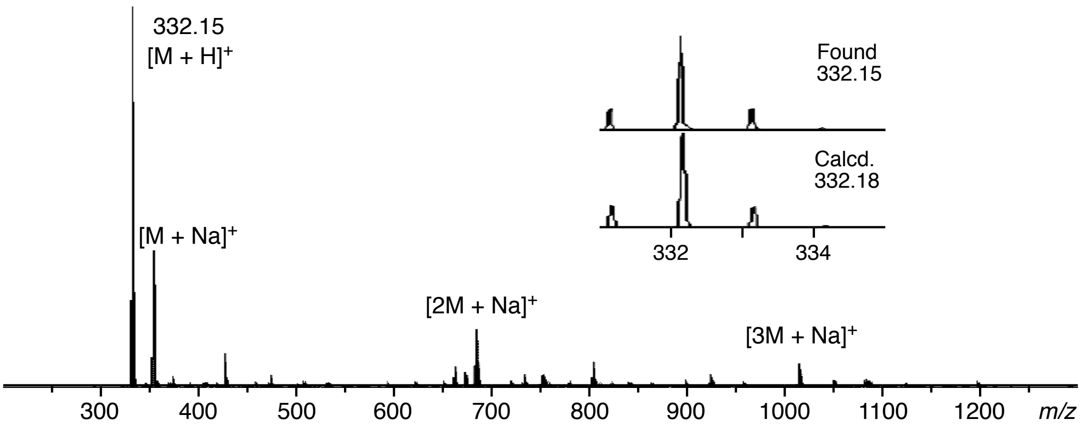

Figure S11. ESI-TOF MS spectrum (CH_3OH) of 4-(3-pyridyl)naphthyl boronate.

Synthesis of ligand 1 TT223, 263

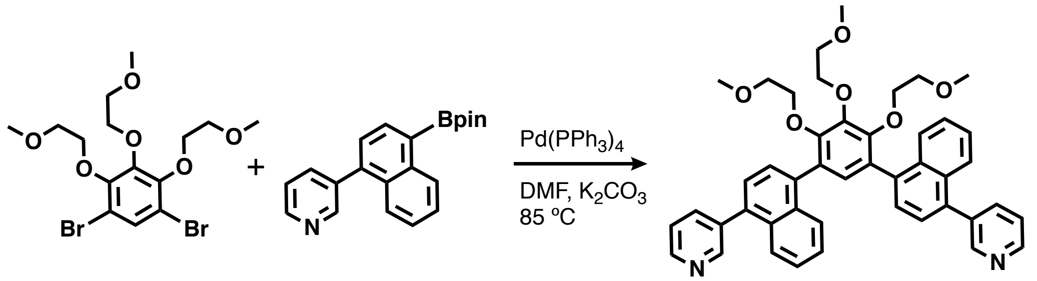

1,5-Dibromo-2,3,4-tri(2-methoxyethoxy)benzene[S1] (748 mg, 1.63 mmol), 4-(3-pyridyl)naphthyl boronate (1.62 g, 4.89 mmol), K_2CO_3 (972 mg, 7.03 mmol), $Pd(PPh_3)_4$ (551 mg, 0.478 mmol), and dry DMF (200 mL) were added to a 300 mL 2-necked glass flask filled with N_2. The resulted solution was stirred at 85 °C for 8 d. The mixture was concentrated under reduced pressure. After addition of water, the crude product was extracted with CH_2Cl_2 and then the combined organic phase was dried over Na_2SO_4, filtrated, and concentrated under reduced pressure. The crude product was purified by column chromatography (silica gel, ethyl acetate to CH_3OH) and GPC to afford **1** as a white solid (856 mg, 1.22 mmol; 74% yield).

^{1}H NMR (400 MHz, $CDCl_3$, r.t.): δ 2.98 (s, 6H), 3.26 (m, 4H), 3.47 (s, 3H), 3.87 (t, J = 4.6 Hz, 2H), 4.00 (m, 4H), 4.45 (m, 2H), 7.14 (s, 0.5 H), 7.15 (s, 0.5 H), 7.43-7.52 (m, 8H), 7.61 (d, J = 7.2 Hz, 1H), 7.62 (d, J = 7.2 Hz, 1H), 7.84-7.86 (m, 4H), 7.92 (d, J = 8.0 Hz, 1H), 7.97 (d, J = 8.4 Hz, 1H), 8.69 (d, J = 3.6 Hz, 2H), 8.79 (s, 2H). ^{13}C NMR (100 MHz, $CDCl_3$, r.t.): δ 58.6 (CH_3) × 2, 59.0 (CH_3), 71.6 (CH_2) × 2, 72.2 (CH_2), 73.0 (CH_2) × 3, 123.3 (CH) × 2, 125.6 (CH) × 2, 126.2 (CH) × 2, 126.3 (CH) × 2, 126.8 (CH) × 2, 127.0 (CH), 127.1 (CH), 127.5 (CH) × 2, 128.8 (CH), 129.0 (CH), 129.7 (C_q), 129.8 (C_q), 131.7 (C_q), 132.7 (C_q), 136.1 (C_q), 136.6 (C_q), 136.9 (C_q), 137.5 (CH) × 2, 145.7 (C_q), 145.8 (C_q), 148.7 (CH) × 2, 150.8 (CH) × 2, 151.2 (C_q), 151.3 (C_q). FT-IR (KBr, cm^{-1}): 3043, 2929, 2879, 2816, 1923, 1562, 1448, 1385, 1197, 1129, 1075, 846, 767, 720. HR MS (ESI, CH_3OH): m/z Calcd. for $C_{45}H_{42}N_2O_6Na$ $[M + Na]^+$ 729.2935, Found 729.2935.

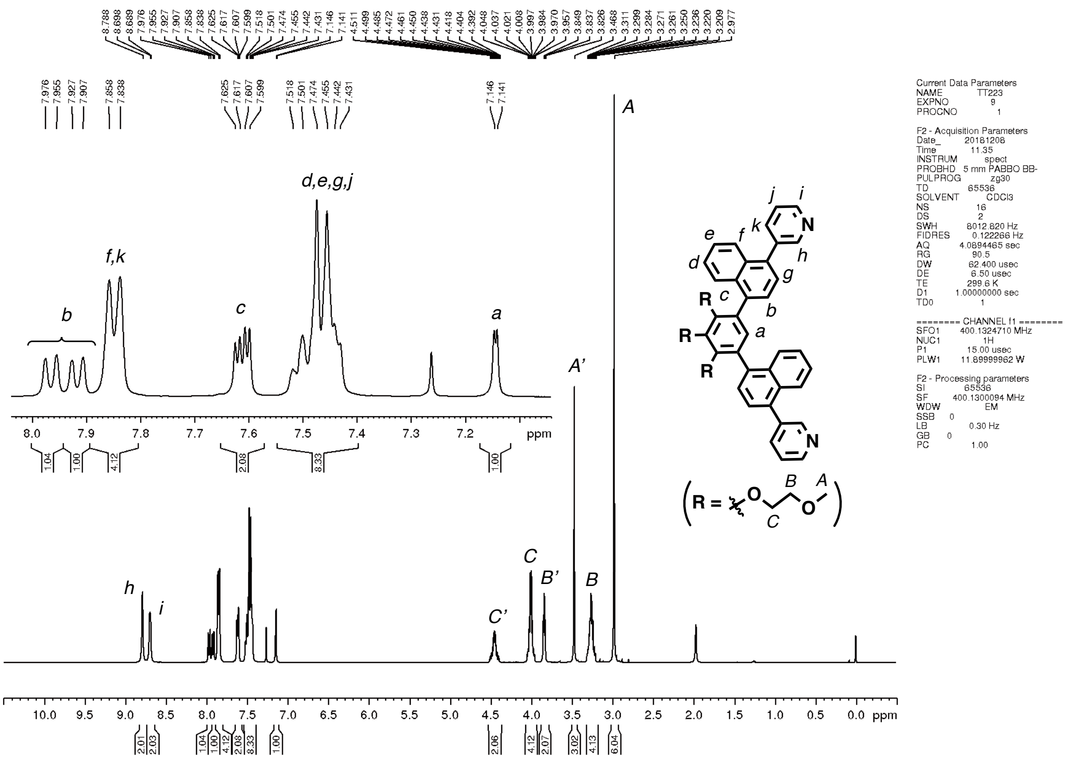

Figure S12. ^{1}H NMR spectrum (400 MHz, $CDCl_3$, r.t.) of **1**.

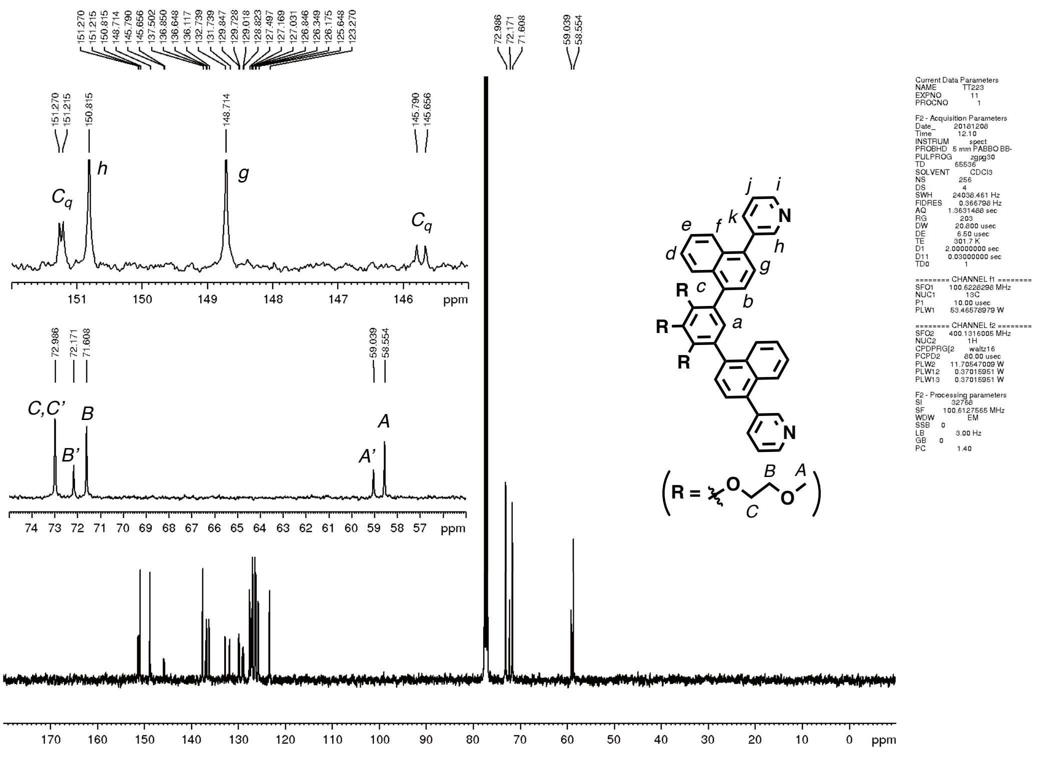

Figure S13a. ^{13}C NMR spectrum (400 MHz, $CDCl_3$, r.t.) of **1**.

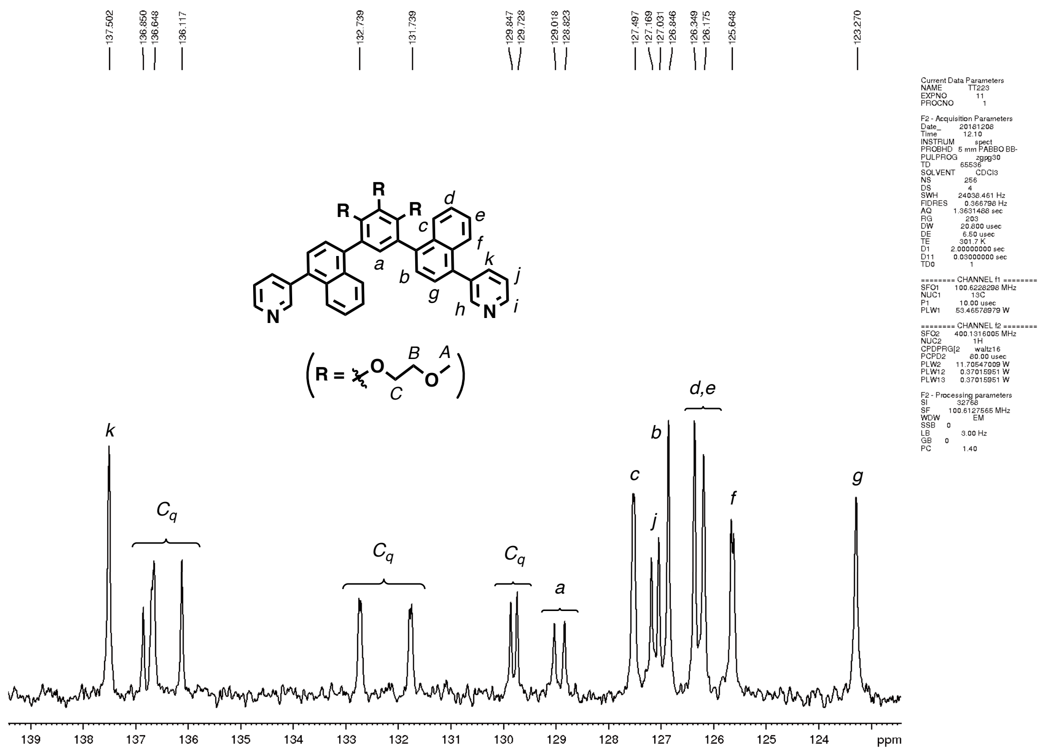

Figure S13b. ^{13}C NMR spectrum (400 MHz, $CDCl_3$, r.t.) of **1**.

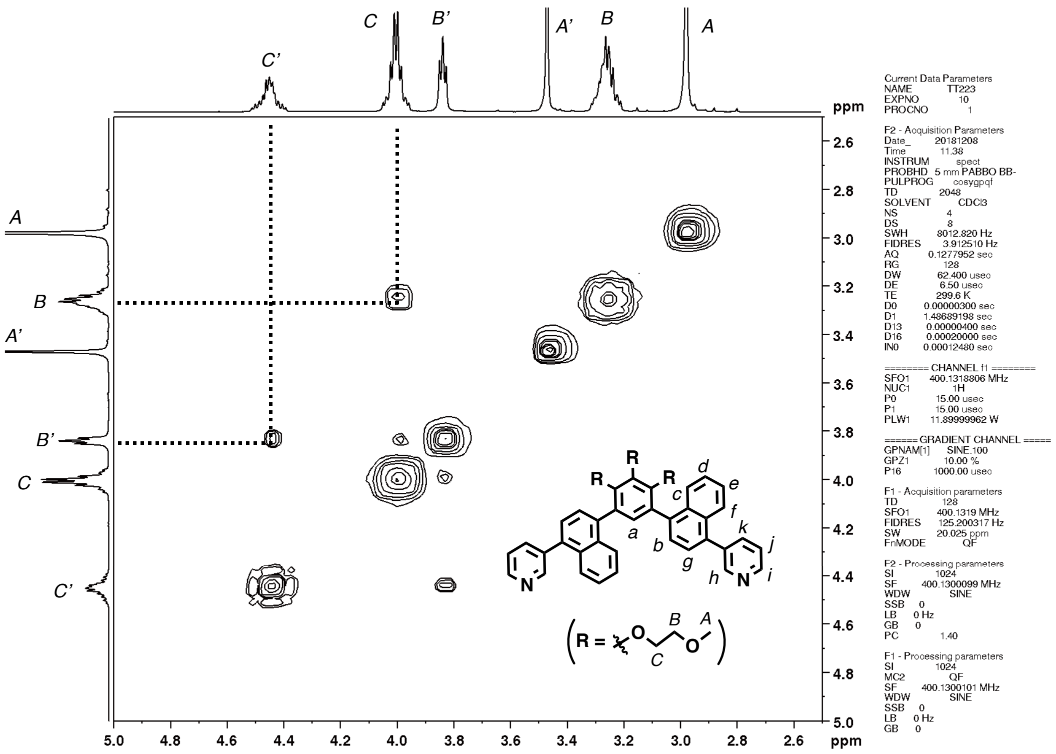

Figure S14a. ^1H-^1H COSY spectrum (400 MHz, $CDCl_3$, r.t.) of **1**.

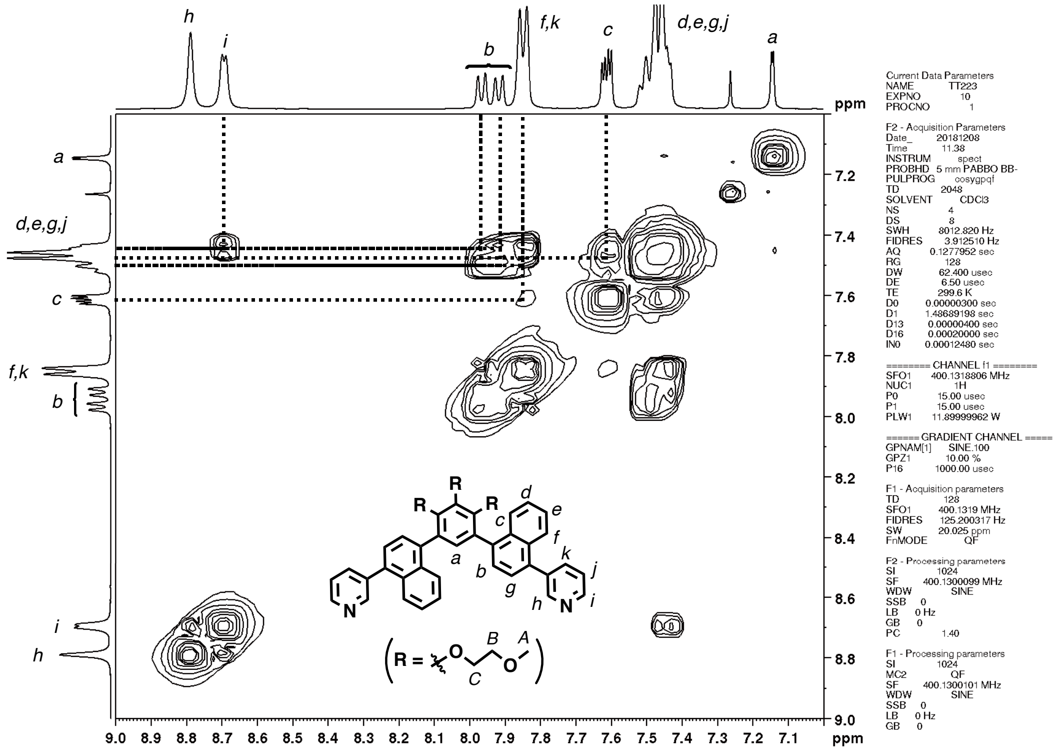

Figure S14b. ^{1}H-^{1}H COSY spectrum (400 MHz, $CDCl_3$, r.t.) of **1**.

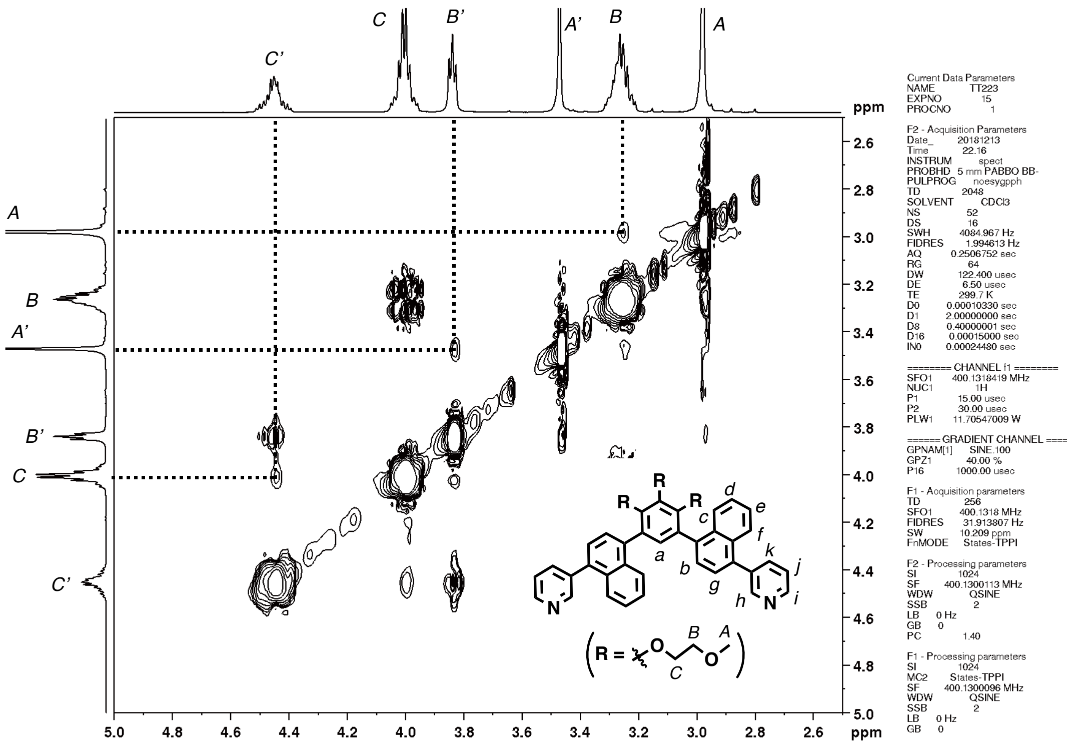

Figure S15a. NOESY NMR spectrum (400 MHz, $CDCl_3$, r.t.) of **1**.

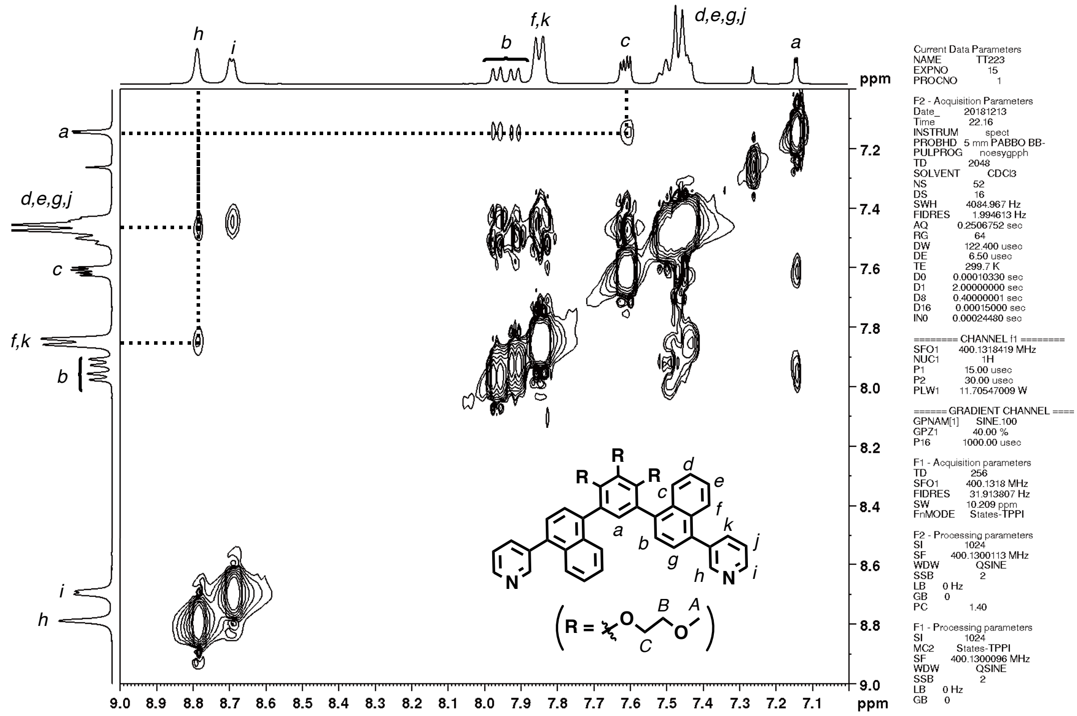

Figure S15b. NOESY NMR spectrum (400 MHz, $CDCl_3$, r.t.) of **1**.

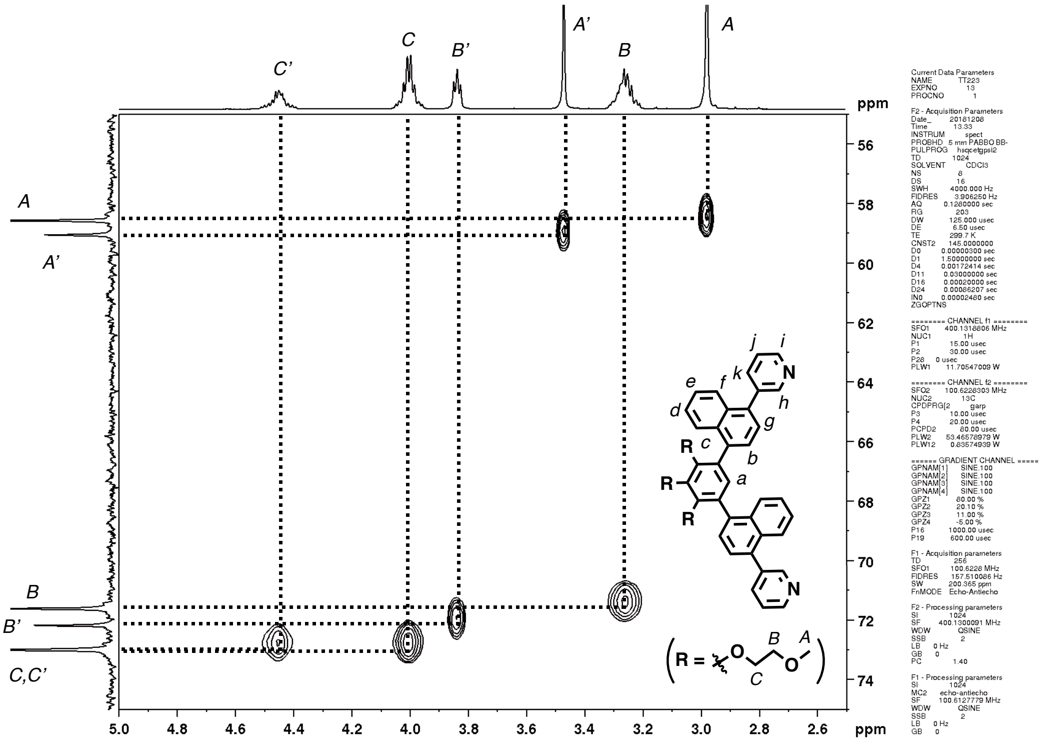

Figure S16a. HSQC NMR spectrum (400 MHz, $CDCl_3$, r.t.) of **1**.

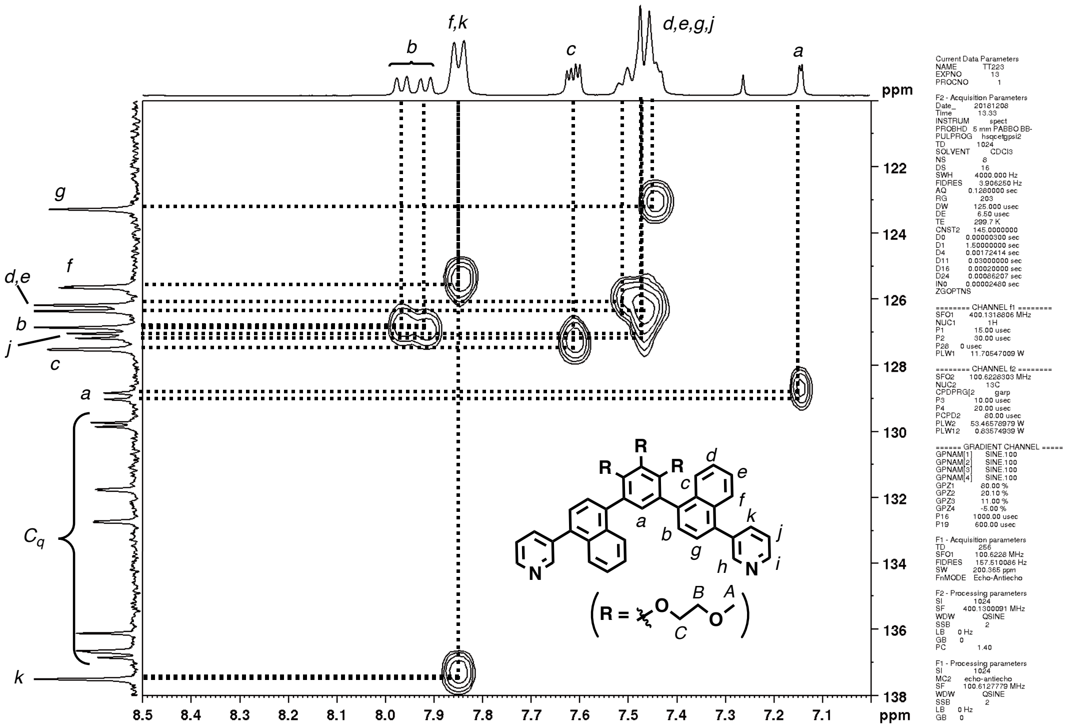

Figure S16b. HSQC NMR spectrum (400 MHz, $CDCl_3$, r.t.) of **1**.

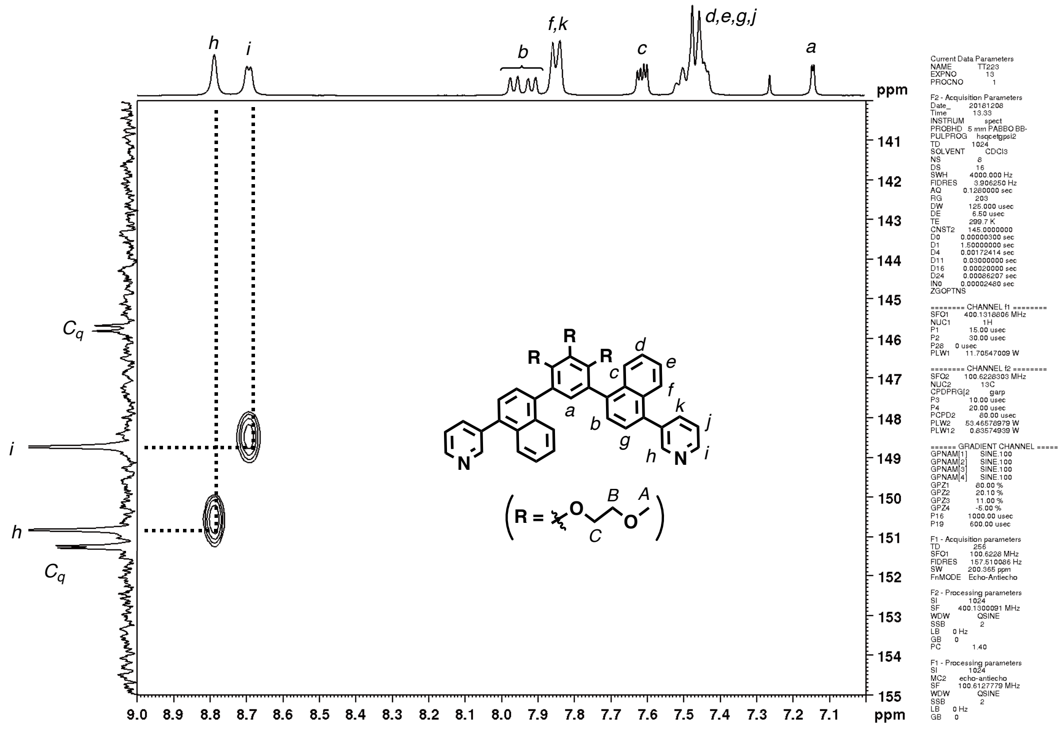

Figure S16c. HSQC NMR spectrum (400 MHz, $CDCl_3$, r.t.) of **1**.

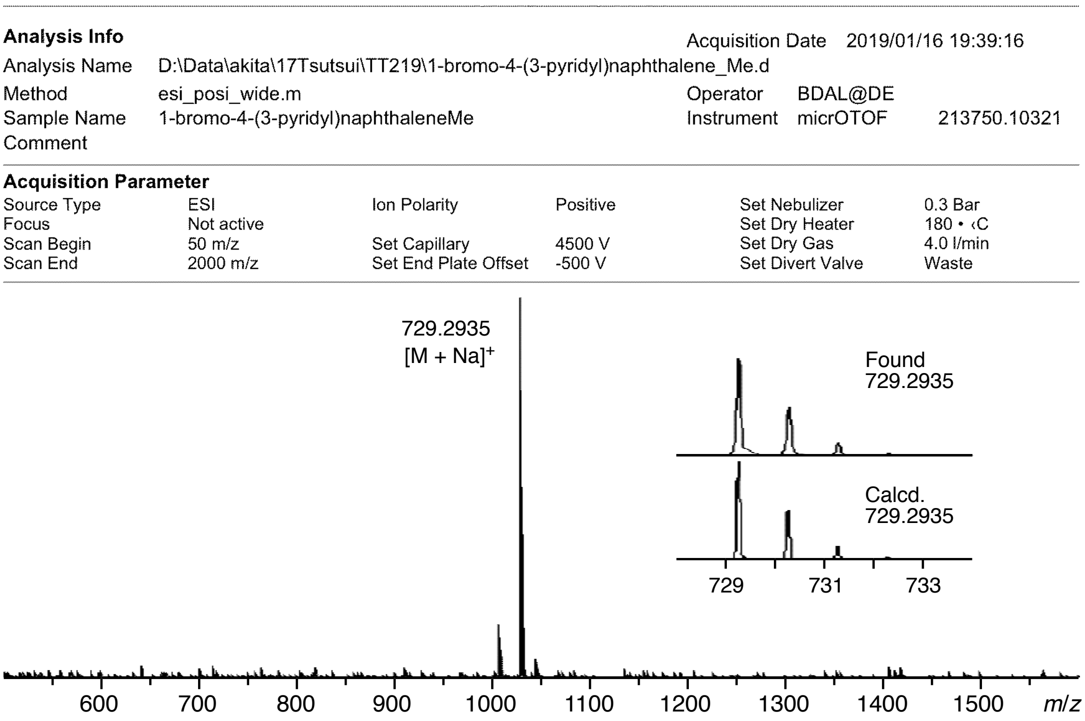

Figure S17. HR MS spectrum (ESI, CH_3OH) of **1**.

Formation of cage 2 TT236

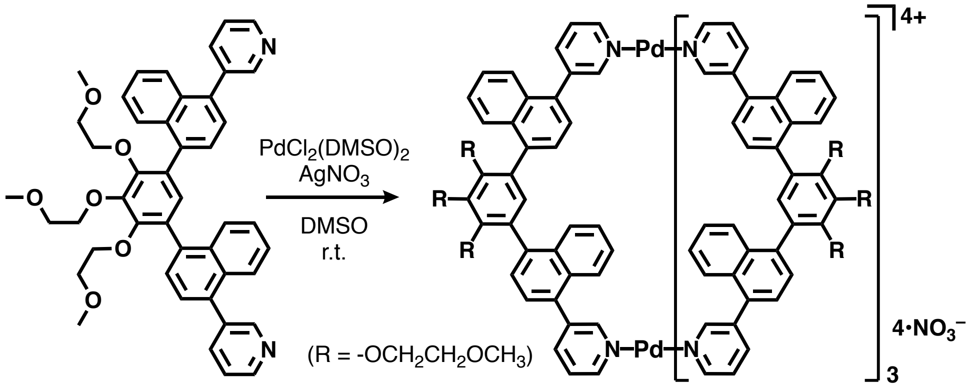

Ligand **1** (5.2 mg, 7.4 μmol), $PdCl_2(DMSO)_2$ (1.3 mg, 3.9 μmol), $AgNO_3$ (1.5 mg, 8.9 μmol), and DMSO-d_6 (0.5 mL) were added to a glass test tube and then the mixture was stirred at r.t. for 1 h. The quantitative formation of Pd(II)-linked cage **2** was confirmed by NMR and MS analyses. For the medium-scale synthesis of cage **2**, ligand **1** (52.3 mg, 74.4 μmol), $PdCl_2(DMSO)_2$ (12.6 mg, 37.7 μmol), $AgNO_3$ (12.9 mg, 75.5 μmol), and DMSO-d_6 (3.0 mL) were added to a glass test tube and then the mixture was stirred at r.t. for 2 h. After complete removal of the solvent under vacuum, the residue was treated with CH_2Cl_2 (5.0 mL) and shortly sonicated. The suspension was filtered using a syringe filter (200 nm pore size) and the filtrate was added dropwise into a centrifugation tube filled

with hexane (40 mL). The formed precipitate was collected via centrifugation, re-dissolved in CH_2Cl_2 (5.0 mL) and once more precipitated from hexane, followed by centrifugation and removal of the supernatant. The obtained solid was then dried under vacuum at r.t. in the dark to give cage **2** as a white solid in ~90% yield.

^{1}H NMR (400 MHz, DMSO-d_6, r.t.): δ 2.76 (m, 24 H), 3.07 (br, 16 H), 3.30 (br, 12 H), 3.67 (br, 8 H), 3.87 (br, 16 H), 4.23 (br, 8 H), 6.68-9.63 (m, 84 H). DOSY NMR (500 MHz, DMSO-d_6, 25 ºC): $D = 1.2 \times 10^{-10}$ m^2 s^{-1}. FT-IR (KBr, cm^{-1}): 2929, 1700, 1601, 1512, 1197, 1127, 1034, 964, 888, 844, 767, 707. ESI-TOF MS (DMSO): m/z 759.9 [**2** – 4•NO_3^-]$^{4+}$, 1033.9 [**2** – 3•NO_3^-]$^{3+}$, 1582.3 [**2** – 2•NO_3^-]$^{2+}$.

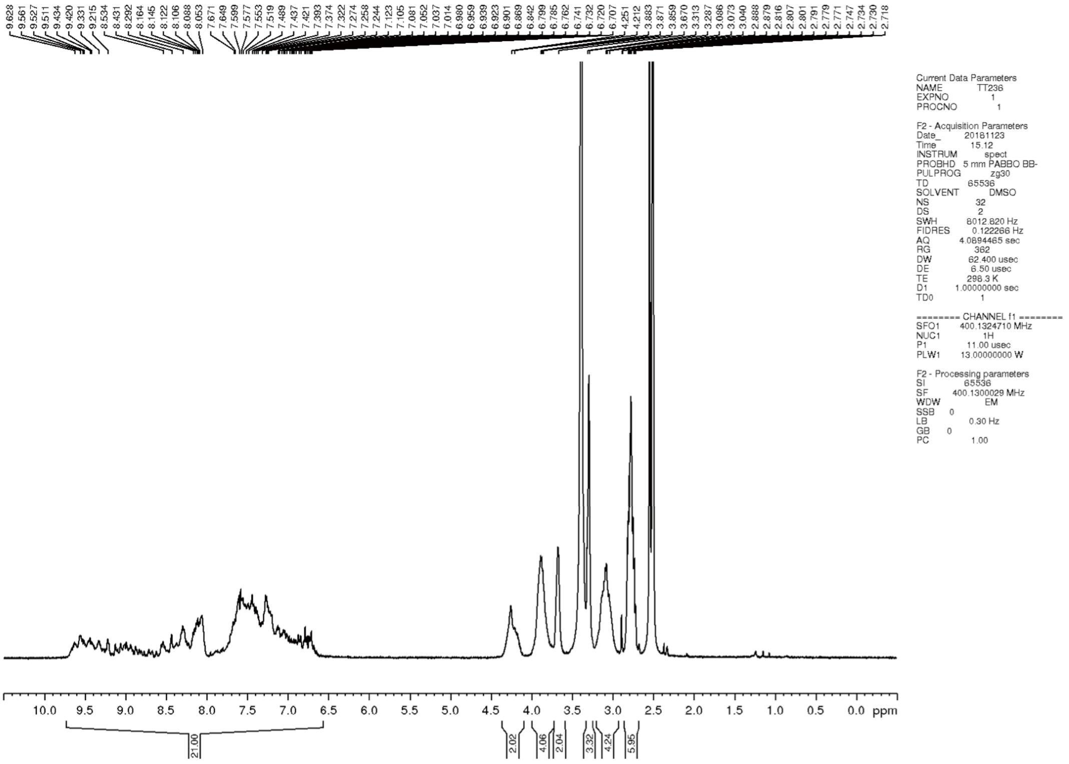

Figure S18. ^{1}H NMR spectrum (400 MHz, DMSO-d_6, r.t.) of **2**.

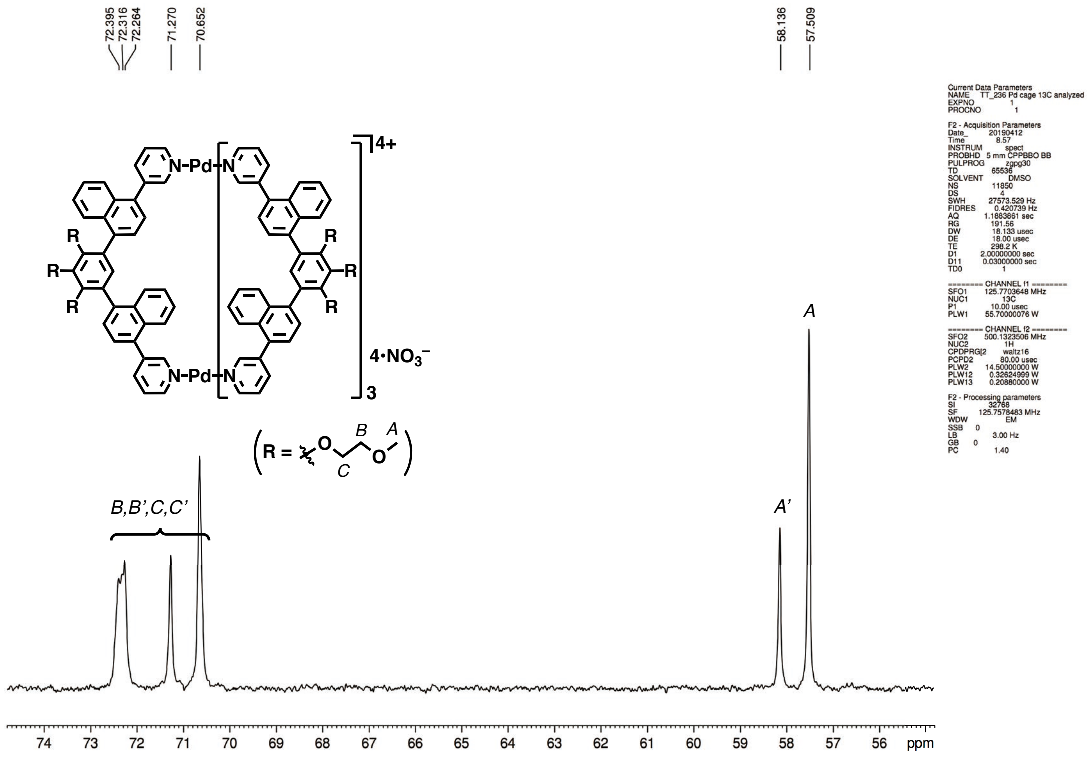

Figure S19a. ^{13}C NMR spectrum (500 MHz, DMSO-d_6, r.t.) of **2**.

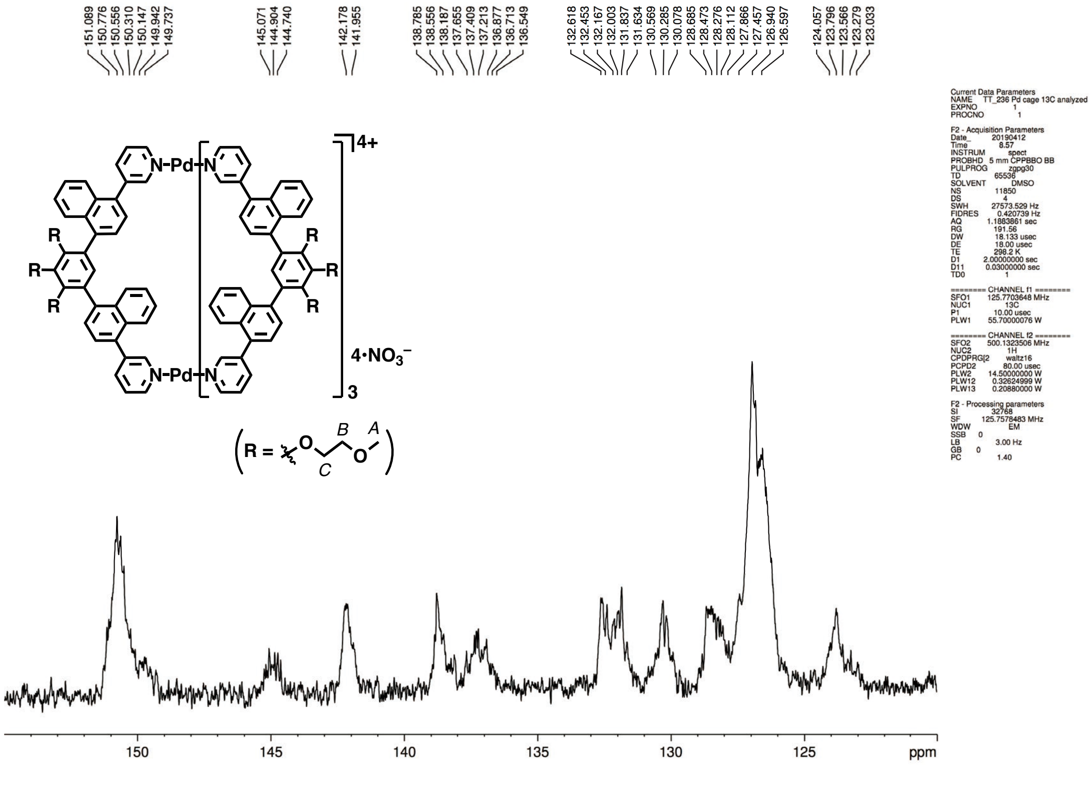

Figure S19b. ^{13}C NMR spectrum (500 MHz, DMSO-d_6, r.t.) of **2**.

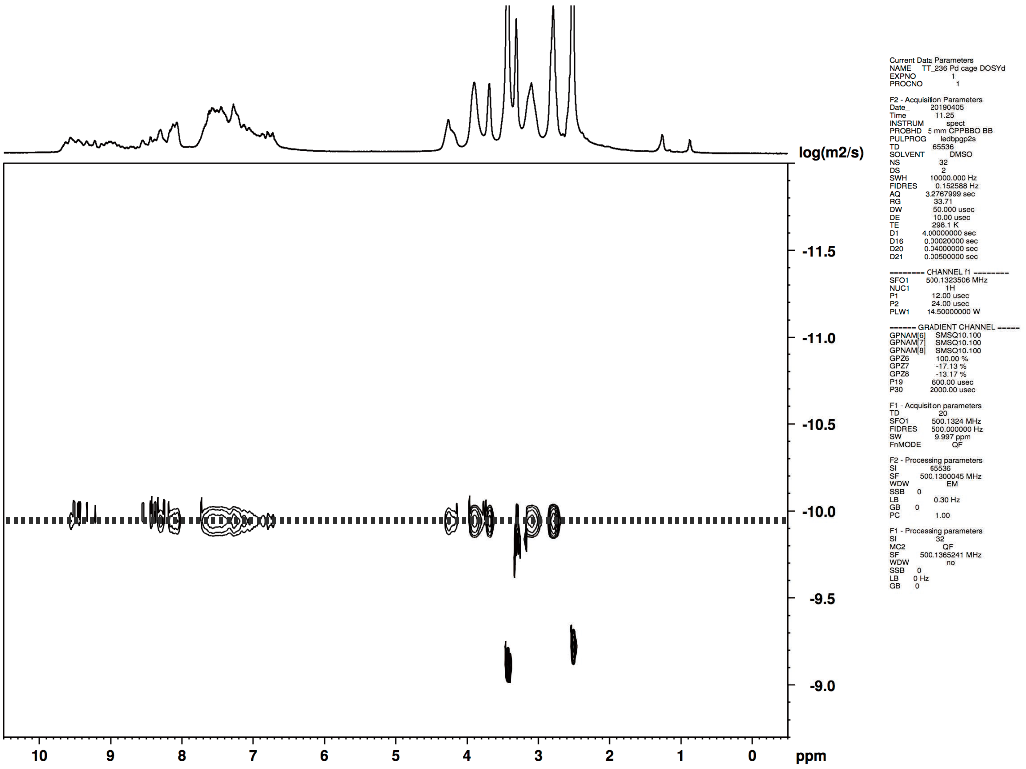

Figure S20. DOSY NMR spectrum (500 MHz, DMSO-d_6, 25 ºC) of **2**.

Analysis Info

Analysis Name	D:\Data\akita\17Tsutsui\TT236\Pdnaphthalenecage_purificated_DMSO.d	Acquisition Date	2018/12/06 18:52:30
Method	Pd Kusaba01.m	Operator	BDAL@DE
Sample Name	Pdnaphthalenecage_purificated_DMSO	Instrument	micrOTOF 213750.10321
Comment			

Acquisition Parameter

Source Type	ESI	Ion Polarity	Positive	Set Nebulizer	3.0 Bar
Focus	Not active			Set Dry Heater	30 • ‹C
Scan Begin	50 m/z	Set Capillary	4500 V	Set Dry Gas	6.0 l/min
Scan End	3000 m/z	Set End Plate Offset	-500 V	Set Divert Valve	Waste

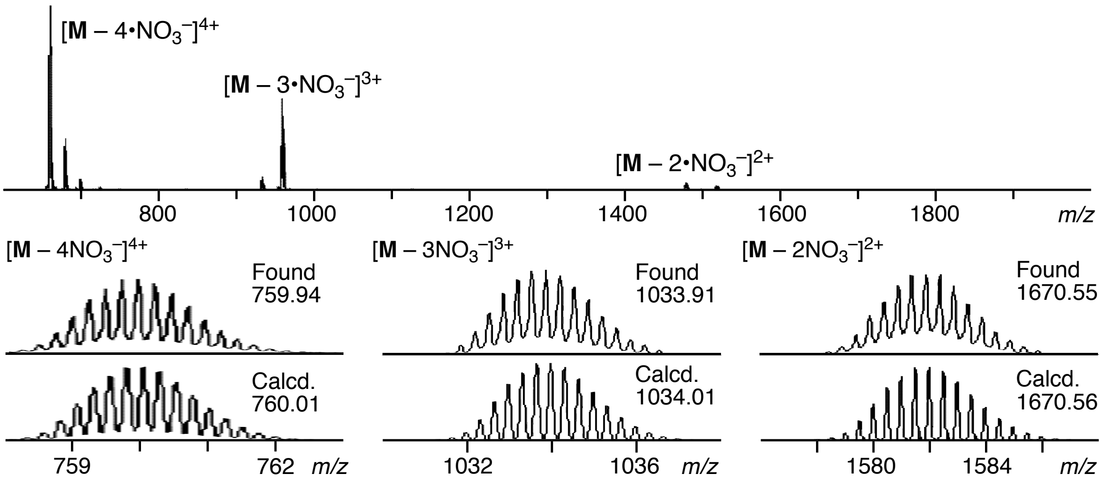

Figure S21. ESI-TOF MS spectrum (DMSO) of **2**.

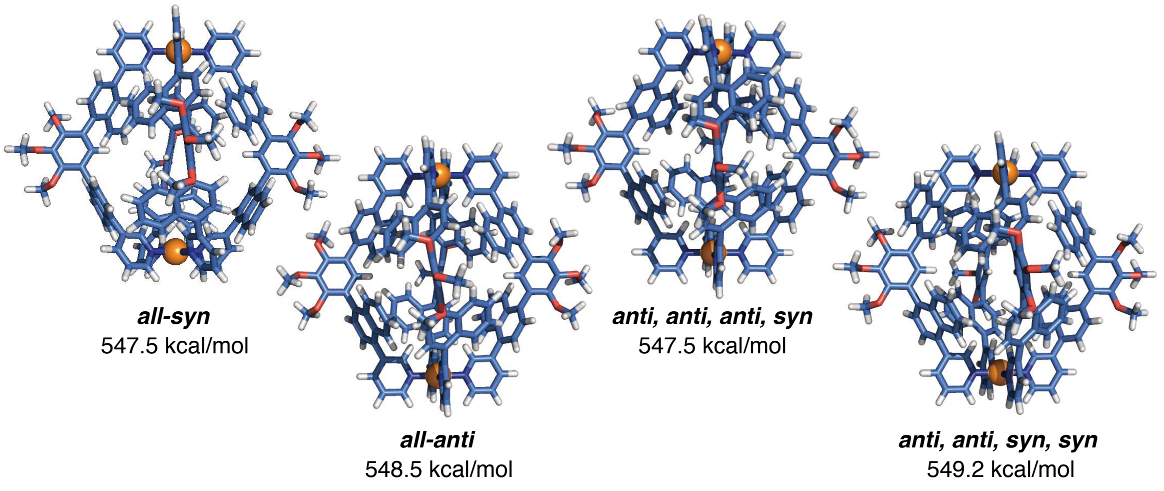

Figure S22. Optimized structures of **2** (four isomers; R = $-OCH_3$) and their energies.

Formation of cage 2'   TT232

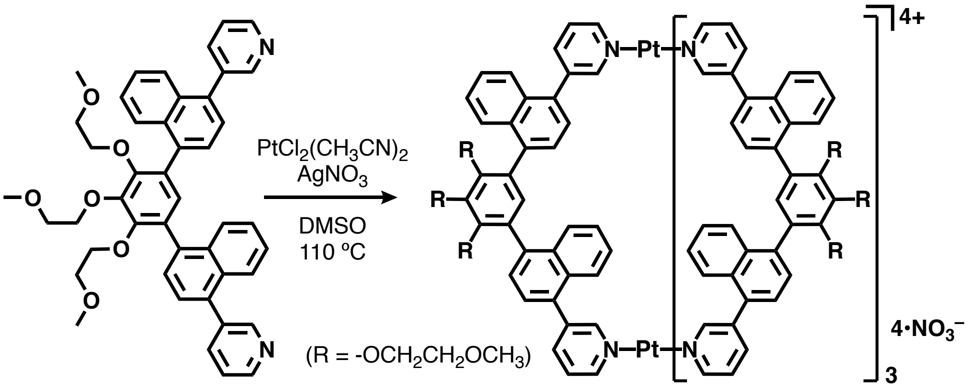

Ligand **1** (5.2 mg, 7.4 μmol), $PtCl_2(CH_3CN)_2$ (1.3 mg, 3.9 μmol), $AgNO_3$ (1.4 mg, 8.0 μmol), and DMSO-d_6 (0.5 mL) were added to a glass test tube and then the mixture was stirred at 110 °C for 1 h. The quantitative formation of Pt(II)-linked cage **2'** was confirmed by NMR and MS analyses. For the medium-scale synthesis of cage **2'**, ligand **1** (52.6 mg, 74.4 μmol), $PtCl_2(CH_3CN)_2$ (13.0 mg, 37.3 μmol), $AgNO_3$ (12.8 mg, 75.3 μmol), and DMSO-d_6 (3.0 mL) were added to a glass test tube and then the mixture was stirred at 110 °C for 2 h. After complete removal of the solvent under vacuum, the residue was treated with CH_2Cl_2 (3.0 mL) and shortly sonicated. The suspension was filtered using a syringe filter (200 nm pore size) and the filtrate was added dropwise into a centrifugation tube filled with hexane (40 mL). The formed precipitate was collected via centrifugation, re-dissolved in CH_2Cl_2 (1.0 mL) and once more precipitated from hexane, followed by centrifugation and removal of the supernatant. The obtained solid was then dried under

vacuum at r.t. in the dark to give cage **2’** as a beige solid in ~80% yield.

^{1}H NMR (500 MHz, DMSO-d_6, r.t.): δ 2.78 (m, 24 H), 3.06 (br, 16 H), 3.30 (br, 12 H), 3.67 (br, 8 H), 3.89 (br, 16 H), 4.23 (br, 8 H), 6.40-9.59 (m, 84 H). DOSY NMR (500 MHz, DMSO-d_6, 25 °C): $D = 1.0 \times 10^{-10}$ m^2 s^{-1}. FT-IR (KBr, cm^{-1}): 2929, 2817, 2427, 1512, 1239, 1197, 1127, 1074, 1058, 1032, 766, 709. ESI-TOF MS (DMSO): m/z 804.3 [**2’** – 4•NO_3^-]$^{4+}$, 1093.5 [**2’** – 3•NO_3^-]$^{3+}$, 1670.6 [**2’** – 2•NO_3^-]$^{2+}$.

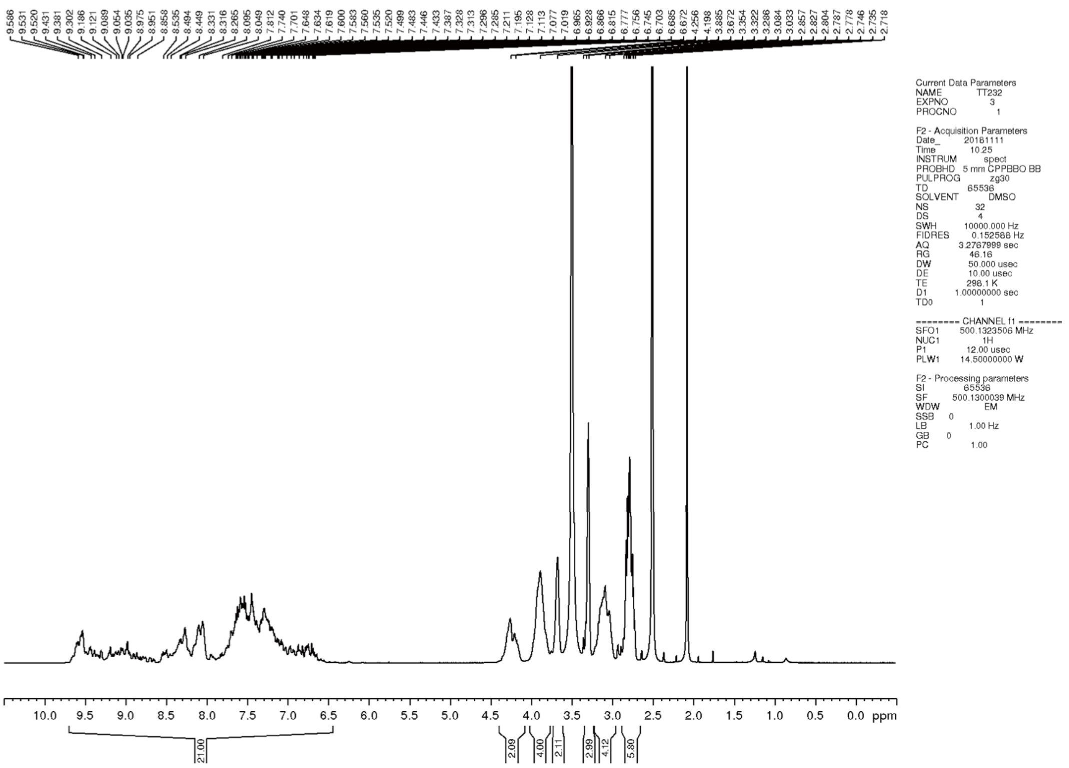

Figure S23. ^{1}H NMR spectrum (500 MHz, DMSO-d_6, r.t.) of **2’**.

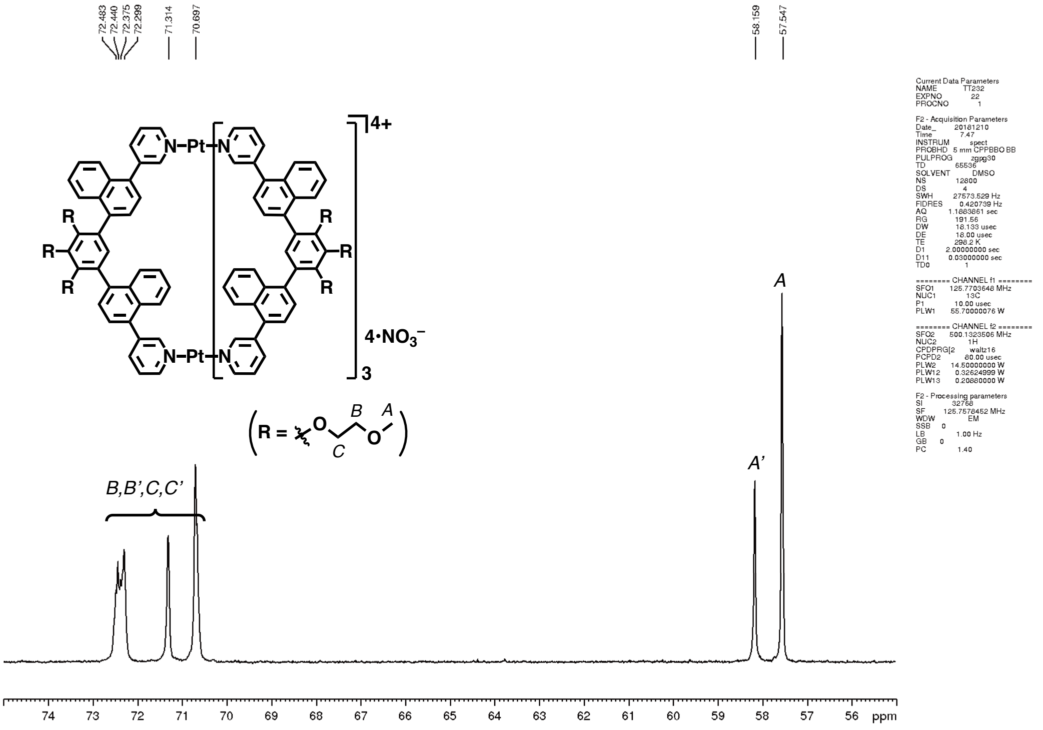

Figure S24a. ^{13}C NMR spectrum (500 MHz, DMSO-d_6, r.t.) of **2'**.

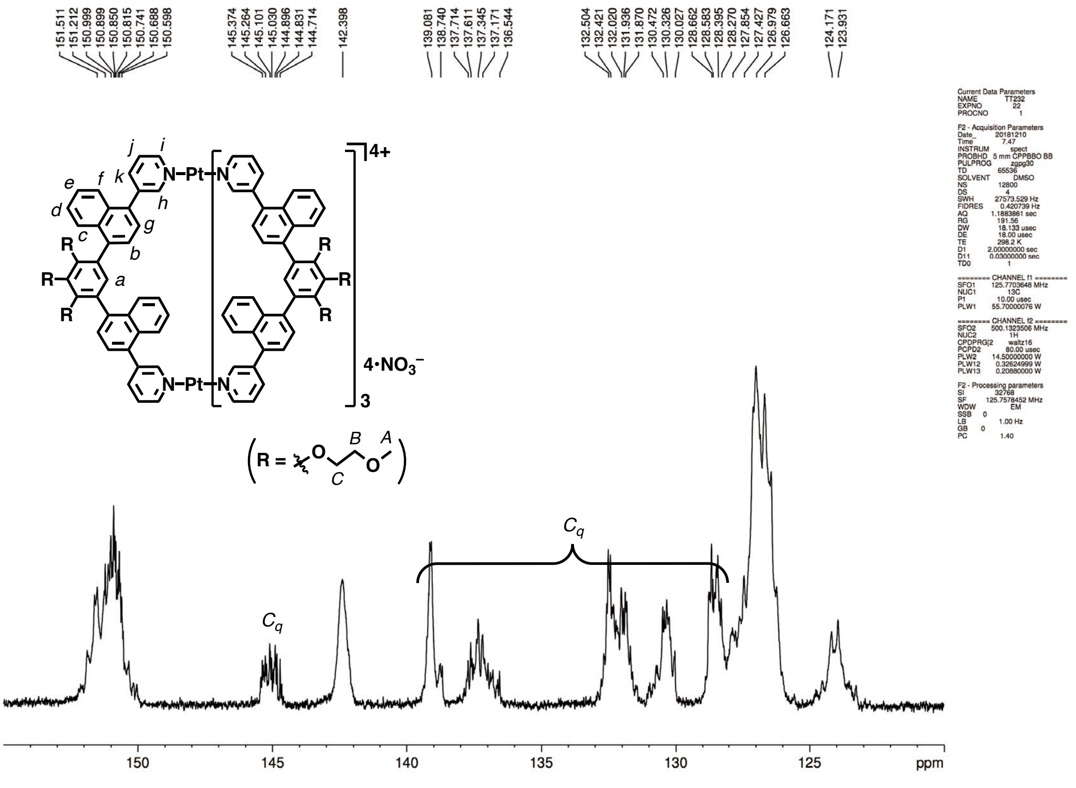

Figure S24b. ^{13}C NMR spectrum (500 MHz, DMSO-d_6, r.t.) of **2'**.

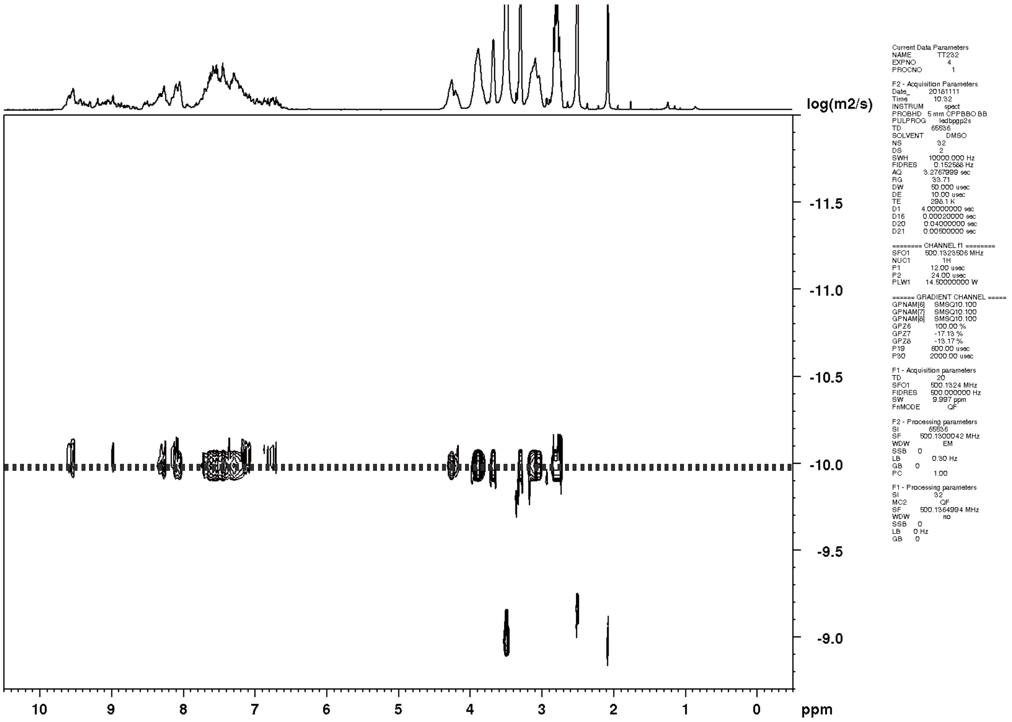

Figure S25. DOSY NMR spectrum (500 MHz, DMSO-d_6, 25 ºC) of **2’**.

Analysis Info | Acquisition Date 2018/11/11 15:13:45

Analysis Name	D:\Data\akita\17Tsutsui\TT232\TT232_Ptnapthalenecage_DMSO.d		
Method	Pd Kusaba01.m	Operator	BDAL@DE
Sample Name	TT232_Ptnapthalenecage_DMSO	Instrument	micrOTOF 213750.10321
Comment			

Acquisition Parameter

Source Type	ESI	Ion Polarity	Positive	Set Nebulizer	3.0 Bar
Focus	Not active			Set Dry Heater	30 • ‹C
Scan Begin	50 m/z	Set Capillary	4500 V	Set Dry Gas	6.0 l/min
Scan End	3000 m/z	Set End Plate Offset	-500 V	Set Divert Valve	Waste

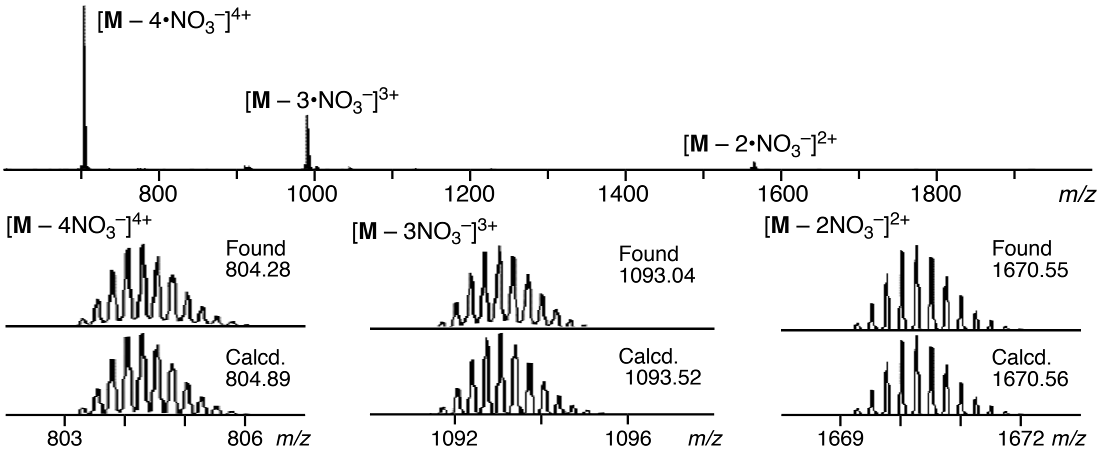

Figure S26. ESI-TOF MS spectrum (DMSO) of **2’**.

Preparation of 2•(Tp)$_2$ TT251

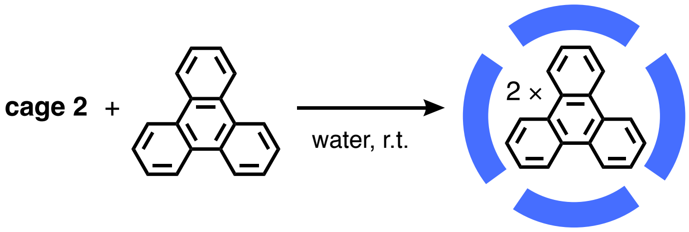

Pd(II)-linked cage **2** (1.0 mg, 0.3 μmol), triphenylene (**Tp**; excess), and D_2O (0.5 mL) were added to a glass test tube. The mixture was stirred at r.t. for 1 h. After filtration of the resultant solution, the formation of host-guest complex **2•(Tp)**$_2$ was confirmed by NMR and ESI-TOF MS analyses. The 1:2 host-guest ratio was precisely estimated by the ^{1}H NMR integral ratios of **2•(Tp)**$_2$ after disassembly in DMSO-d_6 (at 80 °C for 1 h under high dilution conditions).

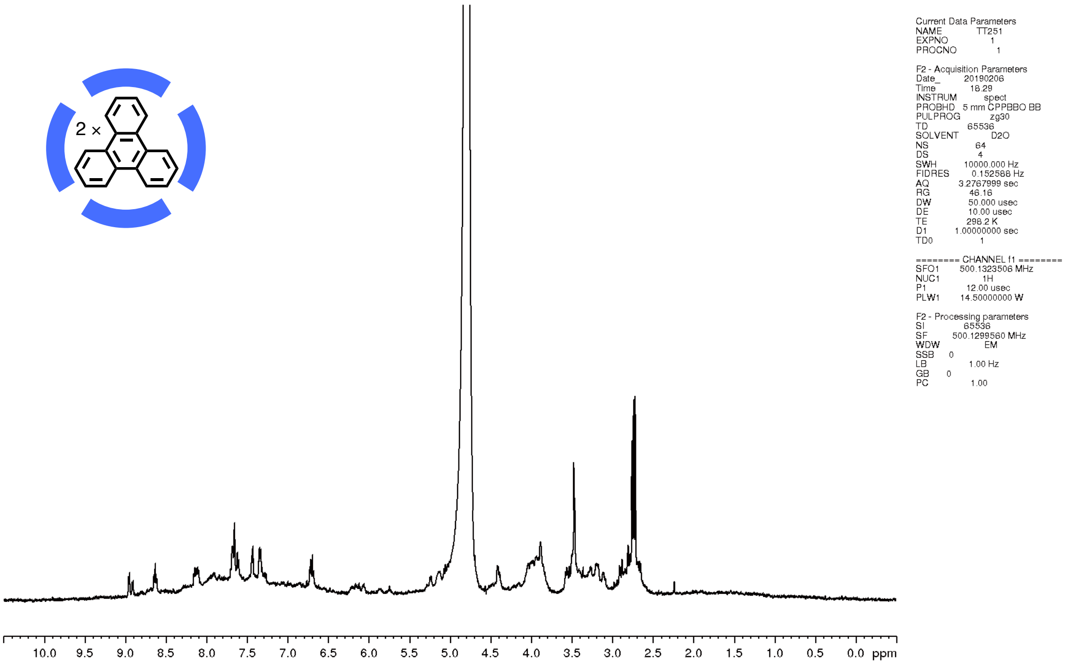

Figure S27a. ^{1}H NMR spectrum (500 MHz, D_2O, r.t.) of **2•(Tp)**$_2$.

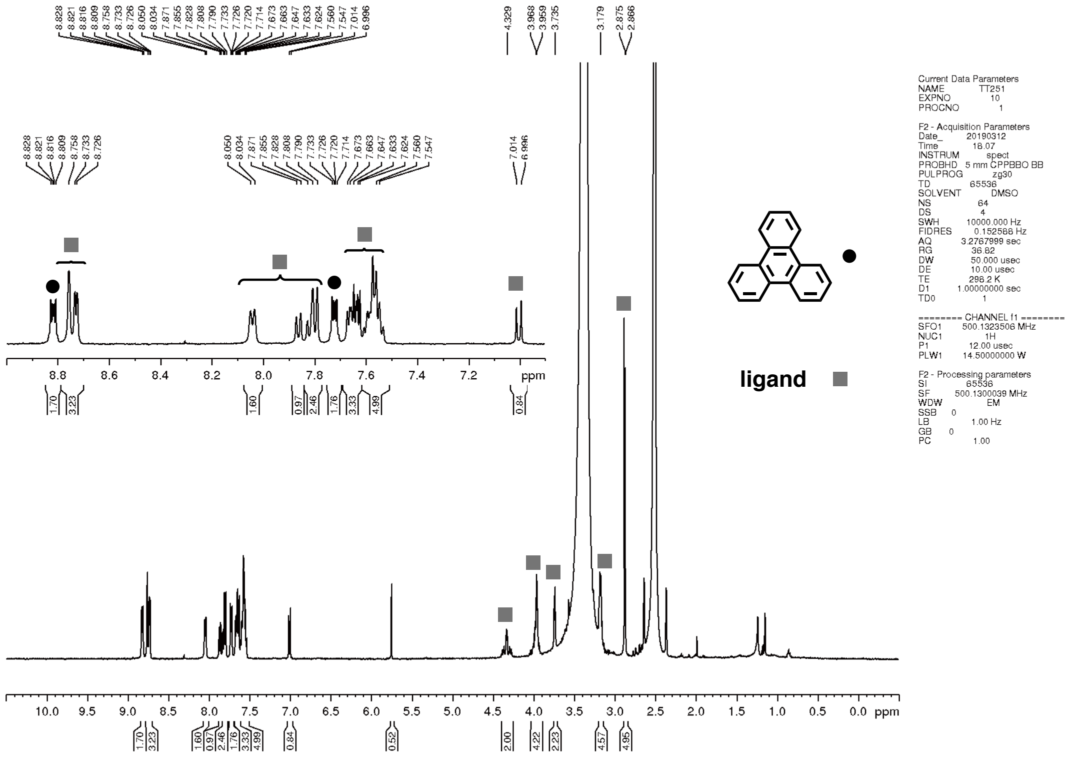

Figure S27b. ^{1}H NMR spectrum (500 MHz, DMSO-d_6, r.t.) of **2•(Tp)**$_2$ after disassembly.

**Analysis Info**		Acquisition Date	2019/03/06 15:54:08
Analysis Name	D:\Data\akita\17Tsutsui\TT251\Pdcage(Tp)2-H2O-sonomama.d		
Method	Pd Kusaba01.m	Operator	BDAL@DE
Sample Name	Pdcage(Tp)2-H2O-sonomama	Instrument	micrOTOF 213750.10321
Comment			

Acquisition Parameter

Source Type	ESI	Ion Polarity	Positive	Set Nebulizer	3.0 Bar
Focus	Not active			Set Dry Heater	30 • ‹C
Scan Begin	50 m/z	Set Capillary	4500 V	Set Dry Gas	6.0 l/min
Scan End	3000 m/z	Set End Plate Offset	-500 V	Set Divert Valve	Waste

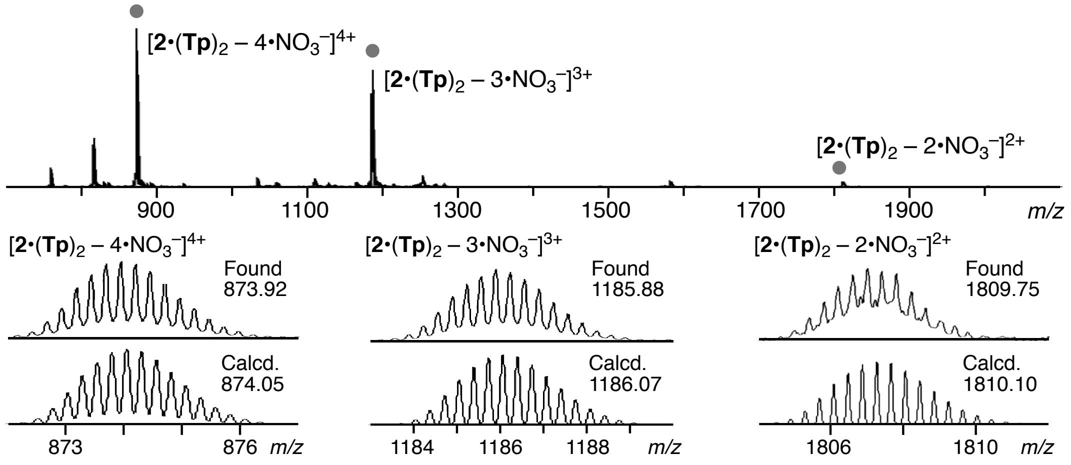

Figure S27c. ESI-TOF MS spectrum (H_2O) of **2•(Tp)**$_2$.

Preparation of 2·Ad TT251

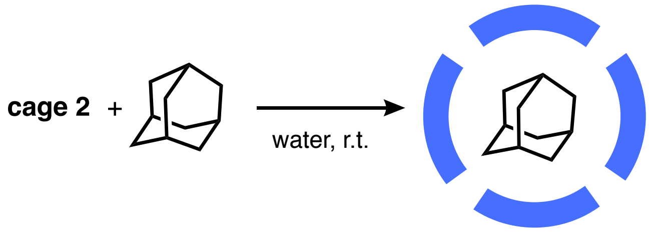

Pd(II)-linked cage **2** (1.0 mg, 0.3 μmol), adamantane (**Ad**; excess), and D_2O (0.5 mL) were added to a glass test tube. The mixture was stirred at r.t. for 1 h. After filtration of the resultant solution, the formation of host-guest complex **2•Ad** was confirmed by NMR analysis. The 1:1 host-guest ratio was precisely estimated by the ^1H NMR integral ratios of **2•Ad** after disassembly in DMSO-d_6 (at 80 °C for 1 h under high dilution conditions).

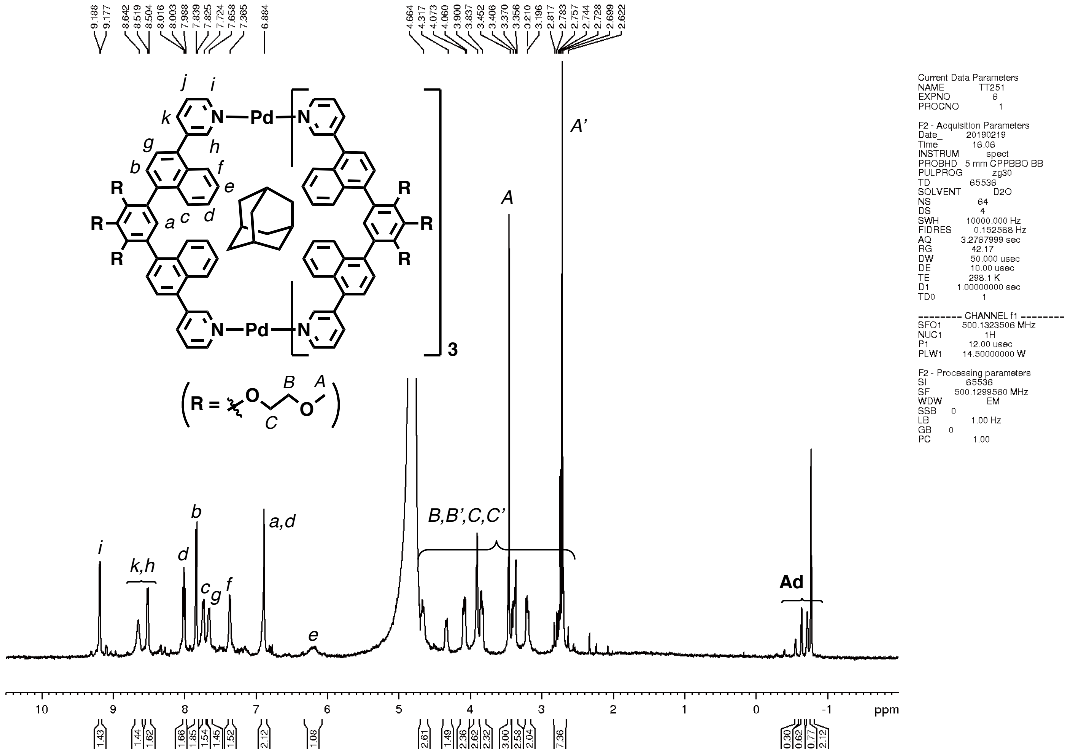

Figure S28a. ^1H NMR spectrum (500 MHz, D_2O, r.t.) of **2•Ad**.

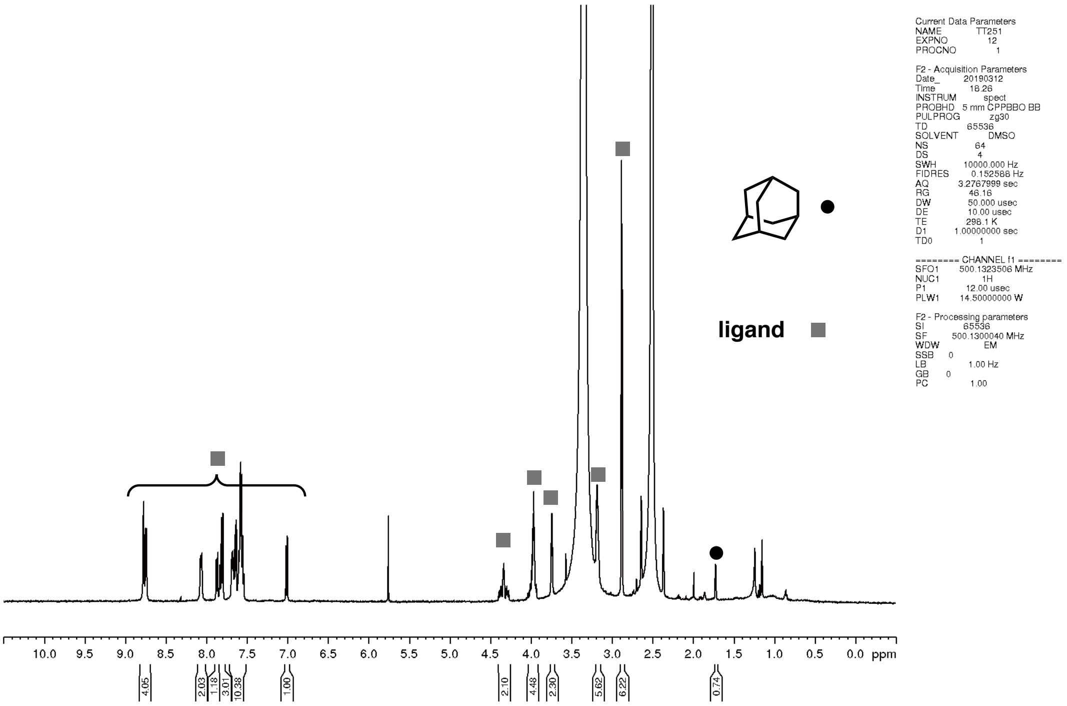

Figure S28b. ^{1}H NMR spectrum (500 MHz, DMSO-d_6, r.t.) of **2•Ad** after disassembly.

Preparation of 2•(Cor)$_2$ and 2'•(Cor)$_2$

TT251

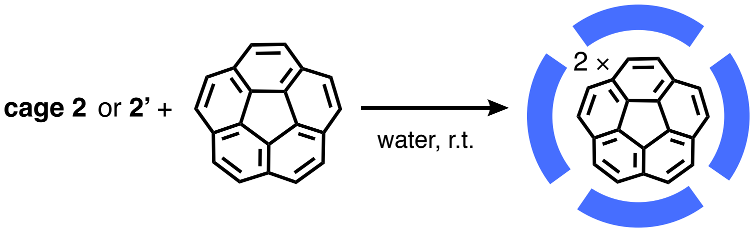

Pd(II)-linked cage **2** (1.0 mg, 0.3 μmol), corannulene (**Cor**; excess), and D_2O (0.5 mL) were added to a glass test tube. The mixture was stirred at r.t. for 1 h. After filtration of the resultant solution, the formation of host-guest complex **2•**(**Cor**)$_2$ was confirmed by NMR and ESI-TOF MS analyses. The 1:2 host-guest ratio was precisely estimated by the ^{1}H NMR integral ratios of **2•**(**Cor**)$_2$ after disassembly in DMSO-d_6 (at 80 ºC for 1 h under high dilution conditions). Similarly, a mixture of Pt(II)-linked cage **2'** (1.0 mg, 0.3 μmol), **Cor** (excess), and D_2O (0.5 mL) was stirred at r.t. for 7 h (or at 80 ºC for 1 h). The formation of **2'•**(**Cor**)$_2$ was confirmed by NMR and ESI-TOF MS analyses.

2•(**Cor**)$_2$: ^{1}H NMR (500 MHz, D_2O, r.t.): δ 2.89 (s, 6H), 3.36-3.43 (br, 4H), 3.59 (s, 3H), 4.03 (br, 2H), 4.25 (br, 4H), 4.63 (br, 2H), 5.89 (s, 2H), 6.22 (m, 2H), 6.97 (m, 2H), 7.23 (s, 0.5H), 7.24 (s, 0.5H), 7.54 (d, J = 7.0 Hz, 2H), 7.79-7.80 (m, 4H), 7.97 (d, J = 8.5 Hz, 2H),

8.01 (d, J = 7.0 Hz, 2H), 8.18 (br, 2H), 8.49 (d, J = 8.0 Hz, 2H). ESI-TOF MS (H_2O): m/z 884.9 [**2**•(**Cor**)$_2$ – 4•NO_3^-]$^{4+}$, 1200.5 [**2**•(**Cor**)$_2$ – 3•NO_3^-]$^{3+}$.

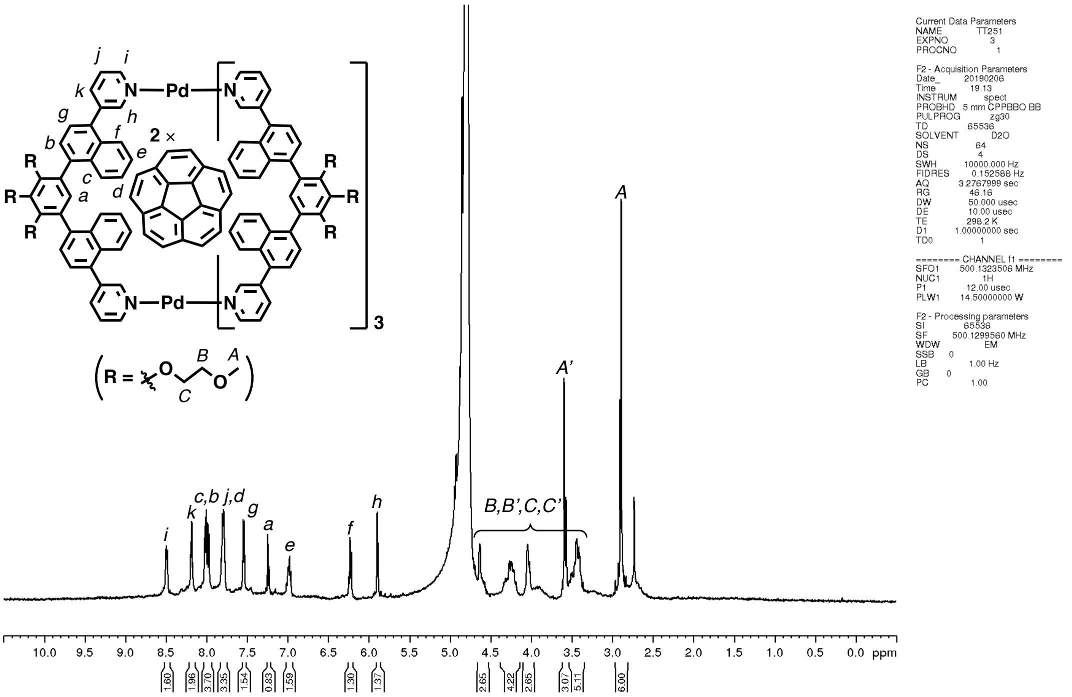

Figure S29a. ^{1}H NMR spectrum (500 MHz, D_2O, r.t.) of **2**•(**Cor**)$_2$.

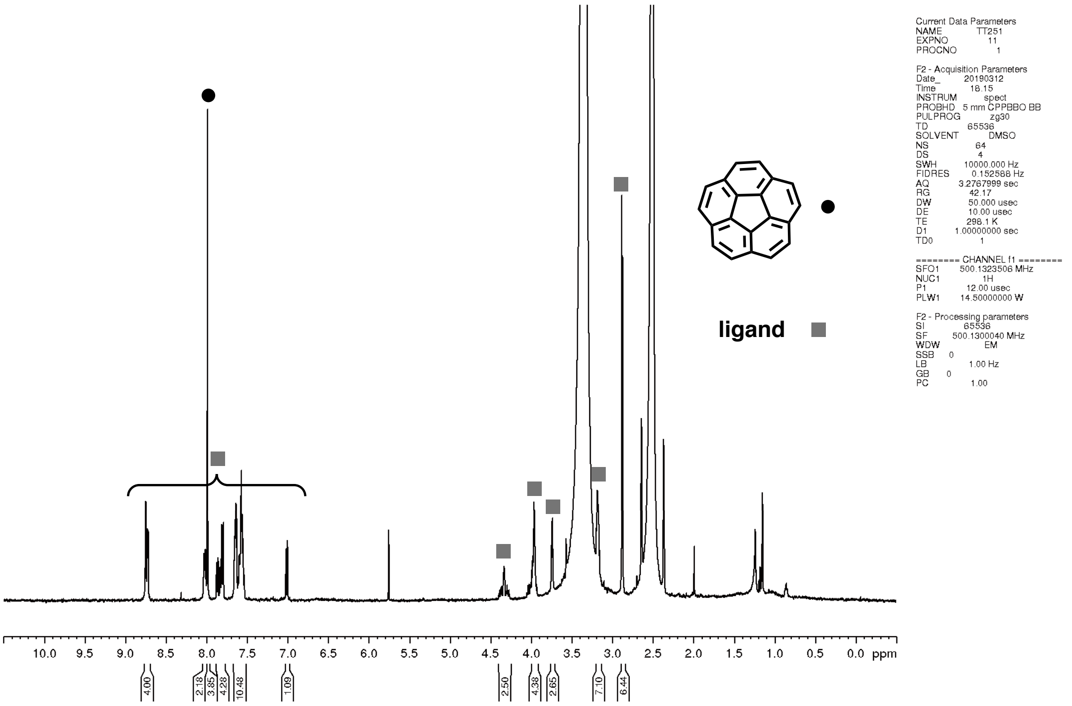

Figure S29b. ^{1}H NMR spectrum (500 MHz, DMSO-d_6, r.t.) of **2**•(**Cor**)$_2$ after disassembly.

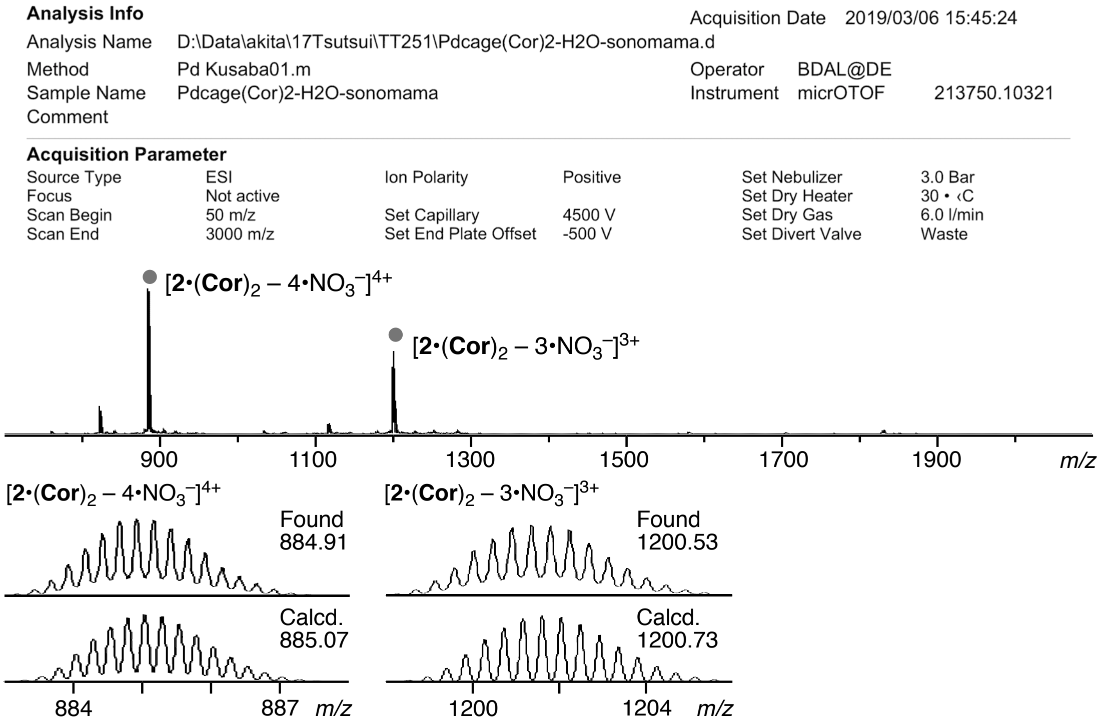

Figure S29c. ESI-TOF MS spectrum (H_2O) of **2•(Cor)**$_2$.

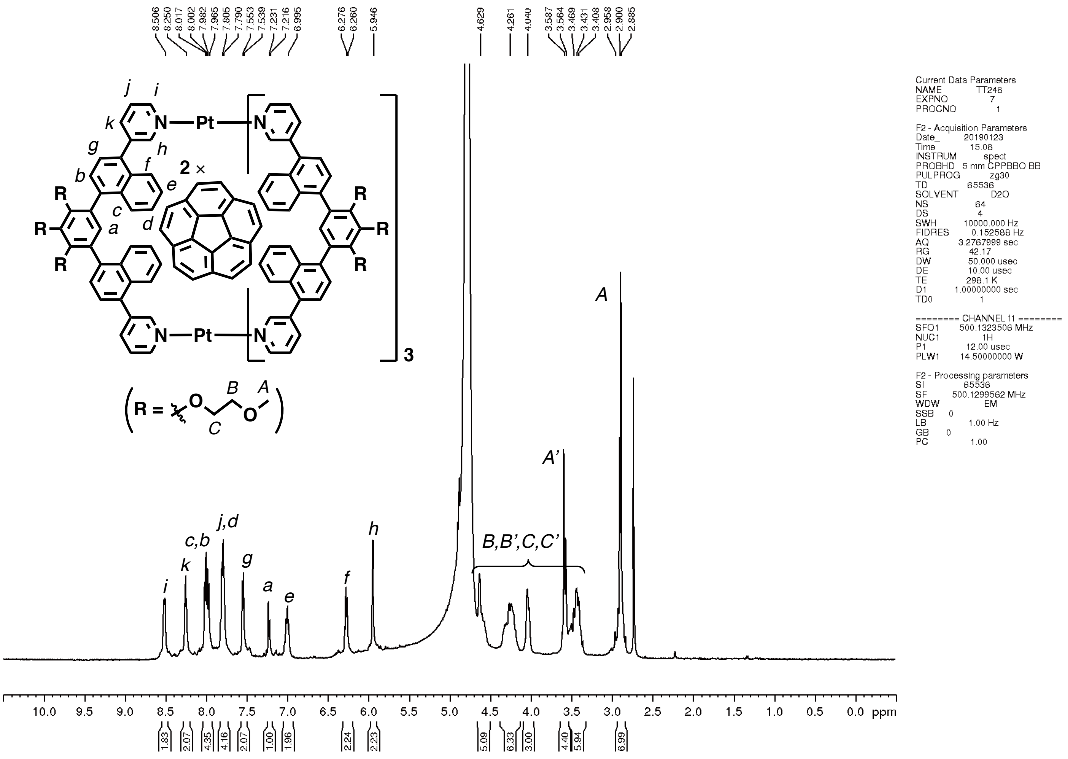

Figure S30a. ^{1}H NMR spectrum (500 MHz, D_2O, r.t.) of **2'•(Cor)**$_2$ (stirred at 80 ºC).

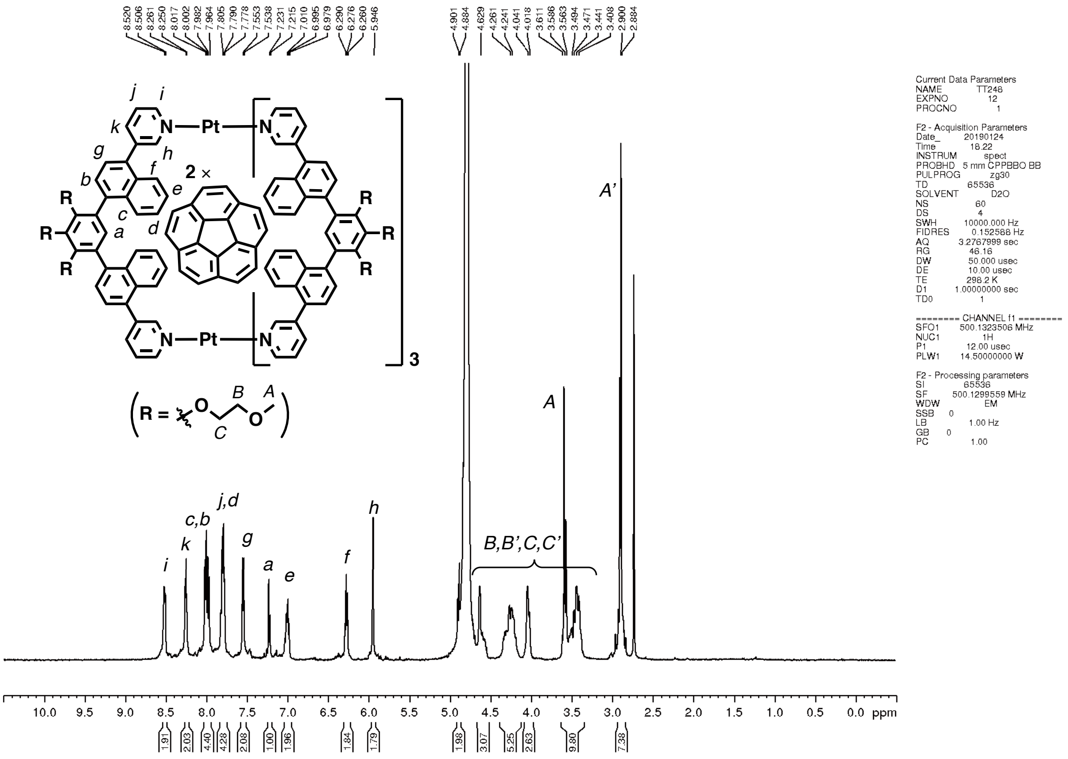

Figure S30b. ^{1}H NMR spectrum (500 MHz, D_2O, r.t.) of **2'•(Cor)**$_2$ (stirred at r.t.).

Analysis Info

Analysis Name	D:\Data\akita\17Tsutsui\TT248\diamantane-Ptcage.d	Acquisition Date	2019/01/25 23:51:29
Method	Pd Kusaba01.m	Operator	BDAL@DE
Sample Name	diamantane-Ptcage	Instrument	micrOTOF 213750.10321
Comment			

Acquisition Parameter

Source Type	ESI	Ion Polarity	Positive	Set Nebulizer	3.0 Bar
Focus	Not active			Set Dry Heater	30 • ‹C
Scan Begin	50 m/z	Set Capillary	4500 V	Set Dry Gas	6.0 l/min
Scan End	3000 m/z	Set End Plate Offset	-500 V	Set Divert Valve	Waste

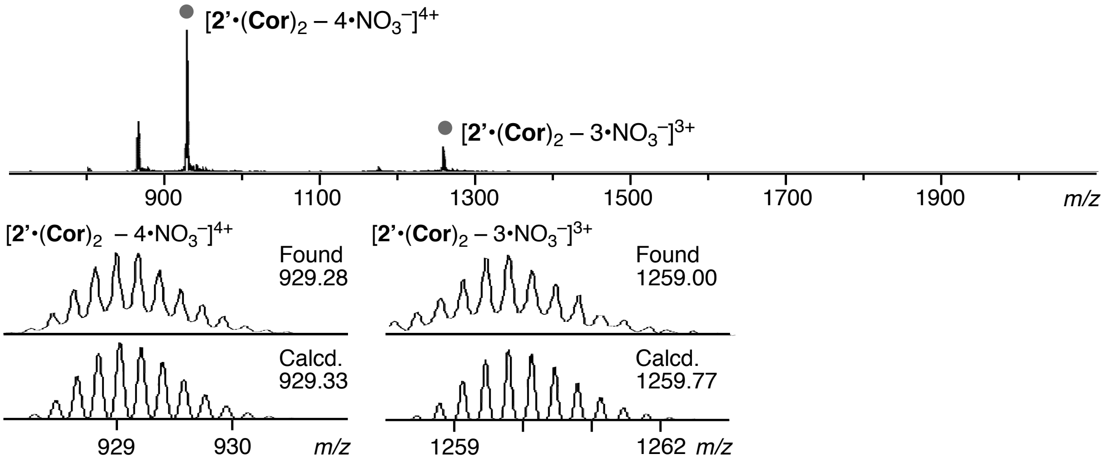

Figure S30c. ESI-TOF MS spectrum (H_2O) of **2'•(Cor)**$_2$.

Preparation of 2•C60   TT248, 236, 257

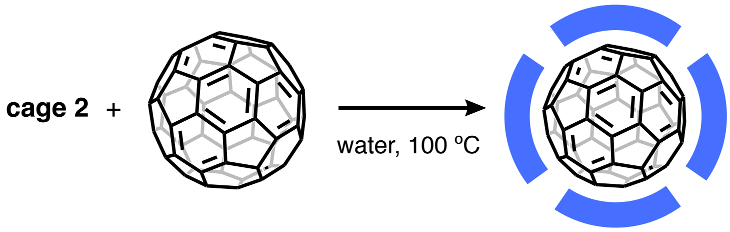

Pd(II)-linked cage **2** (1.0 mg, 0.3 μmol), fullerene (**C**$_{60}$; excess), and D_2O (0.5 mL) were added to a glass test tube. The mixture was stirred at 100 °C for 24 h. The quantitative formation of 1:1 host-guest complex **2•C**$_{60}$ was confirmed by NMR, ESI-TOF MS, and UV-visible analyses. Brown block crystals of **2•C**$_{60}$ were obtained by slow concentration of a 2:1 acetonitrile/water solution (2.1 mM) of **2•C**$_{60}$ over 3 weeks at r.t.

^{1}H NMR (500 MHz, D_2O, r.t.): δ 2.76 (s, 24H), 3.17 (br, 8H) 3.22 (br, 8H), 3.46 (s, 12H), 3.88-3.93 (br, 16 H), 4.01 (br, 8H), 4.42 (br, 8H), 6.94 (d, J = 8.5 Hz, 8H), 7.03 (s, 4H), 7.28 (br, 8H), 7.46 (d, J = 8.5 Hz, 8H), 7.52 (d, J = 7.5 Hz, 8H), 7.57 (br, 8H), 7.62 (d, J = 7.0 Hz, 8H), 7.97 (s, 8H), 8.19 (br, 8H), 8.83 (d, J = 8.0 Hz, 8H), 8.95 (d, J = 5.5 Hz, 8H). ^{13}C NMR (120 MHz, $CDCl_3$, r.t.): δ 140.1 (**C**$_{60}$). ESI-TOF MS (H_2O): m/z 940.2 [**2•C**$_{60}$ – 4•NO_3^-]$^{4+}$, 1274.3 [**2•C**$_{60}$ – 3•NO_3^-]$^{3+}$, 1941.9 [**2•C**$_{60}$ – 2•NO_3^-]$^{2+}$.

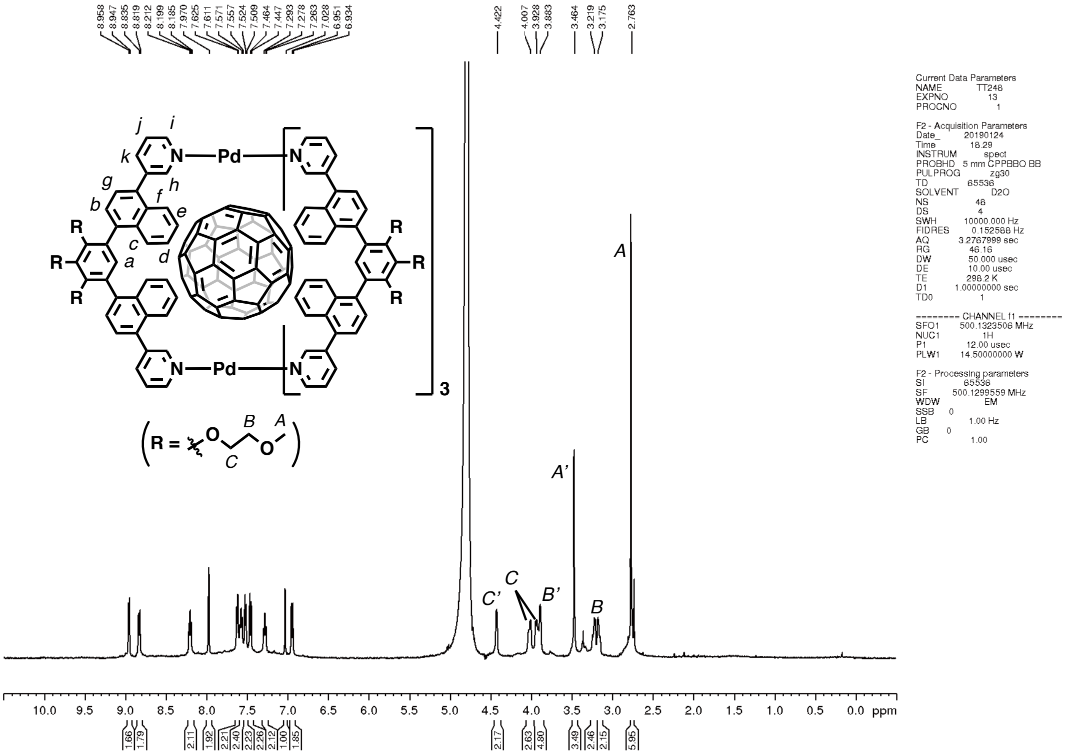

Figure S31a. ^{1}H NMR spectrum (500 MHz, D_2O, r.t.) of **2•C**$_{60}$.

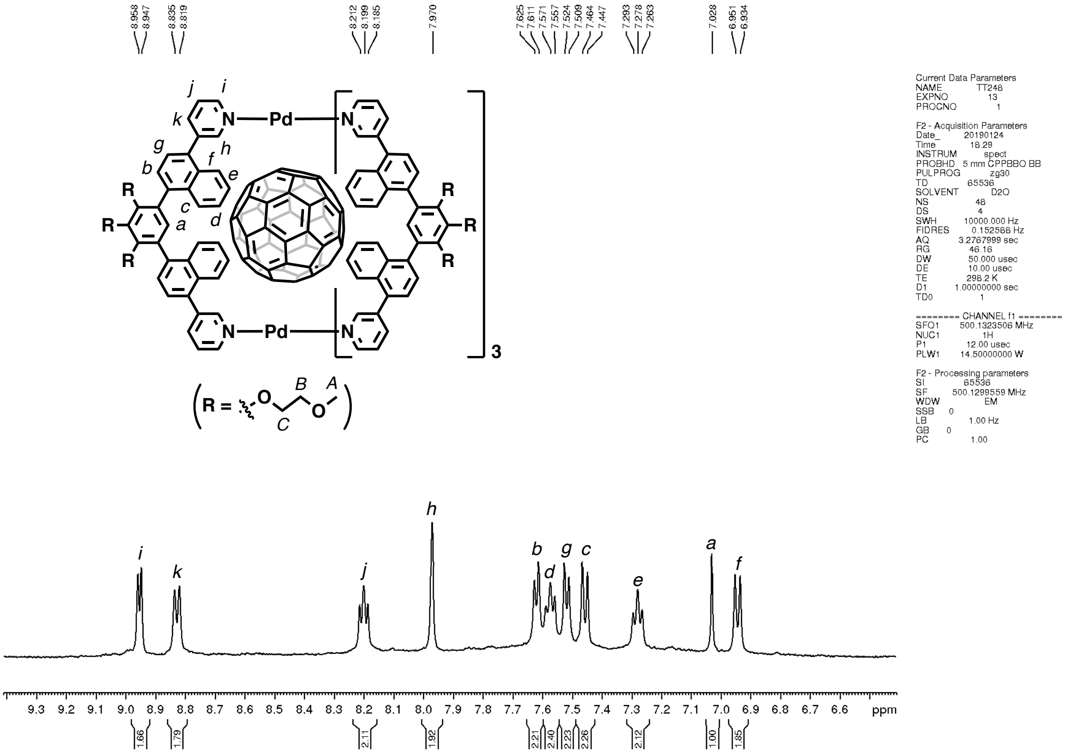

Figure S31b. ^{1}H NMR spectrum (500 MHz, D_2O, r.t.) of **2•C$_{60}$**.

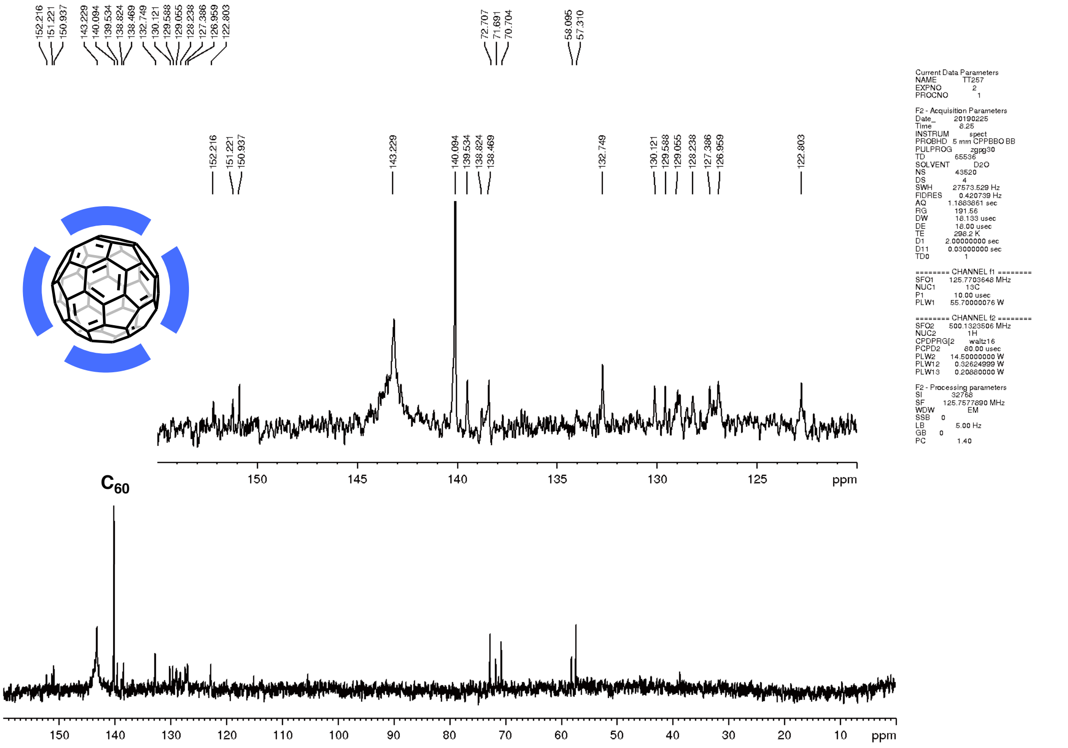

Figure S32. ^{13}C NMR spectrum (125 MHz, D_2O, r.t.) of **2•C$_{60}$**

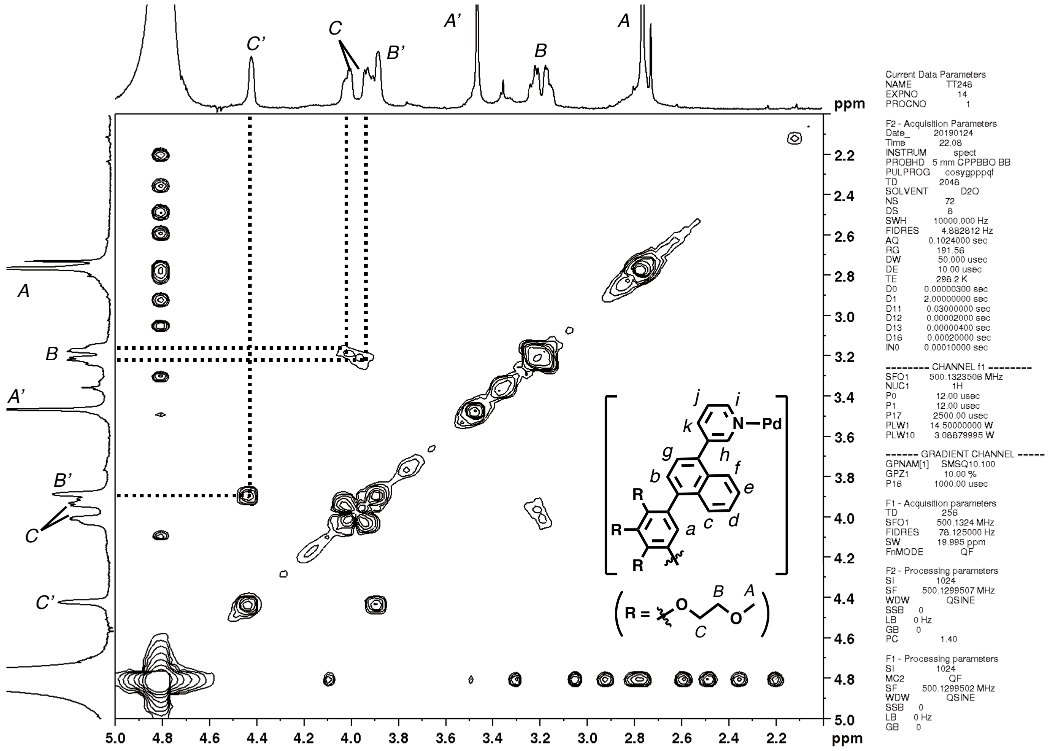

Figure S33a. ^{1}H-^{1}H COSY spectrum (500 MHz, D_2O, r.t.) of **2•C$_{60}$**.

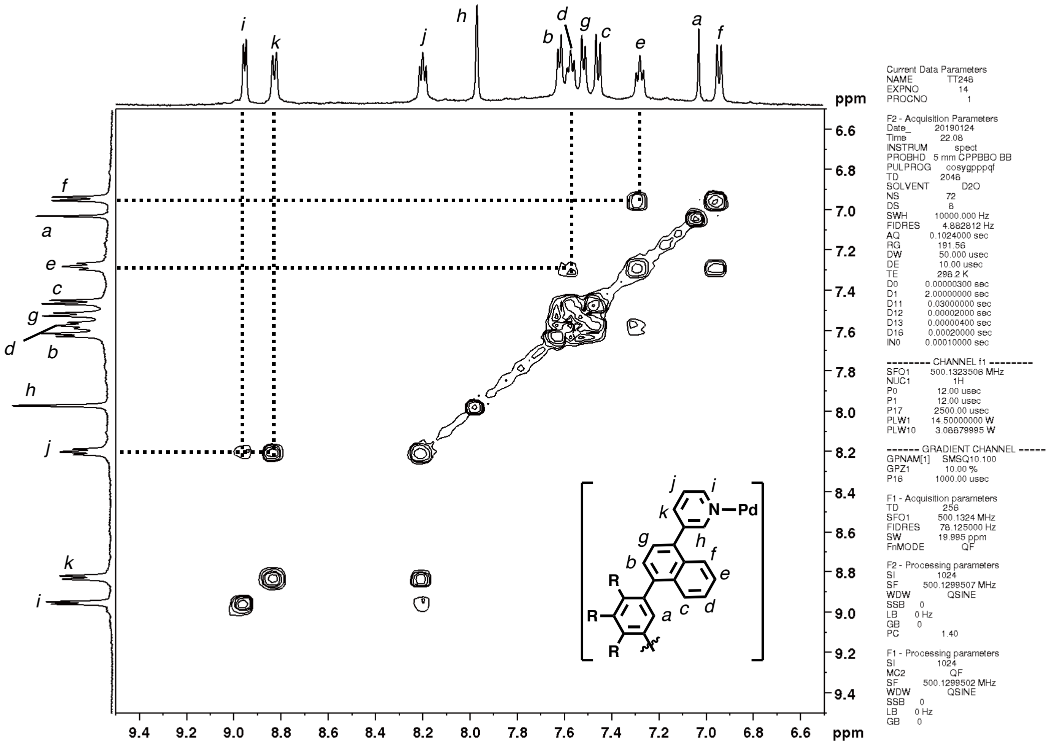

Figure S33b. ^{1}H-^{1}H COSY spectrum (500 MHz, D_2O, r.t.) of **2•C$_{60}$**.

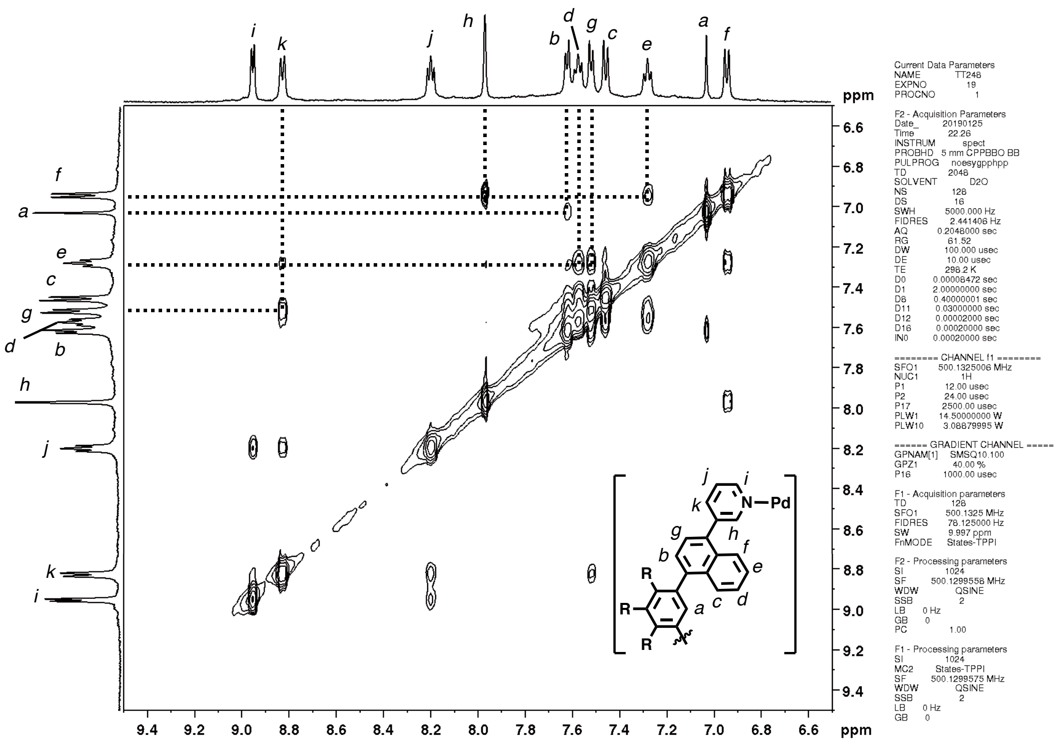

Figure S34a. NOESY NMR spectrum (500 MHz, D_2O, r.t.) of **2•C_{60}**.

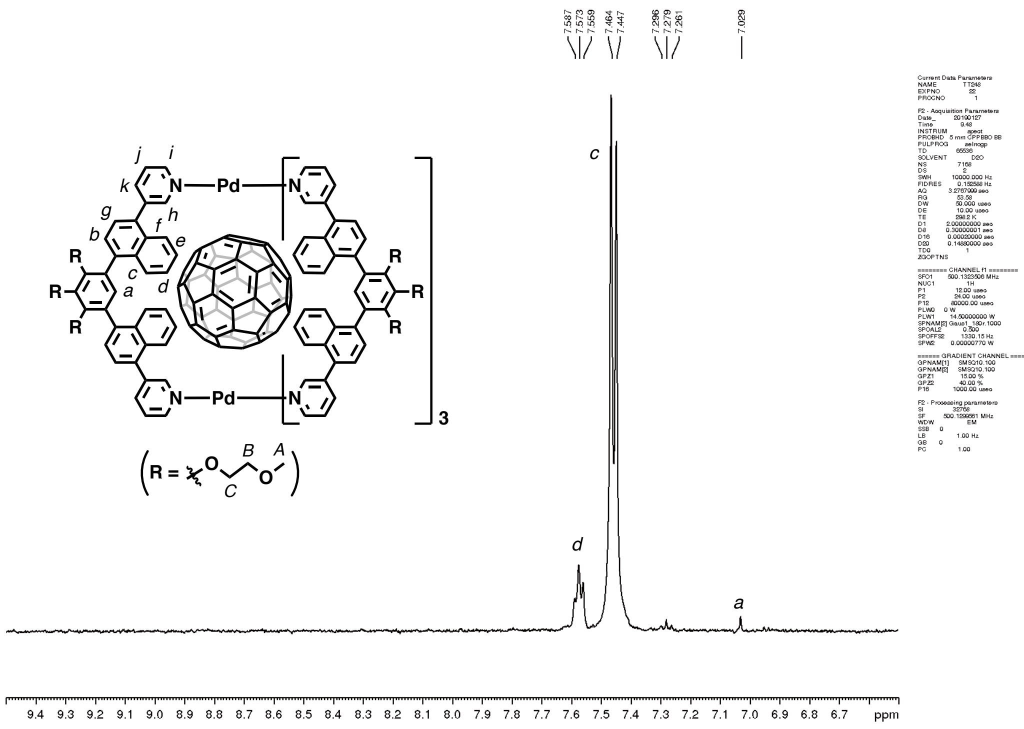

Figure S34b. 1D NOESY NMR spectrum (500 MHz, D_2O, r.t.) of **2•C_{60}** (irradiated at 7.36 ppm)

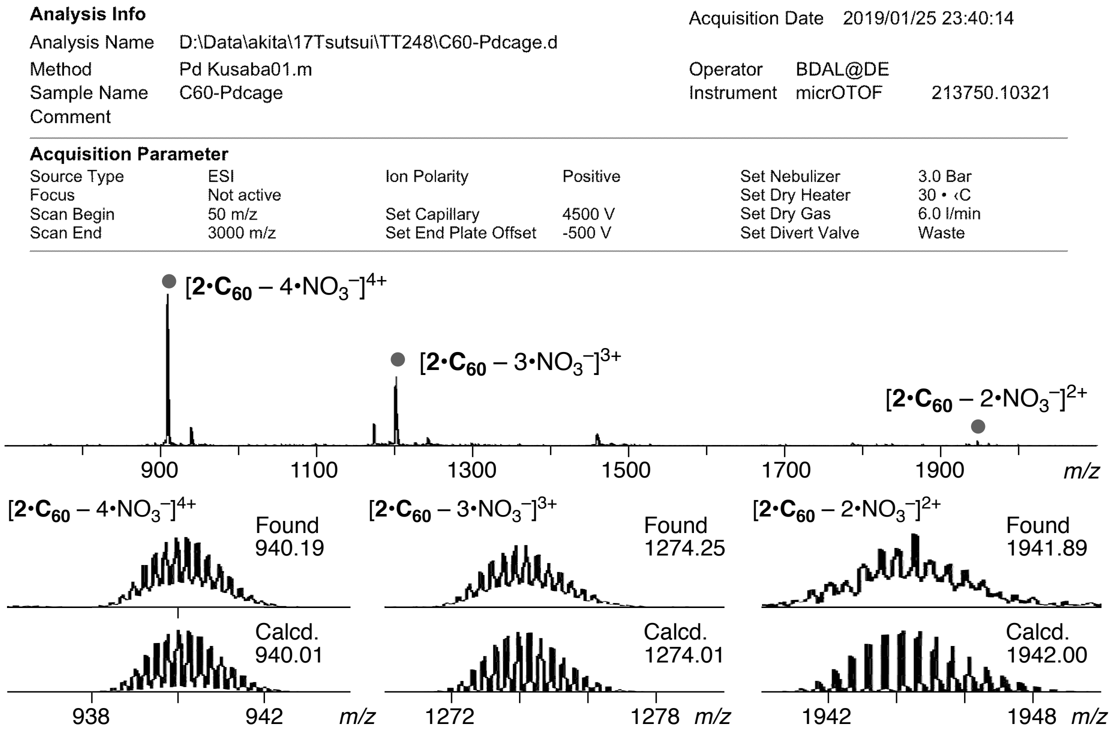

Figure S35. ESI-TOF MS spectrum (H_2O) of **2•C$_{60}$**.

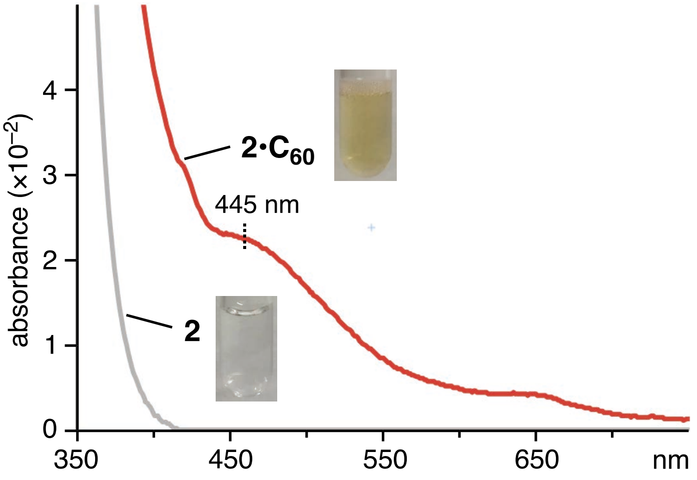

Figure S36. UV-visible spectra (r.t., H_2O, 0.2 mM based on **2**) of **2•C$_{60}$** and **2**.

Table S1. Crystal data and structure refinement for **2•C_{60}**.

Identification code	TT236	
Empirical formula	C240 H168 N12 O38 Pd2	
Formula weight	4040.66	
Temperature	223(1) K	
Wavelength	0.71073 Å	
Crystal system	Tetragonal	
Space group	I-4	
Unit cell dimensions	*a* = 20.0773(5) Å	α = 90°
	b = 20.0773(5) Å	β = 90°
	c = 23.9655(8) Å	γ = 90°
Volume	9660.4(6) $Å^3$	
Z	2	
Density (calculated)	1.389 Mg/m^3	
Absorption coefficient	0.270 mm^{-1}	
F(000)	4176	
Crystal size	0.091 x 0.080 x 0.049 mm^3	
Theta range for data collection	1.323 to 26.382°	
Index ranges	–21<=h<=25, –19<=k<=24, –29<=l<=29	
Reflections collected	26091	
Independent reflections	9442 [R(int) = 0.0300]	
Completeness to theta = 25.242°	100.0 %	
Absorption correction	Semi-empirical from equivalents	
Max. and min. transmission	0.987 and 0.73	
Refinement method	Full-matrix least-squares on F^2	
Data / restraints / parameters	9442 / 4400 / 1145	
Goodness-of-fit on F^2	1.332	
Final R indices [I>2sigma(I)]	R_1 = 0.0991, wR_2 = 0.2732	
R indices (all data)	R_1 = 0.1256, wR_2 = 0.3162	
Absolute structure parameter	0.48(7)	
Extinction coefficient	n/a	
Largest diff. peak and hole	2.467 and –0.582 e.$Å^{-3}$	

The supplementary crystallographic data (CCDC 1907426) can be obtained free of charge from the Cambridge Crystallographic Data Centre via www.ccdc.cam. ac.uk/data_request/cif.

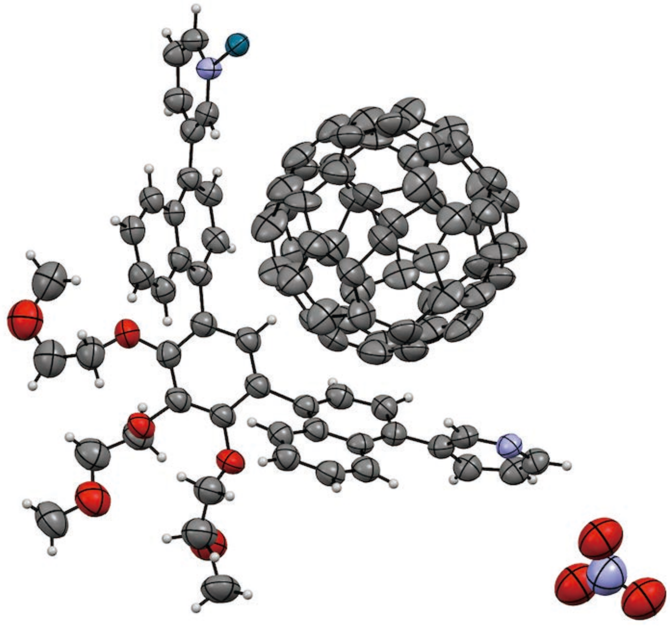

Figure S37. ORTEP drawing of **2•C$_{60}$**. The thermal ellipsoids are drawn at 50% probability.

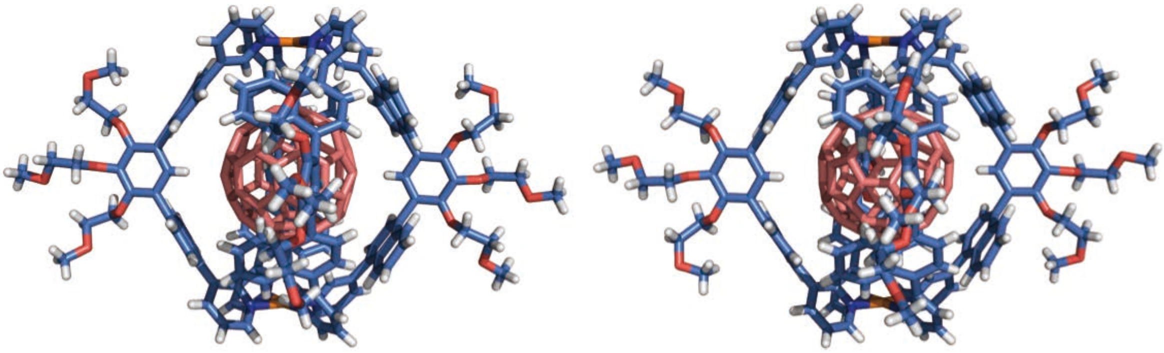

Figure S38a. Crystal structure (stick representation) of **2•C$_{60}$** (front and back sides). The anions and solvent molecules are omitted for clarity.

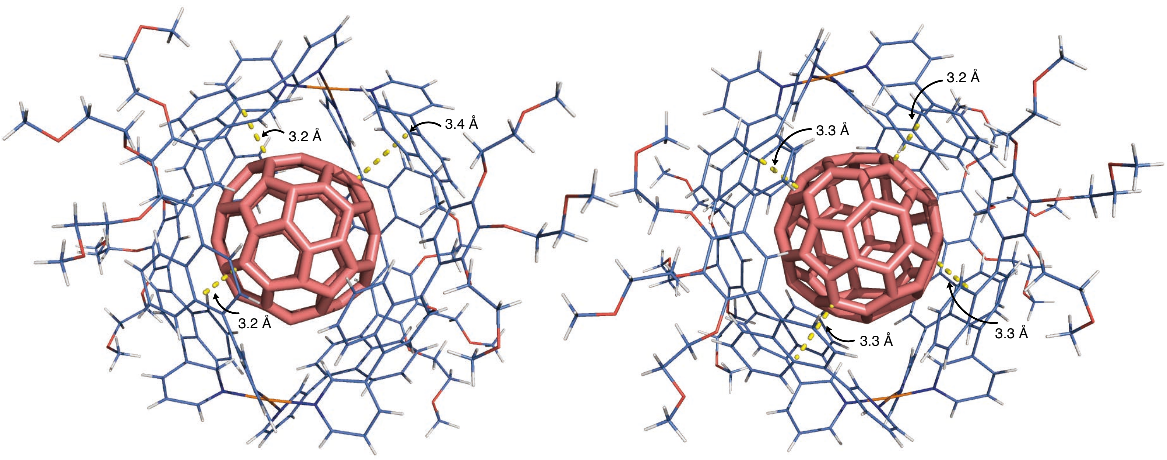

Figure S38b. Highlighted host-guest interactions within **2•C$_{60}$** (front and back sides, yellow dotted lines: π-π interactions).

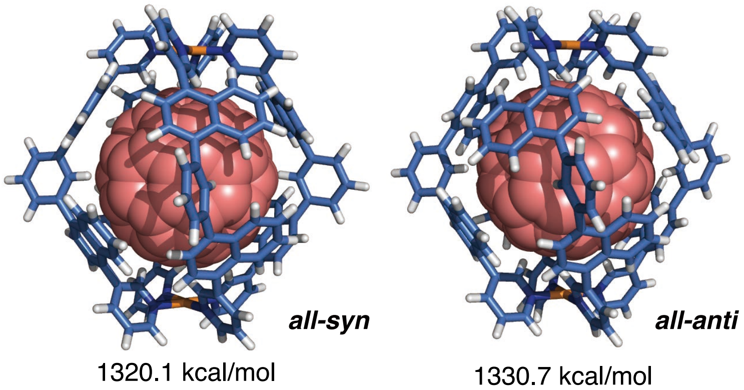

Figure S39. Optimized structures of **2•C$_{60}$** (two isomers; R = -H) and their energies.

Preparation of 2·MC$_{60}$ TT241

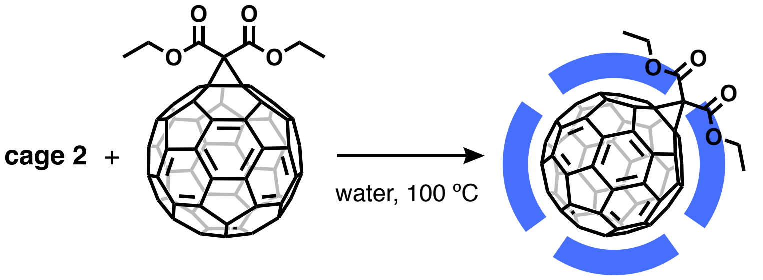

Pd(II)-linked cage **2** (1.0 mg, 0.3 μmol), fullerene derivative **MC$_{60}$** (excess), and D_2O (0.5 mL) were added to a glass test tube. The mixture was stirred at 100 ºC for 24 h. The selective formation of 1:1 host-guest complex **2•MC$_{60}$** was confirmed by NMR, ESI-TOF MS, and UV-visible analyses.

ESI-TOF MS (H_2O): *m/z* 979.6 [**2•MC$_{60}$** – 4•NO_3^-]$^{4+}$, 1326.8 [**2•MC$_{60}$** – 3•NO_3^-]$^{3+}$.

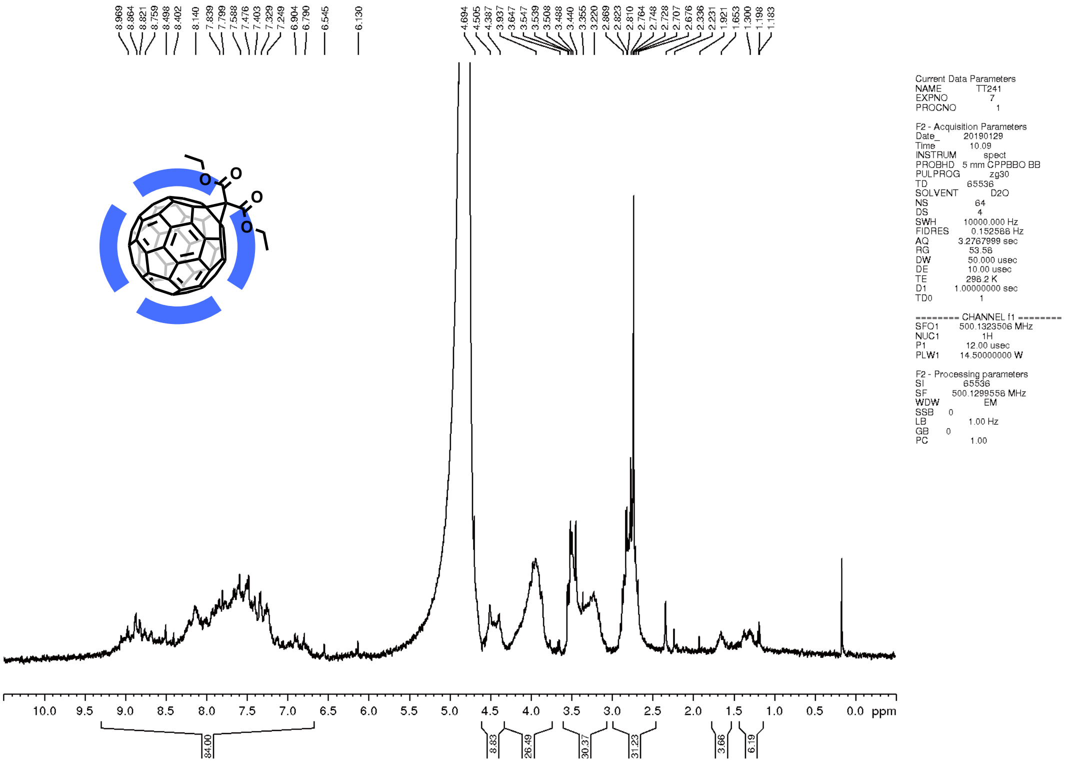

Figure S40a. ^{1}H NMR spectrum (500 MHz, D_2O, r.t.) of **2•MC$_{60}$**.

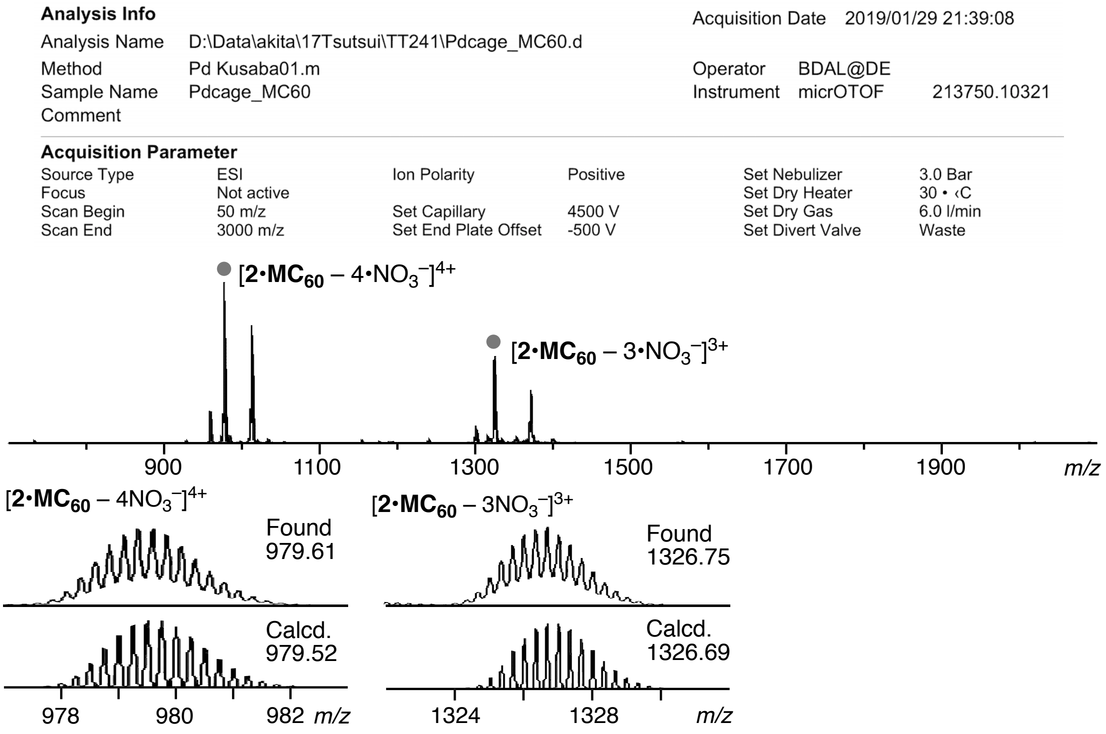

Figure S40b. ESI-TOF MS spectrum (H_2O) of **2•MC$_{60}$**.

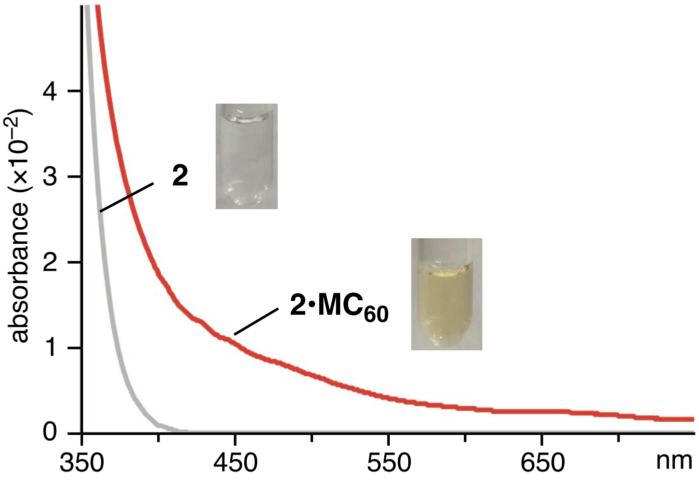

Figure S40c. UV-visible spectra (r.t., H_2O, 0.2 mM based on **2**) of **2** and **2•MC$_{60}$**.

Preparation of 2'•PMB and 3•PMB   LC537, 541, 542

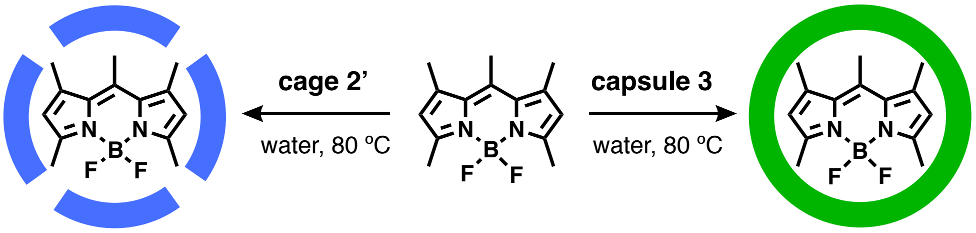

To a solution of Pt(II)-linked cage **2'** (1.1 mg, 0.3 μmol) in D_2O (1.1 mL) was added pentamethyl boron-dipyrromethene **PMB** (0.7 mg, 2.7 μmol) and the mixture was stirred at 80 °C for 90 min in a glass test tube. After filtration of the resultant suspension, the formation of host-guest complex **2'•PMB** was confirmed by NMR and ESI-TOF MS analyses. The UV-visible, fluorescence, and quantum yield analyses of **2'•PMB** were performed at a concentration of 80 μM after dilution with H_2O. Similarly, a mixture of Pt(II)-linked capsule **3** (1.4 mg, 0.4 μmol), **PMB** (0.8 mg, 2.9 μmol), and D_2O (1.2 mL) was stirred at 80 ºC for 90 min.[S2] The formation of 1:1 host-guest complex **3•PMB** was confirmed by NMR, UV-visible, and fluorescence analyses.

2'•PMB: ESI-TOF MS (H_2O): *m/z* 869.8 [**2'•PMB** – 4•NO_3^-]$^{4+}$, 1180.4 [**2'•PMB** – 3•NO_3^-]$^{3+}$.

3•PMB: ^{1}H NMR (400 MHz, D_2O, r.t.): δ –1.21 to –0.96 (m, 15H), 1.99 (br, 2H), 2.50 (s, 24H), 3.06-3.20 (m, 16H), 3.51 (s, 12H), 3.93-4.00 (m, 8H), 4.00-4.15 (m, 16H), 4.46-4.54 (m, 4H), 4.59-4.68 (m, 4H), 6.08 (s, 4H), 6.60 (d, *J* = 8.8 Hz, 8H), 6.84-6.97 (m, 16H), 7.31 (pt, *J* = 8.8 Hz, 8H), 7.49 (pt, *J* = 8.5 Hz, 8H), 7.63 (br, 8H), 7.74 (d, *J* = 8.5 Hz, 8H), 7.80 (pt, *J* = 7.0 Hz, 8H), 8.01 (d, *J* = 8.5 Hz, 8H), 8.34 (pt, *J* = 7.0 Hz, 8H), 8.61 (d, *J* =

8.0 Hz, 8H), 9.15 (d, $J = 4.4$ Hz, 8H).

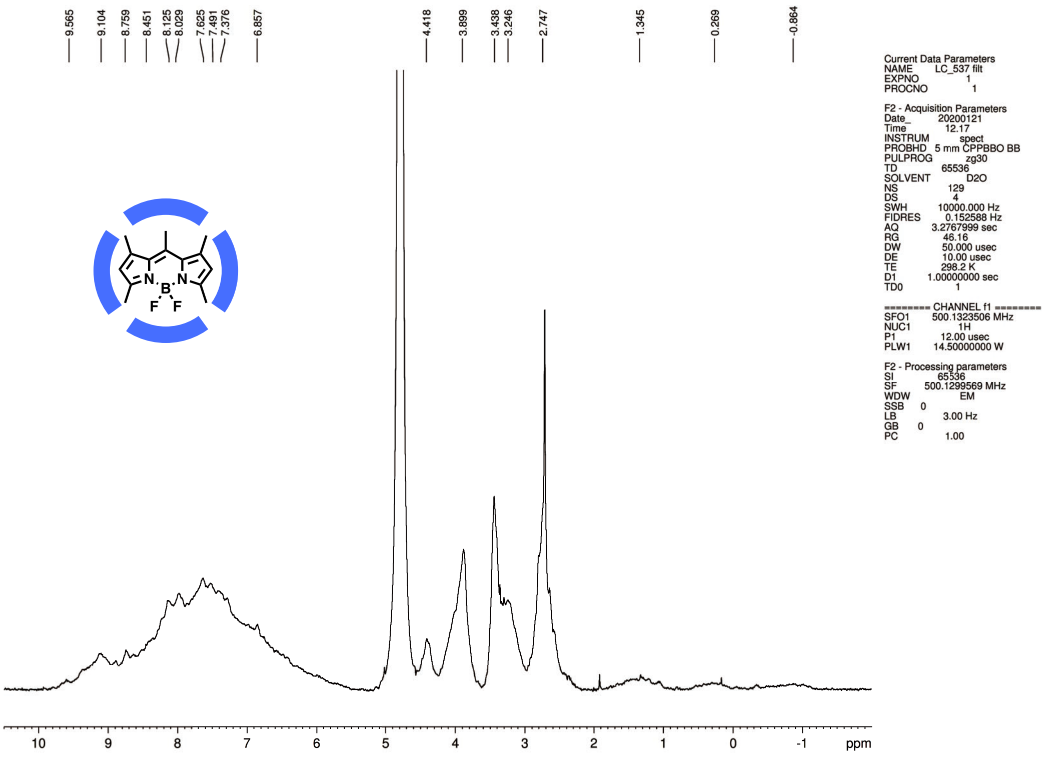

Figure S41a. ^{1}H NMR spectrum (500 MHz, D_2O, r.t.) of **2'•PMB**.

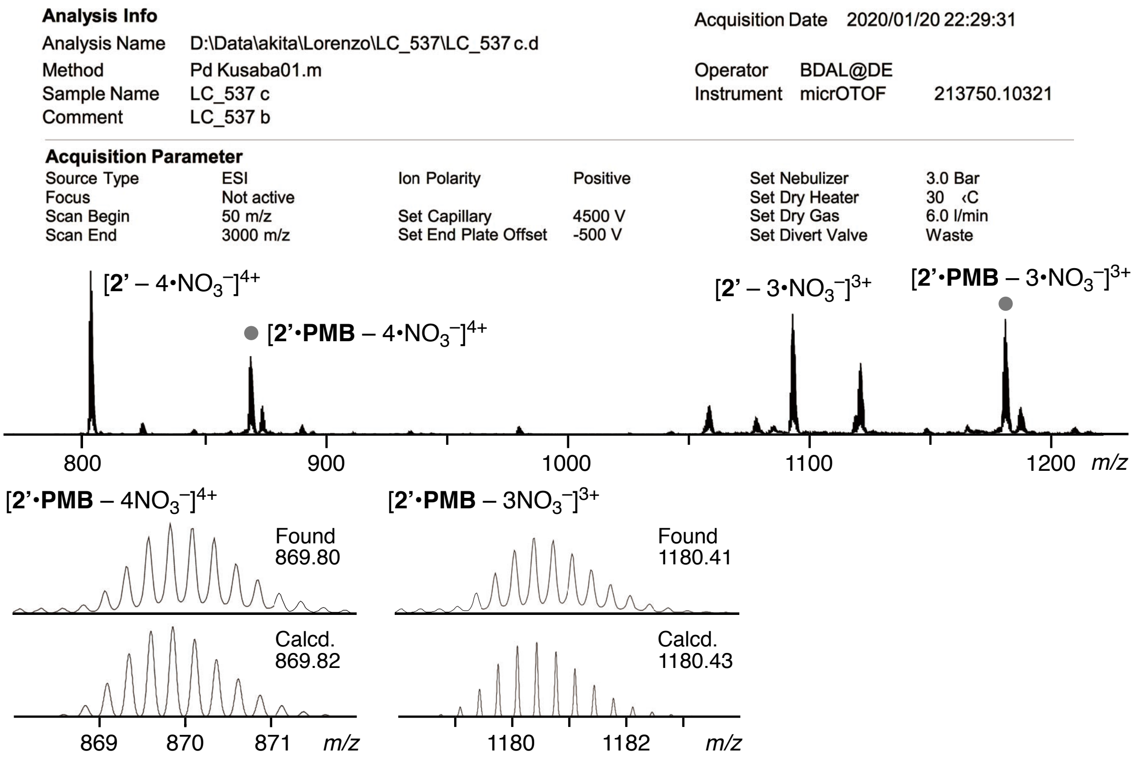

Figure S41b. ESI-TOF MS spectrum (H_2O) of **2'•PMB**.

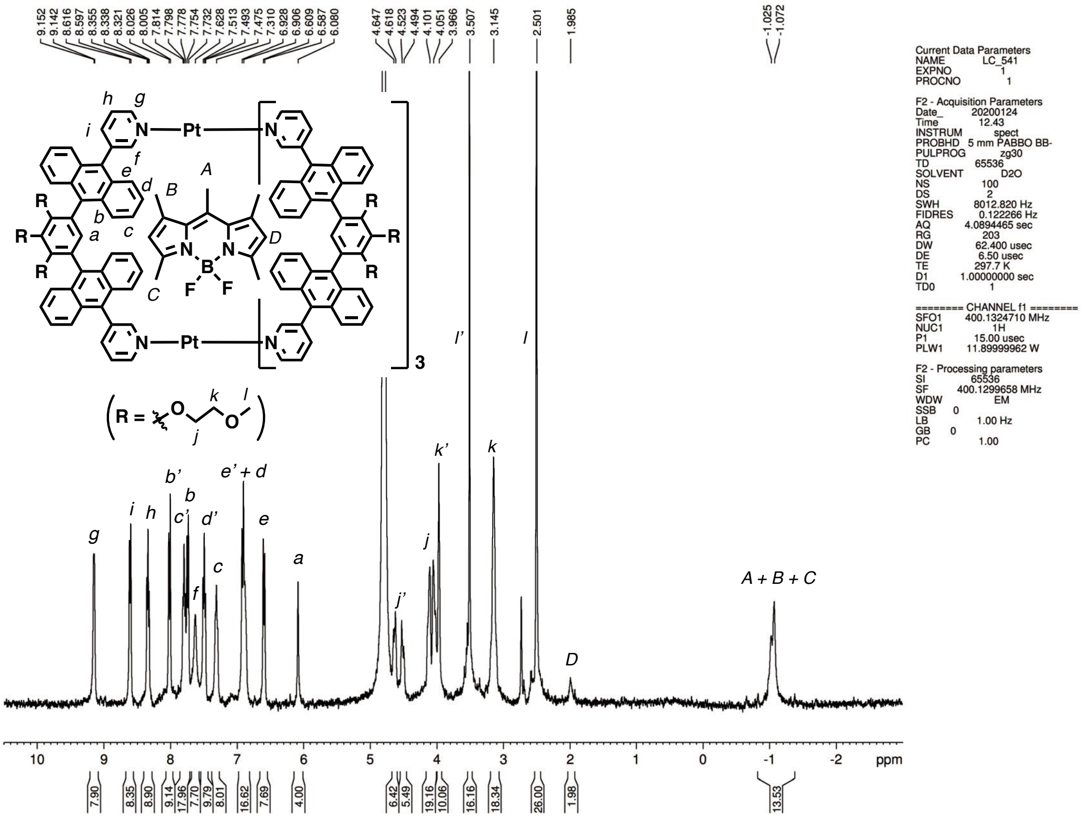

Figure S42. ^{1}H NMR spectrum (400 MHz, D_2O, r.t.) of **3•PMB**.

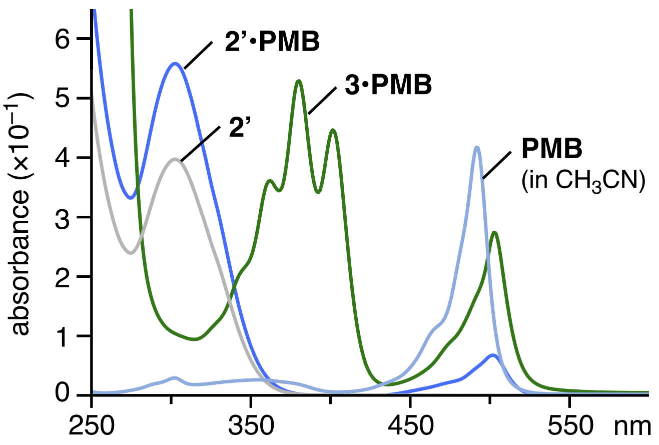

Figure S43a. UV-visible spectra (H_2O, r.t., 80 μM based on host) of **2’**, **2’•PMB**, **3•PMB**, and **PMB** (CH_3CN, r.t., 40 μM).

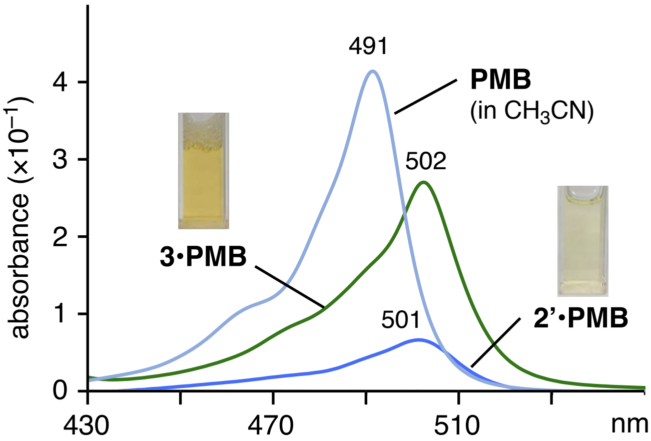

Figure S43b. UV-visible spectra (H_2O, r.t., 80 μM based on host) of **2'•PMB**, **3•PMB**, and **PMB** (CH_3CN, r.t., 40 μM).

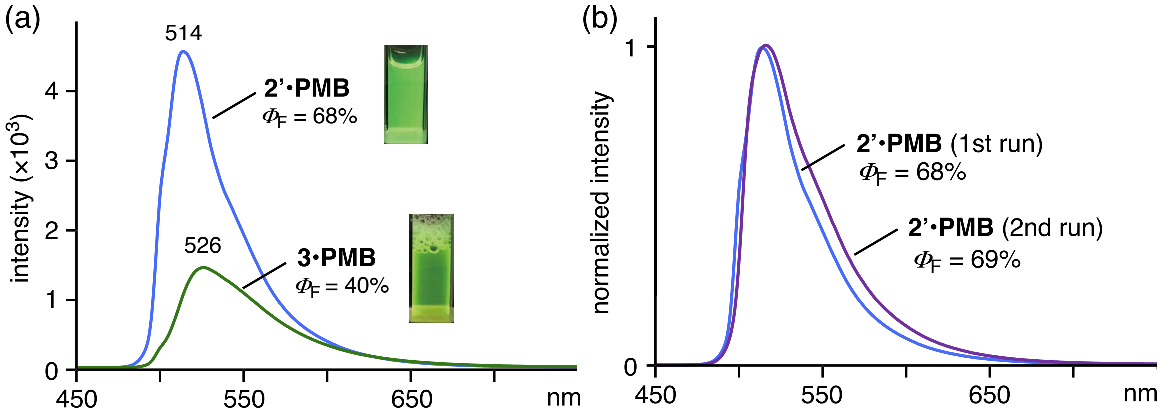

Figure S44. (a) Fluorescence spectra (H_2O, r.t., 80 μM based on host, λ_{ex} = 500 nm) of **2'•PMB** and **3•PMB**, and their photographs (λ_{ex} = 365 nm). (b) Fluorescence spectra (H_2O, r.t., 80 μM based on **2'**, 1st run: λ_x = 500 nm; 2nd run: λ_{ex} = 504 nm) of two separately prepared **2'•PMB** samples.
